# Screening of herbal extracts for TLR2- and TLR4-dependent anti-inflammatory effects

**DOI:** 10.1371/journal.pone.0203907

**Published:** 2018-10-11

**Authors:** Anne Schink, Jan Neumann, Anna Lena Leifke, Kira Ziegler, Janine Fröhlich-Nowoisky, Christoph Cremer, Eckhard Thines, Bettina Weber, Ulrich Pöschl, Detlef Schuppan, Kurt Lucas

**Affiliations:** 1 Multiphase Chemistry Department, Max Planck Institute for Chemistry, Mainz, Germany; 2 Institute of Molecular Biology, Mainz, Germany; 3 Institut für Biotechnologie und Wirkstoff-Forschung gGmbH, Kaiserslautern, Germany; 4 Institute of Molecular Physiology, Johannes Gutenberg University Mainz, Mainz, Germany; 5 Institute of Translational Immunology, University of Mainz Medical Center, Mainz, Germany; 6 Division of Gastroenterology, Beth Israel Deaconess Medical Center, Harvard Medical School, Boston, MA, United States of America; University of the Pacific, UNITED STATES

## Abstract

Herbal extracts represent an ample source of natural compounds, with potential to be used in improving human health. There is a growing interest in using natural extracts as possible new treatment strategies for inflammatory diseases. We therefore aimed at identifying herbal extracts that affect inflammatory signaling pathways through toll-like receptors (TLRs), TLR2 and TLR4. Ninety-nine ethanolic extracts were screened in THP-1 monocytes and HeLa-TLR4 transfected reporter cells for their effects on stimulated TLR2 and TLR4 signaling pathways. The 28 identified anti-inflammatory extracts were tested in comparative assays of stimulated HEK-TLR2 and HEK-TLR4 transfected reporter cells to differentiate between direct TLR4 antagonistic effects and interference with downstream signaling cascades. Furthermore, the ten most effective anti-inflammatory extracts were tested on their ability to inhibit nuclear factor-κB (NF-κB) translocation in HeLa-TLR4 transfected reporter cell lines and for their ability to repolarize M1-type macrophages. Ethanolic extracts which showed the highest anti-inflammatory potential, up to a complete inhibition of pro-inflammatory cytokine production were *Castanea sativa* leaves, *Cinchona pubescens* bark, *Cinnamomum verum* bark, *Salix alba* bark, *Rheum palmatum* root, *Alchemilla vulgaris* plant, *Humulus lupulus* cones, *Vaccinium myrtillus* berries, *Curcuma longa* root and *Arctostaphylos uva-ursi* leaves. Moreover, all tested extracts mitigated not only TLR4, but also TLR2 signaling pathways. Seven of them additionally inhibited translocation of NF-κB into the nucleus. Two of the extracts showed impact on repolarization of pro-inflammatory M1-type to anti-inflammatory M2-type macrophages. Several promising anti-inflammatory herbal extracts were identified in this study, including extracts with previously unknown influence on key TLR signaling pathways and macrophage repolarization, serving as a basis for novel lead compound identification.

## Introduction

Herbs, algae, fungi and cyanobacteria have been used in traditional medicine for centuries. During the last decades, plant extracts and natural compounds became a focal point of interest again as novel lead compounds for the treatment of inflammatory diseases are needed [1]. Several diseases progression and development are influenced by acute and chronic inflammation, such as: autoimmune diseases, allergies, obesity, diabetes, organ fibrosis and dysfunction. Plant extracts that contain largely orally available compounds which attenuate inflammatory processes may be highly attractive as potential therapies [2–8]. Regardless of the origin, inflammation is often associated with a self-enhancing, cyclic process, involving stimulation of innate immunity, prominently of TLRs, production of reactive oxygen and nitrogen species (ROS/RNS), pro-inflammatory cytokine/chemokine secretion, as well as the release of host-derived damage associated molecular patterns (DAMPs) [9,10]. In healthy individuals the initial immune response to an acute stimulus, e.g. a microbial infection, is mitigated over time by downregulation of TLR stimulation, leading to a diminished cytokine production and interruption of the vicious inflammatory circle. In diseases associated with chronic inflammation, the appropriate regulation of TLRs and their downstream signaling pathways is often absent [1, 11]. Antagonists for TLR signaling play an important role in counter-regulating such overwhelming reactions, especially for TLR4 which is a central danger-sensing innate immune receptor. Different from all other TLRs, stimulation of TLR4, leads to activation of two major pathways: 1) the myeloid differentiation 88-dependent (MyD88) or canonical pathway of NF-κB activation, and 2) the MyD88-independent or Toll/interleukin-1 receptor (TIR)-domain-containing adaptor molecule (TRAM) pathway. The canonical pathway can also be activated via TLR2 stimulation [12,13]. Some synthetic small molecules (e.g. Eritoran and TAK-242), but also natural compounds (e.g. epigallocatechin-3-gallate and 6-shogaol) inhibit TLR4 signaling [14–18]. Nevertheless, to date, no effective orally active TLR4 antagonist is available for experimental or clinical application.

Due to their easy oral application and minor adverse effects, herbal extracts compromising of TLR4 antagonistic activity would be highly interesting as new oral treatment strategies for inflammatory diseases. Nevertheless, identification of the active compounds and their targets are often complex. Furthermore, also metabolization products and not only the applied compounds themselves might interact with the TLR signaling pathways. This further complicates the identification of the responsible mechanism(s). Recently, numerous studies have focused on Chinese herbal medicines and their impact on several diseases [19–22], however, their anti-inflammatory effects remain largely unknown. Thus, in the current study we analyzed ethanolic extracts of medicinal plants, which may have anti-inflammatory properties (see S1 Table in the supplementary data).

## Materials and methods

### Ethanolic extracts

Most of the ethanolic extracts were purchased directly from Maros Arznei GmbH. All other samples listed in Table 1 were freshly prepared by grinding 10 g plants or algae in a mortar, if necessary under liquid nitrogen. Afterwards, the powder was resuspended in 50 ml of 70% ethanol (VWR International GmbH, Darmstadt, Germany). Subsequently, the samples were incubated for ten days in the dark at room temperature (RT), being inverted once a day. Then, the extracts were filtered through Rotilabo-folded filters (type 113P; Carl Roth, Karlsruhe, Germany) to remove unresolved residues.

**Table 1:** Sources of ethanolic herbal extracts.

Latin name	Common English name	Used part	Source
*Achillea millefolium*	Common yarrow	Whole plant	Maros Arznei, Fürth, Germany
*Aconitum napellus*	Monkshood	Whole plant	Maros Arznei, Fürth, Germany
*Aesculus hippocastanum*	Horse-chestnut	Fruit/berry/seed	Maros Arznei, Fürth, Germany
*Alchemilla vulgaris*	Common lady's mantle	Whole plant	Maros Arznei, Fürth, Germany
*Allium sativum*	Garlic	Root^2^	Farmer's market, Mainz, Germany
*Allium ursinum*	Wild garlic	Leaf^1^	Tee und Gewürze Lilianna Kamberg und Marianne Schmidt, Offenbach, Germany
*Aloe ferox*	Aloe	Whole plant	Maros Arznei, Fürth, Germany
*Alpinia officinarum*	Galangal	Root	Maros Arznei, Fürth, Germany
*Althaea officinalis*	Common marshmallow	Root	Maros Arznei, Fürth, Germany
*Arctostaphylos uva-ursi*	Bearberry	Leaf	Maros Arznei, Fürth, Germany
*Armoracia rusticana*	Horseradish	Root^2^	Farmer's market, Mainz, Germany
*Arnica montana*	Arnica	Whole plant	Maros Arznei, Fürth, Germany
*Arnica montana*	Arnica	Flower	Maros Arznei, Fürth, Germany
*Artemisia absinthium*	Wormwood	Whole plant	Maros Arznei, Fürth, Germany
*Avena sativa*	Oat	Whole plant	Maros Arznei, Fürth, Germany
*Betula alba*	Birch	Juice/resin	Maros Arznei, Fürth, Germany
*Betula verrucosa*	Weeping birch	Juice/resin	Maros Arznei, Fürth, Germany
*Boswellia carterii*	Frankincense	Whole plant^1^	Olibanum B.V., Kerkrade, Netherlands
*Boswellia serrata*	Frankincense	Juice/resin	Maros Arznei, Fürth, Germany
*Calendula officinalis*	Marigold	Flower	Maros Arznei, Fürth, Germany
*Camellia sinensis* (L.)	Green tea	Leaf^1^	Ostfriesische Tee Gesellschaft, Seevetal, Germany
*Capsicum frutescens*	Chili	Fruit/berry/seed^1^	Tee und Gewürze Lilianna Kamberg und Marianne Schmidt, Offenbach, Germany
*Carum carvi*	Caraway	Fruit/berry/seed	Maros Arznei, Fürth, Germany
*Castanea sativa*	Sweet chestnut	Leaf	Maros Arznei, Fürth, Germany
*Chelidonium majus*	Celandine	Root	Maros Arznei, Fürth, Germany
*Chlorella pyrenoidosa*	Chlorella	Whole green algae^1^	Naturya, Southstoke, United Kingdom
*Cinchona pubescens*	Cinchona	Bark	Maros Arznei, Fürth, Germany
*Cinnamomum verum*	Cinnamon	Bark	Maros Arznei, Fürth, Germany
*Convallaria majalis*	Lily of the valley	Whole plant	Maros Arznei, Fürth, Germany
*Coriandrum sativum*	Coriander	Fruit/berry/seed	Maros Arznei, Fürth, Germany
*Crataegus species*	Hawthorn	Fruit/berry/seed	Maros Arznei, Fürth, Germany
*Curcuma longa*	Turmeric	Root	Maros Arznei, Fürth, Germany
*Cynara scolymus*	Artichoke	Leaf	Maros Arznei, Fürth, Germany
*Daucus carota* subsp. *sativus*	Carrot	Root^2^	Aldi Süd, Mainz, Germany
*Digitalis purpurea*	Common foxglove	Leaf	Maros Arznei, Fürth, Germany
*Dioscorea villosa*	Yam	Root	Maros Arznei, Fürth, Germany
*Echinacea purpurea*	Purple coneflower	Whole plant	Maros Arznei, Fürth, Germany
*Elettaria cardamomum*	Cardamom	Fruit/berry/seed^1^	Tee und Gewürze Lilianna Kamberg und Marianne Schmidt, Offenbach, Germany
*Equisetum arvense*	Field horsetail	Whole plant	Maros Arznei, Fürth, Germany
*Erythraea centaurium*	Common centaury	Whole plant	Maros Arznei, Fürth, Germany
*Euphrasia officinalis*	Eyebright	Whole plant	Maros Arznei, Fürth, Germany
*Filipendula ulmaria*	Meadowsweet	Flower	Maros Arznei, Fürth, Germany
*Foeniculum vulgare*	Fennel	Fruit/berry/seed	Maros Arznei, Fürth, Germany
*Fucus vesiculosus*	Bladderwrack	Whole plant	Maros Arznei, Fürth, Germany
*Gentiana lutea*	Gentian	Root	Maros Arznei, Fürth, Germany
*Geranium robertianum*	Herb robert	Whole plant	Maros Arznei, Fürth, Germany
*Ginkgo biloba*	Ginkgo	Leaf	Maros Arznei, Fürth, Germany
*Glycyrrhiza glabra*	Liquorice	Root	Maros Arznei, Fürth, Germany
*Hamamelis virginiana*	Witch hazel	Leaf	Maros Arznei, Fürth, Germany
*Harpagophytum procumbens*	Devil's claw	Root	Maros Arznei, Fürth, Germany
*Hedera helix*	Common ivy	Leaf	Maros Arznei, Fürth, Germany
*Hibiscus sabdariffa*	Roselle	Leaf	Maros Arznei, Fürth, Germany
*Humulus lupulus*	Hops	Flower	Maros Arznei, Fürth, Germany
*Hypericum perforatum*	St John's wort	Whole plant	Maros Arznei, Fürth, Germany
*Ilex paraguariensis*	Yerba mate	Leaf	Maros Arznei, Fürth, Germany
*Juniperus communis*	Common juniper	Fruit/berry/seed	Maros Arznei, Fürth, Germany
*Lavandula angustifolia*	Lavender	Flower	Maros Arznei, Fürth, Germany
*Marrubium vulgare*	Common horehound	Whole plant	Maros Arznei, Fürth, Germany
*Matricaria chamomilla*	Chamomile	Whole plant	Maros Arznei, Fürth, Germany
*Melilotus officinalis*	Sweet clover	Whole plant	Maros Arznei, Fürth, Germany
*Melissa officinalis*	Lemon balm	Leaf	Maros Arznei, Fürth, Germany
*Mentha piperita*	Peppermint	Whole plant	Maros Arznei, Fürth, Germany
*Nicotiana tabacum*	Tobacco	Leaf^1^	British American Tobacco Nederland B.V., Amstelveen, Netherlands
*Origanum majorana*	Marjoram	Whole plant	Maros Arznei, Fürth, Germany
*Panax ginseng*	Ginseng	Root	Maros Arznei, Fürth, Germany
*Petroselinum crispum*	Parsley	Whole plant	Maros Arznei, Fürth, Germany
*Pimpinella anisum*	Anise	Fruit/berry/seed	Maros Arznei, Fürth, Germany
*Plantago lanceolata*	Ribwort	Whole plant	Maros Arznei, Fürth, Germany
*Primula officinalis*	Common cowslip	Root	Maros Arznei, Fürth, Germany
*Primula vulgaris*	Common primrose	Root	Maros Arznei, Fürth, Germany
*Pulmonaria officinalis*	Common lungwort	Flower	Maros Arznei, Fürth, Germany
*Quercus robur*	English oak	Bark^1^	Holger Senger Naturrohstoffe und Gartenbau, Dransfeld, Germany
*Rheum palmatum*	Rhubarb	Root	Maros Arznei, Fürth, Germany
*Rosmarinus officinalis*	Rosemary	Leaf	Maros Arznei, Fürth, Germany
*Rubus fruticosus*	Blackberry	Leaf	Maros Arznei, Fürth, Germany
*Salix alba*	White willow	Bark	Maros Arznei, Fürth, Germany
*Salvia officinalis*	Salvia	Leaf	Maros Arznei, Fürth, Germany
*Sambucus nigra* (L.)	Elderflowers	Flower^1^	Tee und Gewürze Lilianna Kamberg und Marianne Schmidt, Offenbach, Germany
*Schinus terebinthifolius*	Brazilian pepper tree	Fruit/berry/seed^1^	Tee und Gewürze Lilianna Kamberg und Marianne Schmidt, Offenbach, Germany
*Scrophularia nodosa*	Common figwort	Whole plant	Maros Arznei, Fürth, Germany
*Spirulina*	Spirulina	Whole cyanobacteria^1^	VegaVital UG, Berlin, Germany
*Symphytum officinale*	Comfrey	Root	Maros Arznei, Fürth, Germany
*Syzygium aromaticum*	Clove	Flower^1^	FUCHS Gewürze, Dissen, Germany
*Tanacetum parthenium*	Feverfew	Whole plant	Maros Arznei, Fürth, Germany
*Taraxacum officinale*	Dandelion	Whole plant	Maros Arznei, Fürth, Germany
*Thymus vulgaris*	Common thyme	Whole plant	Maros Arznei, Fürth, Germany
*Tropaeolum majus*	Nasturtium	Whole plant	Maros Arznei, Fürth, Germany
*Uncaria tomentosa*	Cat's claw	Whole plant^1^	Herbathek Naturheilmittel, Berlin, Germany
*Urtica dioica*	Stinging nettle	Root	Maros Arznei, Fürth, Germany
*Usnea barbata*	Barber’s itch	Whole plant	Maros Arznei, Fürth, Germany
*Vaccinium myrtillus*	Bilberry	Fruit/berry/seed	Maros Arznei, Fürth, Germany
*Valeriana officinalis* (L.)	Common valerian	Root	Maros Arznei, Fürth, Germany
*Vanilla planifolia*	Vanilla	Fruit/berry/seed	Maros Arznei, Fürth, Germany
*Verbena officinalis*	Common vervain	Whole plant	Maros Arznei, Fürth, Germany
*Vigna radiata*	Mung bean (dried)	Fruit/berry/seed^1^	Thai World Import Export, Bangkok, Thailand
*Vigna radiata*	Mung bean (cooked in boiling water for 20 min)	Fruit/berry/seed^2^	Thai World Import Export, Bangkok, Thailand
*Viscum album*	European mistletoe	Whole plant	Maros Arznei, Fürth, Germany
*Xanthoria parietina*	Common orange lichen	Whole lichen^1^	AG Weber, Max Planck Institute for Chemistry, Mainz, Germany
*Zingiber officinale*	Ginger	Root	Maros Arznei, Fürth, Germany

^1^10 g dry sample used for extract preparation

^2^10 g wet sample used for extract preparation

### Cell cultures and treatments

#### HeLa-TLR4 cell line

The HeLa-TLR4 transfected reporter cell line (Novusbio, Wiesbaden Nordenstadt, Germany) was cultured in Dulbecco's Modified Eagle Medium (DMEM, Thermo Fisher Scientific, Darmstadt, Germany) supplemented with 10% fetal bovine serum (FBS, Biochrom, Berlin, Germany), 1% penicillin-streptomycin (Thermo Fisher Scientific), 5 μg/ml blasticidine (Sigma-Aldrich, Darmstadt, Germany), 1 μg/ml puromycin (Sigma-Aldrich), and 1 mg/ml geneticin (G418, Sigma-Aldrich) at 37°C in humidified atmospheric air supplemented with 5% CO_2_. In black 96-well microplates with clear bottom (Greiner Bio-One, Solingen, Germany), 2×10^5^ cells/ml were seeded in 100 μl medium and allowed to settle overnight. Experiments were conducted with different concentrations of the extracts or the same amount of vehicle (70% ethanol). Concentrations ranging from 0.01% to 3% in cell culture medium were used to receive a dose-response curve and to cover a broad spectrum of concentrations. Due to toxic effects, higher concentrations than 3% were not used in the performed assay systems. Cells were pre-incubated with extracts or vehicle (70% ethanol) for 2 h at 37°C. Afterwards, lipopolysaccharide (LPS-EB, from *E*. *coli* O111:B4, Invivogen, Toulouse, France) in a final concentration of 25 ng/ml was added to stimulate TLR4 (incubation for 8 h at 37°C). Alamar Blue assay was used to determine cell viability and TLR4 stimulation was measured using luciferase assay (Novusbio).

NF-κB translocation experiments were conducted on a monoclonal HeLa-TLR4 cell line (Novusbio) stably transfected with a plasmid expressing constitutive active firefly luciferase, as a reporter for cell viability (HeLa-TLR4 dual reporter cell line). Cells were cultured as described for HeLa-TLR4 reporter cell line, but selective antibiotics were replaced by 140 μg/ml Hygromycin B Gold (Invivogen). HeLa-TLR4 dual reporter cells were seeded on 12-well glass bottom plates (Cellvis, Mountain View, USA) at a concentration of 2.0×10^5^ cells/ml in 1 ml complete medium per well and incubated for 48 h at 37°C. Cells were pre-incubated with 0.6% extracts in final cell culture medium or with the same amount of vehicle control for 2 h at 37°C. After pre-incubation, LPS-EB in a final concentration of 50 ng/ml was added for 1 h at 37°C. Next, cells were washed 1x with phosphate buffered saline (PBS) and fixed with 4% formaldehyde in PBS buffer (Thermo Fisher Scientific) for 10 min at 37°C. Subsequently, cells were washed 3x with PBS before proceeding with immunostaining for NF-κB.

#### THP-1 cell line

The myeloid THP-1 cell line TIB-202 (ATCC; LGC Standards, Wesel, Germany) was cultured in Roswell Park Memorial Institute (RPMI) 1640 medium (Thermo Fisher Scientific) supplemented with 10% FBS (heat-inactivated), 1% penicillin/streptomycin and 0.05 mM β-mercaptoethanol (Sigma-Aldrich) at 37°C in humidified atmospheric air supplemented with 5% CO_2_. In a 96-well microplate (Greiner Bio-One), 4×10^5^ cells/ml were seeded in 80 μl complete medium and were allowed to settle for 1 h. Experiments were conducted at the same extract concentration range used for HeLa-TLR4 cells. Cells were incubated with extracts or vehicle for 2 h at 37°C. Afterwards, LPS-EB in a final concentration of 50 ng/ml was added to stimulate TLR4 (4 h incubation at 37°C). Alamar Blue assay was used to determine cell viability. TLR4 stimulation was measured using enzyme-linked immunosorbent assay (ELISA, BD Biosciences, Heidelberg, Germany).

Polarization experiments were conducted with THP-1 monocytes differentiated to macrophages. In a 96-well microplate (Greiner Bio-One), 1.5x10^5^ cells/ml were seeded in 200 μl complete growth medium containing 10 ng/ml phorbol 12-myristate 13-acetate (PMA, Sigma-Aldrich). Cells were incubated for three days at 37°C to differentiate to M0 macrophages. Afterwards, the medium was exchanged to 200 μl complete growth medium without PMA and cells were incubated overnight at 37°C. Cells were polarized to M1-type macrophages with 20 ng/ml interferon-gamma (IFNγ, Thermo Fisher Scientific) for 24 h. All further steps were performed as described for THP-1 monocytes.

#### HEK-TLR2 and HEK-TLR4 cell lines

The HEK-Blue hTLR2 (HEK-TLR2) and HEK-Blue hTLR4 cell lines (HEK-TLR4) (both Invivogen) were cultured in DMEM high glucose medium supplemented with 10% FBS (heat-inactivated), 1% penicillin/streptomycin and 1x HEK-Blue Selection (Invivogen) at 37°C in humidified atmospheric air supplemented with 5% CO_2_. In experiments examining cell viability, medium without HEK-Blue Selection was used. In experiments analyzing TLR2 and TLR4 stimulation, cells were cultured in HEK-Blue detection medium (Invivogen). Extracts with concentrations between 0.01% and 3% in final cell culture medium or vehicle (70% ethanol) were added in empty 96-well microplates. Directly afterwards, cells were seeded with 2.8×10^5^ cells/ml in 100 μl complete growth medium and were incubated with the extracts for 2 h at 37°C. Subsequently, receptor activity was stimulated by adding 1 ng/ml S-(2,3-bis(palmitoyloxy)-(2RS)-propyl)-(R)-cysteinyl-(S)-seryl-(S)-lysyl-(S)-lysyl-(S)-lysyl-(S)-lysine (Pam2CSK4, HEK-TLR2 cell line, Invivogen) or 100 ng/ml LPS-EB Ultrapure (HEK-TLR4 cell line, LPS from *E*. *coli* O111:B4, Invivogen). Cells were incubated overnight at 37°C. Alamar Blue assay was used to determine cell viability and TLR2/TLR4 stimulation was measured using HEK-Blue detection assay (Invivogen).

### Determination of cell viability

Cell viability was measured by the Alamar Blue assay (Thermo Fisher Scientific) according to the manufacturer’s protocol (10% final concentration of Alamar Blue solution in cell culture medium). Cells were incubated with Alamar Blue solution at 37°C for 4 h (HeLa-TLR4 cell line) or overnight (all other cell lines). Fluorescence intensity was measured with microplate reader Synergy Neo (Biotek, Bad Friedrichshall, Germany) at an excitation wavelength of 560 nm and an emission wavelength of 590 nm.

### IL-8 transcriptional activity in HeLa-TLR4 cells

LightSwitch Luciferase assay (Novusbio) was used to determine production of renilla luciferase under transcriptional control of interleukin-8 (IL-8) promoter. HeLa-TLR4 cells were washed with PBS (Thermo Fisher Scientific) after performing the cell viability assay to remove the Alamar Blue staining and were frozen overnight at -80°C for better cell lysis. After thawing the cells, LightSwitch Luciferase assay was performed according to manufacturer’s protocol and luminescence was measured with microplate reader Synergy Neo (Biotek).

### IL-8, IL-10 and TNF-α secretion

Supernatants of pre-treated THP-1 monocytes and macrophages were examined for concentration of IL-8, IL-10 or tumor necrosis factor alpha (TNF-α) using ELISAs (BD Biosciences). Sandwich ELISA was carried out according to manufacturer’s protocol with optimized washing buffer volume. Supernatant was diluted up to 6-fold (total used volume: 100 μl/well). Absorbance was measured using a Synergy Neo plate reader at a wavelength of 450 nm and a reference wavelength of 570 nm. Based on pipetted standard values, a four-parameter logarithmic standard curve was determined with the Gen5 software on SynergyNeo (Biotek). Cytokine production of THP-1 monocytes and macrophages were calculated according to standard curves and dilution factors.

### Determination of transcription activity in HEK-Blue cells

HEK-Blue Detection assay (Invivogen) was used to determine production of inducible secreted embryonic alkaline phosphatase (SEAP), based on activation of NF-κB and activator protein 1 (AP-1). HEK-Blue cells were cultured in HEK-Blue Detection medium during incubation with extracts and stimulation of receptor activity. Afterwards, stimulation of TLR2 (HEK-TLR2 cell line) and TLR4 (HEK-TLR4 cell line) were determined using a Synergy Neo plate reader (Biotek) at a wavelength of 640 nm.

### Determination of NF-κB p65 translocation by fluorescence microscopy

For immunostaining of NF-κB, HeLa-TLR4 dual reporter cells were incubated in blocking solution consisting of 5% bovine serum albumin (BSA, Cell Signaling Technology, Cambridge, UK) and 0.3% Triton X-100 (Merck, Darmstadt, Germany) in PBS for 1 h at RT. Next, cells were incubated with primary anti-NF-κB p65 antibody (D14E12, Cell Signaling Technology) overnight at 4°C. After washing, secondary anti-rabbit Alexa Fluor 568 antibody was applied (Thermo Fisher Scientific) for 1 h at RT. Both antibodies were diluted 1:400 in PBS containing 1% BSA and 0.3% Triton X-100. Cell nuclei were counterstained using 4’,6-diamidino-2-phenylindole (DAPI, Thermo Fisher Scientific).

Stained cells were imaged using the Opera Phenix High-Content Screening System (PerkinElmer, Waltham, USA) with laser lines for Alexa Fluor 568 at 568 nm excitation and 570 nm—630 nm emission and laser lines for DAPI at 405 nm excitation and 435 nm—480 nm emission. Image processing was done using Harmony software (PerkinElmer). Nuclear masks (DAPI stained cell nuclei) and surrounding ring-like masks (cytoplasm) were identified and the mean fluorescence intensity ratio of nuclear to cytoplasmic NF-κB was determined.

### Statistical analyses

Statistical analyses were accomplished in GraphPad Prism 5.01 (GraphPad Software, San Diego, California, USA, www.graphpad.com) conducting one-way ANOVA followed by Dunnett’s post hoc test. Values of p<0.05 were considered as significant. For statistical analyses of data from macrophage polarization, ANOVA was accomplished, followed by an unpaired t-test. Values of p<0.05 were considered significant.

## Results

### Screening of ethanolic herbal extracts for TLR-dependent anti-inflammatory effects

**Fig 1:**
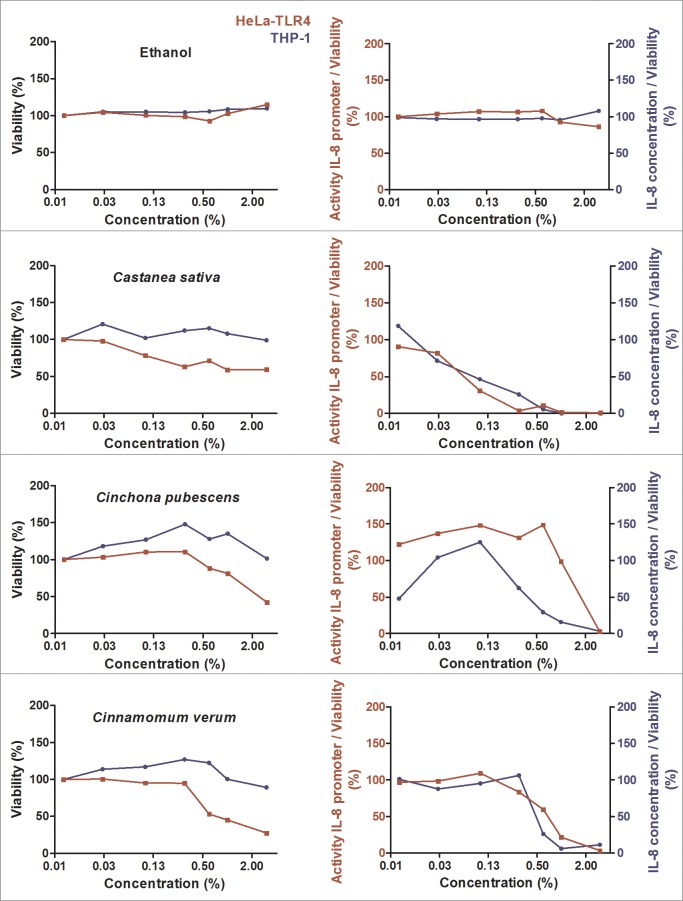
Cell viability and concentration-dependent anti-inflammatory effects of selected herbal extracts (part 1). HeLa-TLR4 cells (red) and THP-1 monocytes (blue) were incubated with extracts in different concentrations or vehicle (70% ethanol), followed by stimulation with LPS-EB. Viability was measured using the Alamar Blue Assay and was normalized to the negative control (untreated cells). TLR4 receptor stimulation was measured using Renilla luciferase expression for the HeLa-TLR4 cell line and IL-8 ELISA (pg/ml) for the THP-1 monocytes and was normalized to ethanol-treated cells. Data are displayed as viability (%) in the left graphs and TLR4 stimulation divided by normalized viability (%) in the right graphs. Data represents means (*n*≥2). For graphical display of further extracts, see Fig 2, Fig 3 and supplementary data S1 Fig.

**Fig 2:**
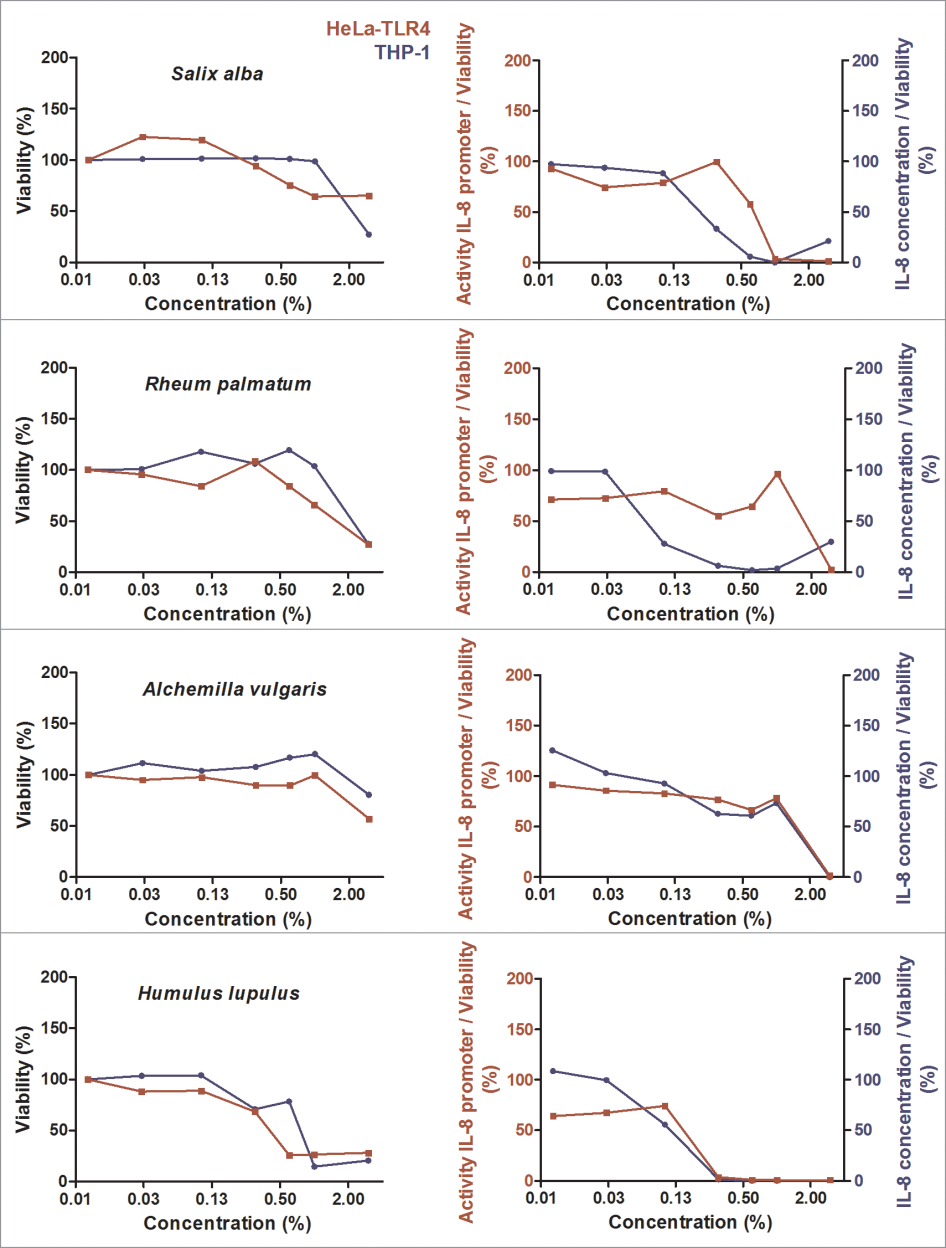
Cell viability and concentration-dependent anti-inflammatory effects of selected herbal extracts (part 2). HeLa-TLR4 cells (red) and THP-1 monocytes (blue) were incubated with extracts in different concentrations or vehicle (70% ethanol), followed by stimulation with LPS-EB. Viability was measured using the Alamar Blue Assay and was normalized to the negative control (untreated cells). TLR4 receptor stimulation was measured using Renilla luciferase expression for the HeLa-TLR4 cell line and IL-8 ELISA (pg/ml) for the THP-1 monocytes and was normalized to ethanol-treated cells. Data are displayed as viability (%) in the left graphs and TLR4 stimulation divided by normalized viability (%) in the right graphs. Data represents means (*n*≥2). For graphical display of further extracts, see Fig 1, Fig 3 and supplementary data S1 Fig.

**Fig 3:**
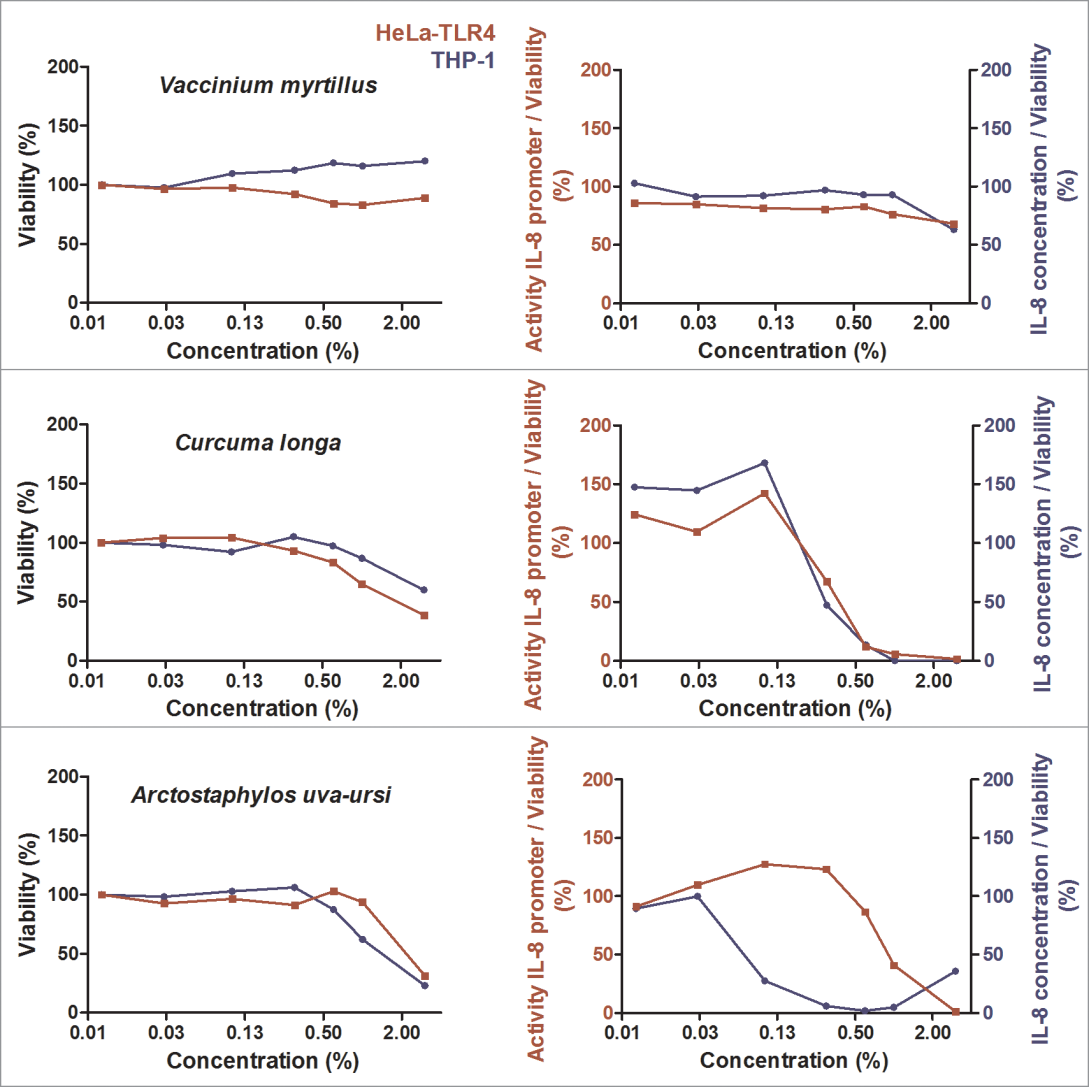
Cell viability and concentration-dependent anti-inflammatory effects of selected herbal extracts (part 3). HeLa-TLR4 cells (red) and THP-1 monocytes (blue) were incubated with extracts in different concentrations or vehicle (70% ethanol), followed by stimulation with LPS-EB. Viability was measured using the Alamar Blue Assay and was normalized to the negative control (untreated cells). TLR4 receptor stimulation was measured using Renilla luciferase expression for the HeLa-TLR4 cell line and IL-8 ELISA (pg/ml) for the THP-1 monocytes and was normalized to ethanol-treated cells. Data are displayed as viability (%) in the left graphs and TLR4 stimulation divided by normalized viability (%) in the right graphs. Data represents means (*n*≥2). For graphical display of further extracts, see Fig 1, Fig 2 and supplementary data S1 Fig.

**Fig 4:**
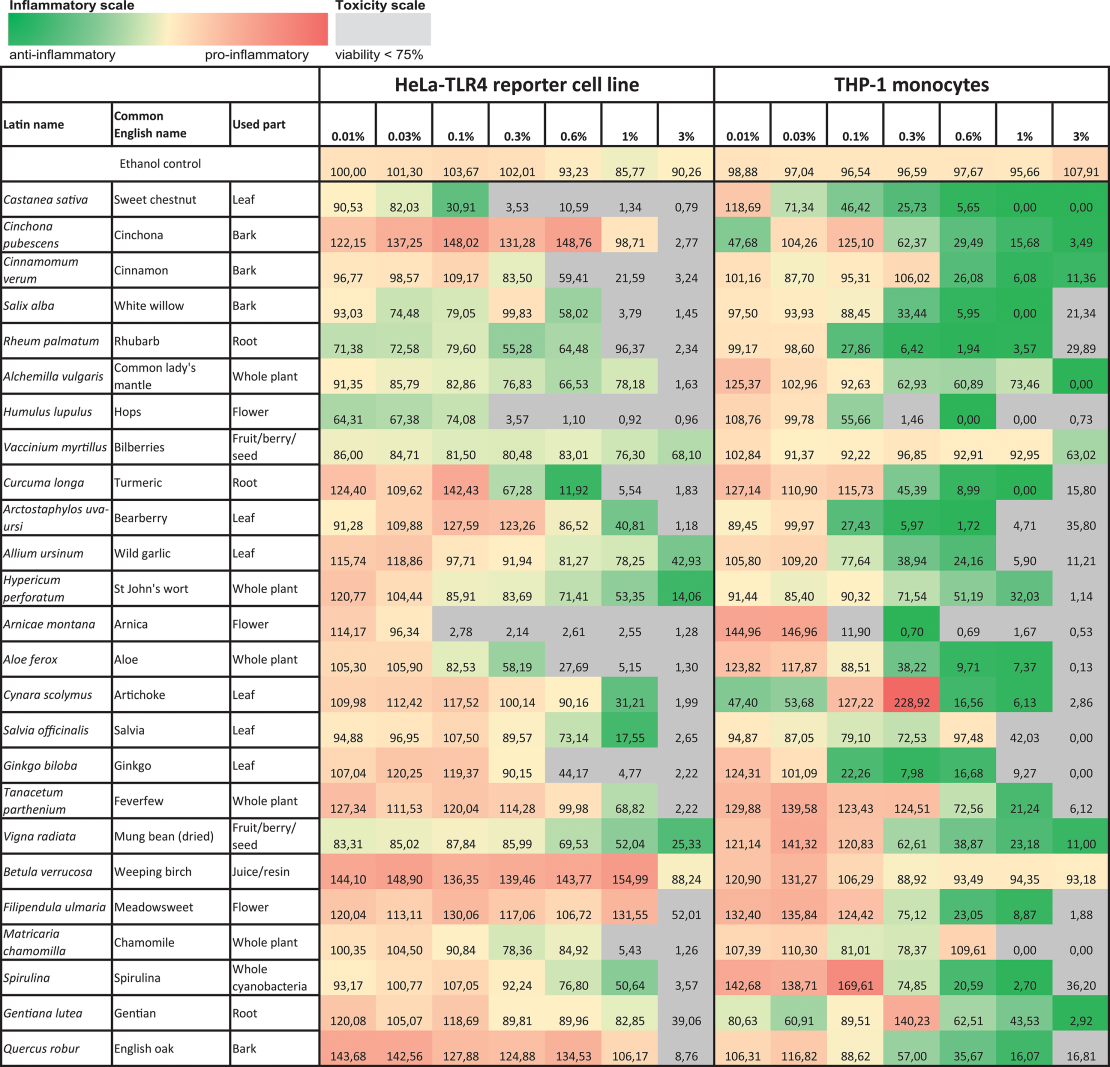
Ethanolic extracts with highest anti-inflammatory activity. HeLa-TLR4 cells or THP-1 monocytes were incubated with extracts in different concentrations or vehicle (70% ethanol), followed by stimulation with LPS-EB. Viability was measured using the Alamar Blue Assay and was normalized to the negative control (untreated cells) (*Viability (%)*). TLR4 receptor activity was measured using Renilla luciferase expression for the HeLa-TLR4 cell line or IL-8 ELISA for the THP-1 monocytes and was normalized to ethanol-treated cells (*TLR4-Activity*). Data are displayed as TLR4 stimulation divided by viability and ranked ascending by the following formula: (150—*Viability (%)*) * (2 * *TLR4-Activity* + 100) weighted in a ratio of 2:1 for THP-1 monocytes vs. HeLa-TLR4 cells. The 25 extracts with the highest mitigation of LPS-induced inflammatory signal are displayed here (for comparison of all extracts see S2 Fig). Data represents means (*n*≥2).

During initial screening, 99 ethanolic extracts (96 herbal extracts, one cyanobacterial, one green algae and one lichen extract) were tested for their anti-inflammatory activity. Ten extracts displayed major anti-inflammatory activity combined with a high cell viability (Fig 1, Fig 2 and Fig 3). The results for the other 89 extracts are graphically displayed in the supplementary data (S1 Fig). Comparing the effects in both cell lines used for efficacy and toxicity readout, HeLa-TLR4 transfected reporter cells were more susceptible to toxic effects of most extracts. THP-1 monocytes showed a viability above 85% after treatment with the ten most effective anti-inflammatory extracts in concentrations up to 1%, except for *Humulus lupulus* and *Arctostaphylos uva-ursi* (Fig 1, Fig 2 and Fig 3). Some extracts such as *Castanea sativa*, *Cinnamomum verum*, *Humulus lupulus* and *Curcuma longa* revealed comparable anti-inflammatory efficacy in both cell lines, whereas other extracts (e.g. *Cinchona pubescens* and *Rheum palmatum*) were more effective in THP-1 monocytes than in HeLa-TLR4 cells. *Castanea sativa* was the only extract with a dose-dependent anti-inflammatory activity already at low concentrations, whereas most of the other extracts possessed an inhibition threshold (e.g. between 0.3% and 0.6% for cinnamon extract). Ethanolic extracts with the highest anti-inflammatory potential were sweet chestnut (*Castanea sativa*), cinchona (*Cinchona pubescens*), cinnamon (*Cinnamomum verum*), white willow (*Salix alba*), rhubarb (*Rheum palmatum*), common lady’s mantle (*Alchemilla vulgaris*), hops (*Humulus lupulus*), bilberries (*Vaccinium myrtillus*), turmeric (*Curcuma longa*) and bearberry (*Arctostaphylos uva-ursi*) (Fig 1, Fig 2 and Fig 3). Three bark extracts were within the top five anti-inflammatory extracts (number 2: *Cinchona pubescens*, number 3: *Cinnamomum verum* and number 4: *Salix alba*). Fig 4 shows the 25 most effective anti-inflammatory extracts and S2 Fig displays all 99 extracts with a ranking according to their anti-inflammatory potential. For visualization, heat maps display TLR4 stimulation normalized to viability shown in green for anti-inflammatory and in red for pro-inflammatory effects. THP-1 monocytes naturally express TLR4 and several other innate immune receptors, in contrast to HeLa-TLR4 cells, which were transfected with TLR4. THP-1 monocytes more reflect the physiological mechanisms in the human body, therefore effects on THP-1 monocytes vs. HeLa-TLR4 cells were weighted in a ratio of 2:1 for ranking of the extracts. In addition, TLR4 inhibition was calculated with a higher impact than toxic effects. Extracts resulting in anti-inflammatory activity but with a viability below 75% should be viewed critically, since the anti-inflammatory effects have to be rather attributed to toxicity. In the heat maps these values are displayed in grey.

### Screening for exclusive TLR4 antagonistic effects

**Fig 5:**
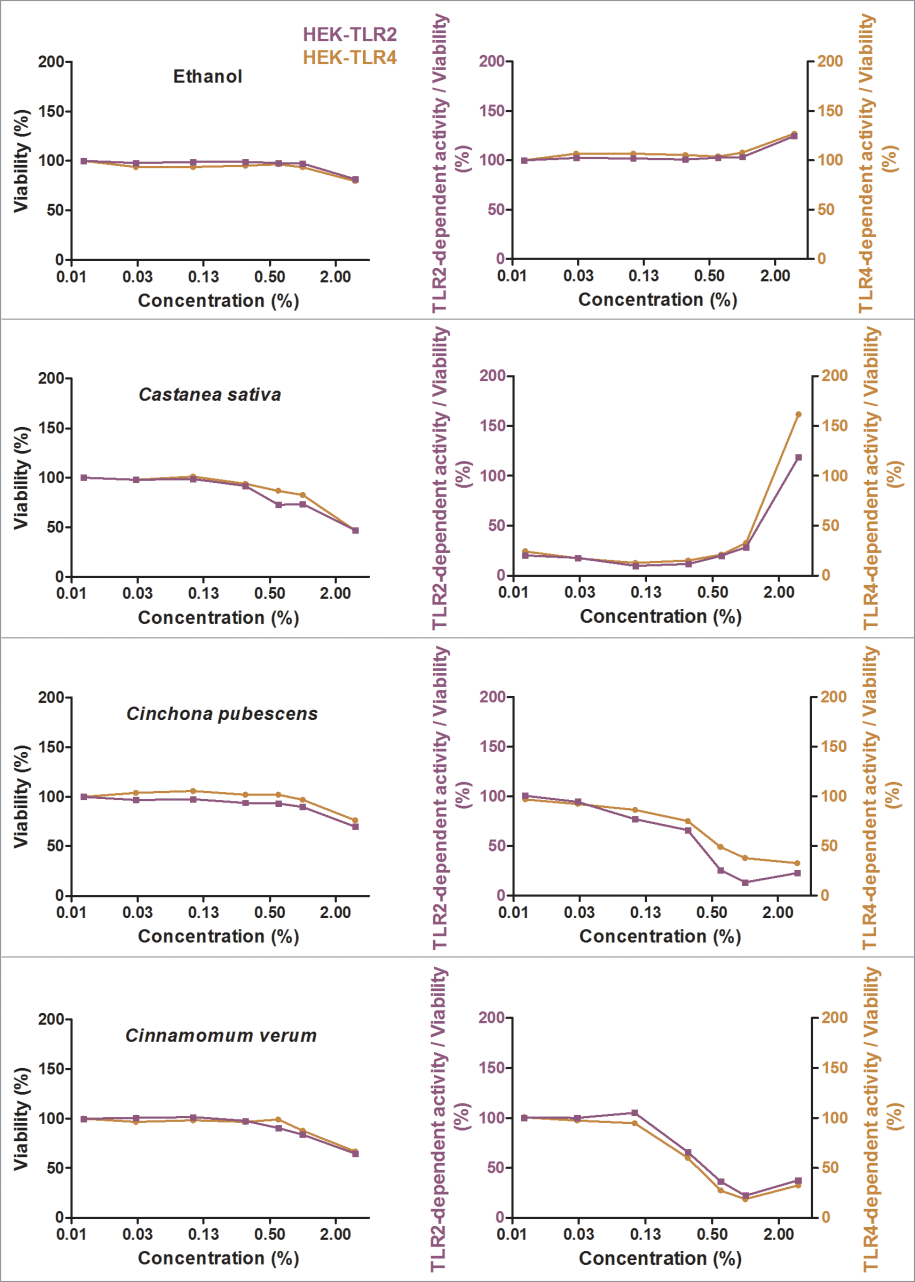
Extracts with TLR2 and TLR4 antagonistic effects (part 1). HEK-TLR2 cells (purple) and HEK-TLR4 cells (orange) were incubated with extracts in different concentrations or vehicle (70% ethanol), followed by stimulation of HEK-TLR2 cells with Pam2CSK4 or HEK-TLR4 cells with LPS-EB Ultrapure. Viability was measured using the Alamar Blue Assay and was normalized to the negative control (untreated cells). TLR2 and TLR4 receptor stimulation were measured using SEAP production and were normalized to ethanol-treated cells. Data are displayed as viability (%) in the left graphs and TLR4 stimulation divided by viability (%) in the right graphs. Data represents means (*n*≥4). For graphical display of further extracts, see Fig 6 and supplementary data S3 Fig.

**Fig 6:**
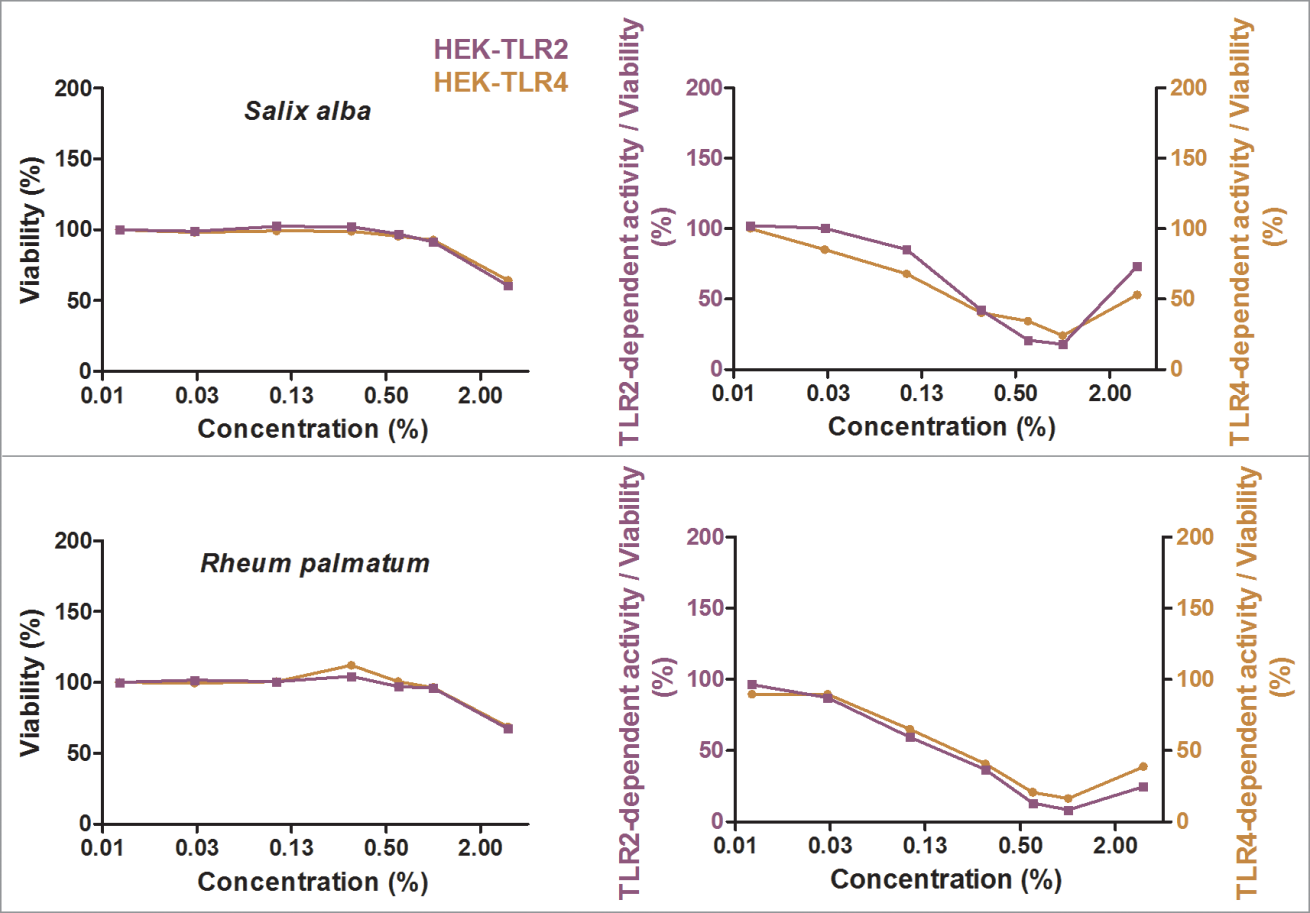
Extracts with TLR2 and TLR4 antagonistic effects (part 2). HEK-TLR2 cells (purple) and HEK-TLR4 cells (orange) were incubated with extracts in different concentrations or vehicle (70% ethanol), followed by stimulation of HEK-TLR2 cells with Pam2CSK4 or HEK-TLR4 cells with LPS-EB Ultrapure. Viability was measured using the Alamar Blue Assay and was normalized to the negative control (untreated cells). TLR2 and TLR4 receptor stimulation were measured using SEAP production and were normalized to ethanol-treated cells. Data are displayed as viability (%) in the left graphs and TLR4 stimulation divided by viability (%) in the right graphs. Data represents means (n≥4). For graphical display of further extracts, see Fig 5 and supplementary data S3 Fig.

**Fig 7:**
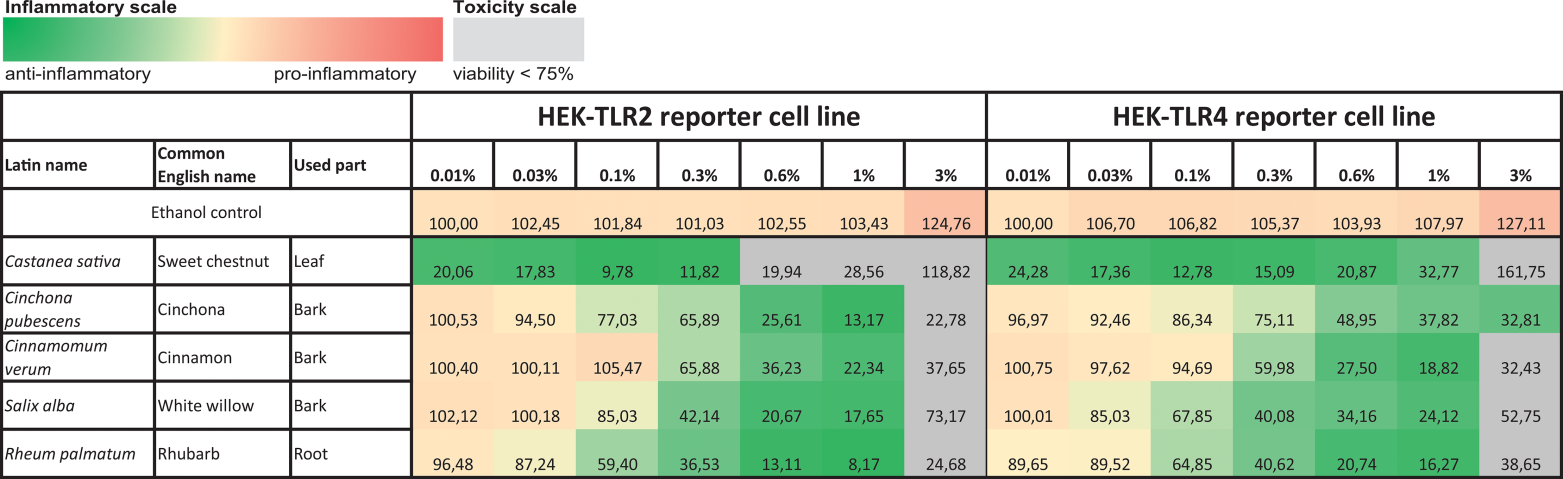
Ethanolic extracts with TLR2 and TLR4 antagonistic activities in HEK-TLR2 and HEK-TLR4 cell lines. HEK-TLR2 or HEK-TLR4 cells were incubated with extracts in different concentrations or vehicle (70% ethanol), followed by stimulation of HEK-TLR2 cells with Pam2CSK4 or HEK-TLR4 cells with LPS-EB Ultrapure. Viability was measured using the Alamar Blue Assay and was normalized to the negative control (untreated cells). TLR2 and TLR4 receptor activity were measured using SEAP production and were normalized to ethanol-treated cells. Data are displayed as receptor stimulation divided by normalized viability. The five extracts with the highest mitigation of LPS-induced inflammatory signal from Fig 4 are displayed here (for comparison of further extracts, see S4 Fig). Data represents means (*n*≥4).

Besides the identification of anti-inflammatory extracts, we were also interested in specific antagonists for TLR4. Twenty-eight ethanolic extracts with strong anti-inflammatory effects were additionally tested in a comparative assay with Pam2CSK4-stimulated HEK-TLR2 and LPS-EB Ultrapure-stimulated HEK-TLR4 cells to discriminate a direct TLR4 antagonistic effect from interference with downstream signaling pathways shared by TLR2 and TLR4 signaling. All tested extracts exhibited mitigation of TLR2- and TLR4-dependent responses (see Figs 5 and 6 for the five most effective anti-inflammatory extracts and S3 Fig for all other extracts). For most of the extracts, especially the most effective anti-inflammatory extracts, HEK-TLR2 and HEK-TLR4 cell lines showed comparable dose-dependent anti-inflammatory effects. Interestingly, *Castanea sativa* leaves inhibited TLR2- and TLR4-dependent inflammatory responses already at 0.01% extract dilution, accompanied by a cell viability of 98%. An increase of inflammatory activity was observed for most of the extracts with concentrations between 1% and 3% in cell culture medium. This may be due to toxic effects and should therefore be viewed critically. Heat maps displaying the TLR2- and TLR4 stimulation are shown in Fig 7 (the five most promising extracts) and S4 Fig (all 28 extracts), with anti-inflammatory activity shown in green to pro-inflammatory activity shown in red. The ranking of the extracts was taken from the THP-1 and HeLa-TLR4 screening shown in Fig 4 and S2 Fig. Values with viability below 75% are marked in grey.

### Inhibition of NF-κB p65 translocation by select extracts

**Fig 8:**
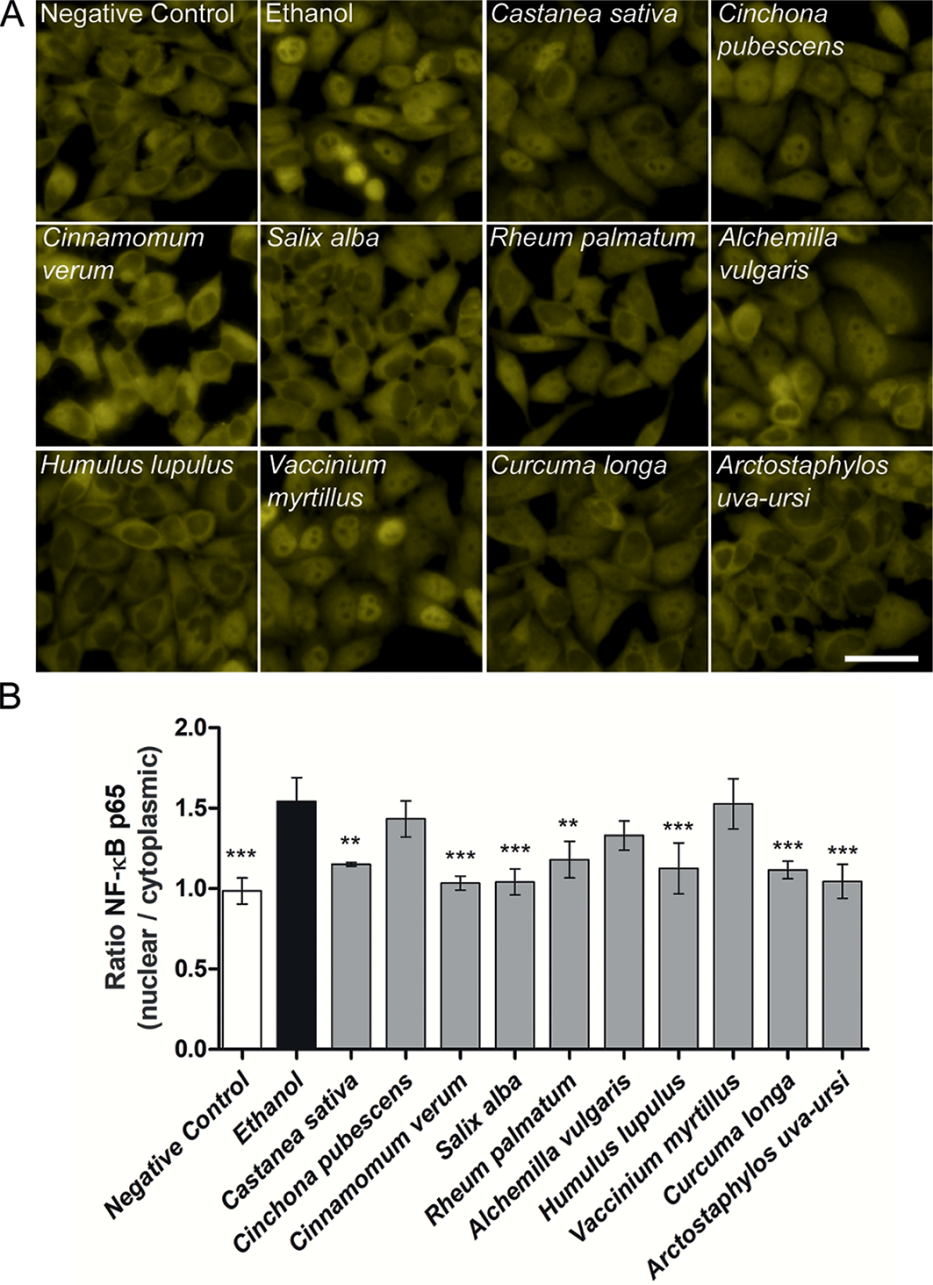
NF-κB translocation of select anti-inflammatory extracts. **A**: Fluorescence microscopy images of NF-κB stained HeLa-TLR4 dual reporter cells incubated with extracts or vehicle (70% ethanol), followed by stimulation with LPS-EB. For better visibility, images were cropped and adjusted in brightness and contrast. Scale bar = 50 μm. **B**: Quantitative evaluation of NF-κB p65 translocation. Mean fluorescence ratios of nuclear to cytoplasmic NF-κB p65 were calculated and compared to ethanol control. Data represents means ± SD (*n* = 3, 72 images (fields) per experiment and per treatment condition). Dunnett’s post hoc test with **p<0.01; ***p<0.001 compared to ethanol control.

Several pro-inflammatory signaling pathways cumulate in the nuclear translocation of the transcription factor NF-κB. Therefore, the ten extracts with the highest anti-inflammatory activity, combined with no or low toxicity in THP-1 and HeLa-TLR4 cells (Fig 1, Fig 2 and Fig 3) were tested regarding their influence on NF-κB p65 translocation in LPS-stimulated HeLa-TLR4 dual reporter cells. Here *Castanea sativa*, *Cinnamomum verum*, *Salix alba*, *Rheum palmatum*, *Humulus lupulus*, *Curcuma longa* as well as *Arctostaphylos uva-ursi* significantly inhibited NF-κB translocation, compared to the LPS-stimulated vehicle control (Fig 8). Interestingly, *Cinchona pubescens*, *Alchemilla vulgaris* and *Vaccinium myrtillus*, which demonstrated strong anti-inflammatory activity in the THP-1 and HeLa-TLR4 as well as in the comparative HEK-TLR2/HEK-TLR4 assays, did not prevent the LPS-induced NF-κB translocation into the nucleus, indicating interference with another, alternative pro-inflammatory mechanism.

### Repolarization of THP-1 macrophages by select extracts

**Fig 9:**
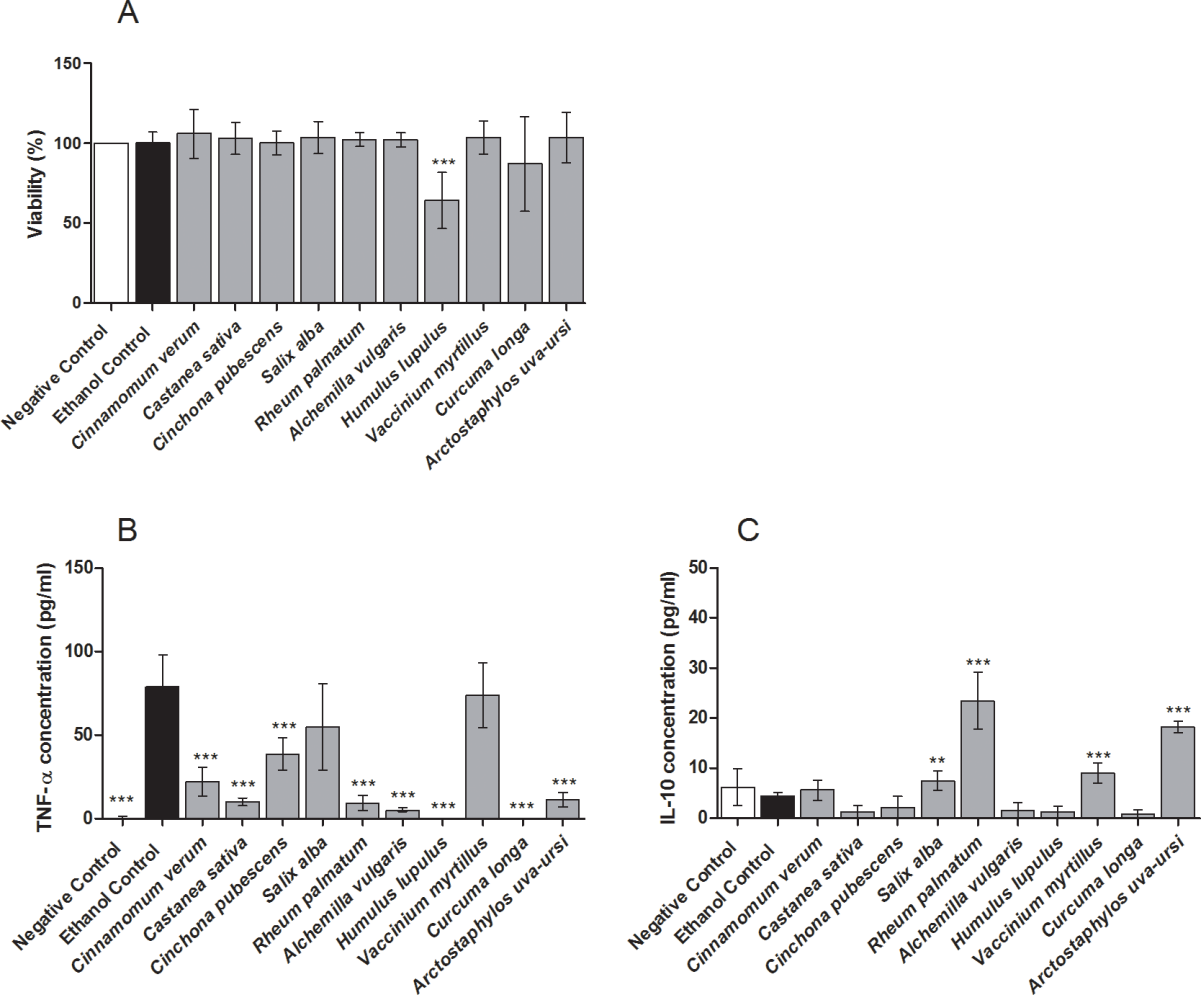
Effect of ten most effective anti-inflammatory extracts on macrophage repolarization. THP-1 M1 macrophages were incubated with extracts or vehicle (70% ethanol) control, followed by stimulation with LPS-EB. Negative control: untreated M1-type macrophages. **A**: Viability (Alamar Blue assay) was normalized to viability of untreated cells. **B**: TNF-α secretion (pg/ml) measured by ELISA. **C**: IL-10 secretion (pg/ml) measured by ELISA. Data represents means ± SD of 2 independent experiments (each with *n* = 3); unpaired t-test with ***p<0.001, **p<0.005 compared to respective ethanol control.

The same ten extracts were additionally tested for their influence on repolarization of pro-inflammatory M1-type to anti-inflammatory M2-type macrophages. Here, enhanced secretion of TNF-α is an indicator for M1-type polarization, whereas IL-10 secretion suggests M2-type polarization. All tested M1-polarized, LPS and extract treated THP-1 macrophages showed a viability above 85%, except for *Humulus lupulus* extract, which resulted in 64% viability compared to the vehicle control (Fig 9A). A significant mitigation of TNF-α secretion was observed after incubation with all extracts, except for *Salix alba* and *Vaccinium myrtillus* (Fig 9B). Furthermore, IL-10 production was significantly induced by several extracts, especially *Rheum palmatum* and *Arctostaphylos uva-ursi* (Fig 9C).

## Discussion

Potent anti-inflammatory effects were observed in THP-1 monocytes and HeLa-TLR4 cells after treatment with various ethanolic extracts (Fig 1, Fig 2 and Fig 3). In addition, the most effective extracts were tested in a comparative assay of HEK-TLR2 and HEK-TLR4 cells (Figs 5 and 6). Comparable dose-dependent anti-inflammatory activities in both transfected HEK cell lines suggested that all tested extracts interfere with the NF-κB/AP-1 signaling pathways of TLR2 and TLR4 (and some other innate immune receptors). Thus, it is reasonable that no exclusive TLR4 antagonist was found among the tested extracts. Furthermore, the ten most effective extracts were tested for their ability to inhibit translocation of NF-κB p65 into the cell nucleus (Fig 8). Seven extracts mitigated NF-κB p65 translocation. Despite being anti-inflammatory in THP-1 and HeLa-TLR4 screening as well as in the comparative HEK-TLR2/HEK-TLR4 assay, three extracts (*Cinchona pubescens*, *Alchemilla vulgaris* and *Vaccinium myrtillus*) showed no effect on NF-κB p65 translocation. This is remarkable, since nuclear translocation of NF-κB is an essential step in inflammation. Thus, other signaling pathways, such as those triggered by TNF-α receptor (TNFαR) and interleukin-1 receptor (IL-1R) lead to the observed translocation of NF-κB, however, without IL-8 cytokine release. Those three extracts that lack inhibition of NF-κB translocation may inhibit other molecules downstream of NF-κB. Ethanolic and organic extracts were shown to mainly comprise of anti-inflammatory compounds, whereas water extracts primarily comprise of compounds stimulating the immune system [23, 24]. A mixture of anti-inflammatory and pro-inflammatory compounds within our tested 70% ethanolic extracts might therefore serve as a partial explanation for the contradictory results between the anti-inflammatory effects in HeLa-TLR4 and THP-1 screening vs. the lack of inhibition of LPS-induced NF-κB translocation observed after incubation with *Cinchona pubescens*, *Alchemilla vulgaris* and *Vaccinium myrtillus*. Furthermore, incubation with two out of the ten most effective extracts (*Rheum palmatum* and *Arctostaphylos uva-ursi*, Fig 9) suggest an activity to repolarize pro-inflammatory and tissue destructive M1-type towards M2-type macrophages, which are involved in tissue repair and angiogenesis [25]. Comparing both cell lines, THP-1 monocytes were more susceptible to TLR4-dependent anti-inflammatory effects than HeLa-TLR4 cells. Since HeLa-TLR4 cells were co-transfected with the functional human TLR4, MD-2 and CD-14 complex they do exhibit high TLR4 canonical pathway specificity compared to other TLR4-related pathways. Extracts with stronger anti-inflammatory effects in THP-1 monocytes could possibly influence molecular mechanisms absent in HeLa-TLR4 cells. Therefore, the results observed in THP-1 monocytes may be better suited to reflect physiological TLR4 signaling.

In the NF-κB translocation experiments, the incubation time differs from those in THP-1 and HeLa-TLR4 based screening assays (1 h vs. 4–8 h incubation with LPS), which might also explain the differing results. These LPS incubation times were chosen, as influences of the extracts on THP-1 and HeLa TLR4 screening were best seen after 4 h and 8 h, whereas NF-κB showed highest translocation into the nucleus at 1-2 h.

Due to the high number of extracts tested, we focused on the ten most effective anti-inflammatory extracts identified on screening. These included *Castanea sativa* leaves, *Cinchona pubescens* bark, *Cinnamomum verum* bark, *Salix alba* bark, *Rheum palmatum* root, *Alchemilla vulgaris* plant, *Humulus lupulus* cones, *Vaccinium myrtillus* berries, *Curcuma longa* root and *Arctostaphylos uva-ursi* leaves. To the best of our knowledge, *Castanea sativa* leaves and *Alchemilla vulgaris* plant extracts had not been reported to modulate the NF-κB, TLR2 or TLR4 signaling pathways, thus representing new promising candidates for further investigations. However, spiny burrs of sweet chestnuts inhibited NF-κB activation *in vivo* [26] and ellagitannins present in *Alchemilla vulgaris* were shown to possess NF-κB dependent anti-inflammatory effects [27].

### Sweet chestnut (*Castanea sativa*)—leaves

Strong dose-dependent anti-inflammatory effects, represented by decreased IL-8 concentrations, were observed after sweet chestnut extract incubation. In addition, high anti-inflammatory activity in the comparative HEK-Blue assay, even at low extract concentrations, were found. Apparently, sweet chestnut leaf extracts affect the signaling pathway of both TLR2 and TLR4. Sweet chestnut extract is used in traditional medicines for treatment of skin and soft tissue infections [28]. In modern studies several medicinal properties have been reported, e.g. anti-oxidant and anti-microbial effects [28–30]. Spiny burrs of sweet chestnuts applied for hepatorenal injury in diabetic rats inhibit NF-κB activation [27]. The carotenoid content in fruit and leaves has been suggested to possess direct anti-oxidant effects as well as to influence redox-sensitive and vitamin A signaling pathways [29,31]. Nevertheless, for sweet chestnut leaves only limited information has been available concerning its anti-inflammatory potential, including its ability to suppress TLR2 or TLR4 signaling pathways.

### Common lady’s mantle (*Alchemilla vulgaris)–*whole plant

In our experiments, common lady’s mantle plant extract led to a decrease of LPS-induced IL-8 release and showed inhibitory effects on stimulated signaling pathways of both TLR2 and TLR4, but it displayed no inhibitory effects on nuclear NF-κB translocation. Thus, these results offer a promising starting point for identification of novel pro-inflammatory pathways and anti-inflammatory lead compounds. Common lady’s mantle has been used traditionally in Europe to treat several disorders like urogenital diseases, eczema, inflammation, diarrhea and sepsis [32, 33]. But also, in modern science, beneficial health effects like anti-viral, anti-oxidant and wound-healing properties have been reported [32–35]. Again, its properties to mitigate TLR2 or TLR4 stimulation and to influence NF-κB signaling has not yet been described. However, ellagitannins, which are known to be present in *Alchemilla vulgaris*, were reported to mitigate inflammatory effects via the NF-κB pathway [27].

Besides these two extracts, with newly TLR2 and TLR4 dependent anti-inflammatory effects, the anti-inflammatory activities of our remaining herbal extracts are supported by existing descriptions in the literature. For several of these extracts, anti-inflammatory effects or the influence on TLR2 and TLR4 signaling pathways were solely described for single compounds but not for the complex mixtures present within the whole extract. Therefore, it is possible that further compounds, not investigated so far, contribute to their beneficial health effects.

### Bearberry (*Arctostaphylos uva-ursi*)–leaves

In our assays, bearberry leaves showed strong dose-dependent anti-inflammatory activities in several cell lines and inhibition of LPS-induced NF-κB translocation. In addition, this extract mitigated the secretion of TNF-α accompanied with an increased secretion of IL-10 in macrophages, indicating a switch from pro-inflammatory M1-type to anti-inflammatory M2-type macrophages [26]. Bearberry leaves are traditionally used to treat symptoms of lower urinary tract infections and have been proven in modern medicine to possess anti-septic and anti-adhesion properties [36]. The most representative constituent of bearberry, the phenolic glycoside arbutin, is an anti-oxidant and anti-inflammatory agent [37]. Among others, arbutin significantly reduced production of pro-inflammatory cytokines including IL-1β and TNF-α, and other inflammation-related genes such as monocyte chemoattractant protein-1 (MCP-1) and IL-6. Furthermore, it inhibits the nuclear translocation of NF-κB [37]. These data support our data. Although arbutin is the major active ingredient, whole bearberry extracts are necessary to reveal the complete pharmaceutical activity [38]. The complex mixture of bearberry extract has not yet been reported to influence TLR2/TLR4 signaling pathways or to induce macrophage repolarization, making it a suitable candidate for further investigations.

### Cinchona (*Cinchona pubescens)–*bark

Cinchona bark extract diminished LPS-induced inflammatory signals, especially in THP-1 monocytes and the comparative HEK-TLR2/TLR4 assay system, whereas LPS-induced NF-κB translocation was not affected. *In vivo* inhibition of TLR2- and TLR4-mediated signaling cascades by cinchonine from cinchona bark are demonstrated in literature, supporting our observed inhibition of pro-inflammatory cytokine production in stimulated TLR2 and TLR4 signaling pathways [39]. Besides cinchonine, further quinine derivatives in cinchona bark, e.g. chloroquine inhibited different TLR signaling pathways [40,41]. However, whole cinchona bark extract has, based on our present knowledge, not yet been described to influence TLR2 and/or TLR4 signaling pathways. Further analyses need to be performed to explore the underlying active constituents, besides cinchonine, especially those that affect TLR2 and TLR4 signaling.

### Hops (*Humulus lupulus)–*cones

A strong decrease of pro-inflammatory cytokine production and a mitigation of LPS-induced NF-κB translocation were observed after hops extract treatment in our cell culture-based assays. Flavonoids from hops cones possess pharmaceutically important properties, e.g. anti-oxidant, anti-microbial, anti-carcinogenic, anti-inflammatory and estrogenic effects [42]. In particular, flavonoids like xanthohumol are effective inhibitors of arachidonic acid metabolism through inhibition of cyclooxygenase 1 and 2 (COX-1, COX-2) [43]. Furthermore, LPS-induced production of nitric oxide (NO), IL-1β, and TNF-α, and the activation of NF-κB signaling were inhibited by xanthohumol isolated from hops [44,45]. Again, it is likely that further compounds present in hops extract contribute to its strong anti-inflammatory effects demonstrated in our experiments.

### White willow (*Salix alba)–*bark

Strong dose-dependent anti-inflammatory effects, as shown by decreased IL-8 production, were observed in TLR2- and TLR4-stimulated cell lines, coupled with a significant decrease in LPS-induced NF-κB translocation. White willow extract is commonly known for its beneficial health effects. Its active compound, the alcoholic β-glucoside salicin, was the basis for the discovery of Aspirin, a COX inhibitor, which still is one of the most widely used medicines in the world [46]. During absorption, salicin is metabolized into several salicylate derivatives resulting in pharmaceutical activities based on various components. This leads to a different mode of action compared to Aspirin and its pure compound acetylsalicylic acid, with less severe side effects [47]. In numerous *in vitro* and *in vivo* studies, anti-inflammatory effects have been shown for white willow extract, e.g. downregulation of several pro-inflammatory cytokines like TNF-α and inhibition of NF-κB translocation, which supports the data obtained by us [48–50]. The anti-inflammatory effects have been credited to salicin [48]. However, salicin alone cannot satisfactorily explain the anti-inflammatory effects, and other compounds within the complex mixture, especially polyphenols such as flavonoids and proanthocyanidins, are suggested to contribute to its overall activity and thereby broaden the mechanisms of action [51].

### Turmeric (*Curcuma longa*)–root

Our results suggest that TLR2/TLR4 signaling pathways are molecular targets of turmeric extract. This is supported by reports that show that turmeric and some of its compounds, especially curcumin and aromatic-turmerone, inhibit LPS-induced NF-κB activation, and expression of TLR4 and the downstream genes IL-1 receptor-associated kinase 1 (IRAK-1) and tumor necrosis factor receptor-associated factor 6 (TRAF-6) [52]. Treatment with curcumin inhibits both MyD88- and TRAM/TRIF-dependent pathways in LPS-induced TLR4 signaling. Production of TNF-α, IL-6 and ROS as well as levels of TLR4, MyD88 and the downstream effectors including NF-κB, interferon regulatory factor 3 (IRF3), MyD88, and TIRF were attenuated [53–55]. Incubation with aromatic-turmerone, another compound of turmeric extract, blocked TLR4-mediated downstream signaling and the release of pro-inflammatory mediators [56]. Furthermore, *Curcuma longa* induced a shift from M1-type to M2-type macrophages in the murine RAW 264.7 macrophage cell line [57], which is in line with our data showing a decreased TNF-α secretion.

### Rhubarb (*Rheum palmatum)–*root

Rhubarb root extract led to a decrease of LPS-induced cytokine production, inhibited both TLR2 and TLR4 signaling, nuclear NF-κB translocation, and induced M1-type to M2-type macrophage polarization. Treatment of inflammatory diseases with rhubarb roots has been described to significantly decrease TLR2, TLR4 and NF-κB mRNA, and protein expression *in vivo* [58,59], which is in line with our results. Emodin, which has been isolated from rhizomes of *Rheum palmatum*, inhibits several target molecules in inflammation and cancer, such as NF-κB and the serine/threonine kinase Akt, both important molecules in TLR2 and TLR4 signaling pathways [60]. Furthermore, emodin suppressed LPS-induced pro-inflammatory cytokines and chemokines as well as NF-κB inhibitor alpha (IκBα) degradation and thus NF-κB activation by disruption of lipid rafts [61]. Further research has to be conducted on whether the inhibition of TLR2- and TLR4 mediated signaling is only related to the reported effects of emodin or if further compounds present in the complex mixture of rhubarb root extract play a role in the observed anti-inflammatory effects.

### Cinnamon (*Cinnamomum verum)–*bark

Cinnamon bark extract strongly mitigated pro-inflammatory cytokine production in stimulated TLR2 and TLR4 signaling pathways, and in LPS-induced nuclear NF-κB translocation. For cinnamon extract, several beneficial health effects are reported in literature, e.g. anti-inflammatory effects on murine alcohol-induced steatosis and colitis [62–64]. Ethanolic cinnamon bark extracts have been shown to suppress release of TNF-α, IL-1β and IL-6 and NF-κB activation, in line with our data [65,66]. The major constituent present in cinnamon bark extract, *trans*-cinnamaldehyde, showed several anti-inflammatory effects [67–70]. Still, more active compounds may be present in cinnamon extract.

### Bilberries (*Vaccinium myrtillus)–*berries

Bilberry extract mitigated LPS-induced inflammatory signals, whereas LPS-induced NF-κB translocation was not affected. Bilberry extract, which is rich in antioxidant anthocyanins, showed anti-inflammatory effects *in vivo*, e.g. suppression of LPS-induced inducible nitric oxide synthase (iNOS), TNF-α, IL-1β and IL-6 transcripts, and iNOS, TNF-α and NF-κB protein levels in liver inflammation [71], compatible with our results. In a randomized, controlled dietary intervention for patients with the metabolic syndrome, bilberry supplementation reduced serum high-sensitivity C-reactive protein (CRP), IL-6, IL-12 and LPS levels, and downregulated genes associated with the TLR pathway [72]. Consumption of bilberry juice by patients with an increased risk for cardiovascular disease led to decreased plasma concentrations of CRP, IL-6 and IL-15 [73]. Overall, bilberry consumption may reduce low-grade metabolic inflammation, decreasing the risk of cardiometabolic diseases [72]. In our study, bilberries showed no inhibitory effects on nuclear translocation of NF-κB. In contrast, quercetin, resveratrol and epicatechin, all polyphenols present in bilberry, were shown to inhibit LPS-induced NF-κB activation *in vitro*, which suggests that conflicting effects of compounds in whole bilberry extract may play a significant role [73]. Taken together, both clinical studies demonstrate not only the anti-inflammatory potential of bilberries, but also their oral effectiveness in humans.

In conclusion, we showed that numerous ethanolic herbal extracts mitigate TLR2- and TLR4-dependent signaling and downstream inflammation. Several of them are known as officinal plants, but the underlying molecular mechanisms of their anti-inflammatory effects are often ill-defined or unknown. TLR pathways are of fundamental relevance in diverse inflammatory diseases. With the identification of orally active TLR-and/or NF-κB antagonizing herbal extracts and their active compounds, new promising treatment strategies might be developed. Moreover, a previously unknown attenuation of TLR-induced inflammatory responses by *Castanea sativa* leaves and *Alchemilla vulgaris* plants could be shown in our study. Interestingly, three out of the ten most effective anti-inflammatory extracts showed no inhibition of NF-κB translocation. Further identification of the active compounds of different extracts, investigations concerning their anti-inflammatory activity and their molecular targets within the signaling cascades are necessary. Furthermore, the anti-inflammatory activity demonstrated with our *in vitro* assays might differ in animals and humans due to alterations by digestion, absorption and metabolism of different compounds, as well as excretion of the extracts. Often, the overall efficacy in complex herbal mixtures is not only based on single compound activity, but on the combination of several different compounds. Hereby, not only additive but also synergistic effects were observed [74–76]. Therefore, experiments with combinations of defined compounds can help to elucidate these interactions.

## Supporting information

S1 Table Overview of herbal extracts. **Scientific and common names, taxonomic information, active compounds and classification and reported beneficial health effects.** ↓: downregulation, ↑: upregulation, AChE: acetylcholinesterase, ALP: alkaline phosphatase, ALT: alanine transaminase, AP-1: activator protein 1, AST: aspartate transaminase, CRP: C-reactive protein, CXCL1: chemokine (C-X-C motif) ligand 1, EGCG: epigallocatechin-3-gallate, ERK: extracellular signal-regulated kinase, Fg: Fibrinogen, GSH: glutathione, Hmox1: heme oxygenase (decycling) 1, IBD: inflammatory bowel disease, IFN: interferon, IL: interleukin, iNOS: inducible nitric oxide synthase, IRAK1: IL-1 receptor-associated kinase, IRF: interferon regulatory factor, IκBα: NF-κB inhibitor alpha, JNK: c-Jun N-terminal kinase, LDH: lactate dehydrogenase, LOX: lipoxygenase, MCP-1: monocyte chemoattractant protein-1, MDA: malondialdehyde, MPO: myeloperoxidase, MyD88: myeloid differentiation primary response 88, NF-κB: Nuclear factor-κB, NLRP3: NOD-like receptor family pyrin domain-containing 3, NO: nitric oxide, Nrf2: nuclear factor erythroid 2-related factor 2, PGE2: prostaglandin E2, RAGE: receptor for advanced glycation end products, RANKL: receptor activator of nuclear factor kappa-B ligand, ROS: reactive oxygen species, SOD: superoxide dismutase, TLR4: Toll-like receptor 4, TGFβ1: Transforming growth factor beta 1, TRAF6: TNF receptor-associated factor 6.
(PDF)

S1 Fig Cell viability and anti-inflammatory effects of ethanolic herbal extracts. HeLa-TLR4 cells (red) and THP-1 monocytes (blue) were incubated with extracts (the ten extracts with highest anti-inflammatory potential are displayed in Fig 1, Fig 2 and Fig 3) or vehicle (70% ethanol), followed by stimulation with LPS-EB. Viability was measured using the Alamar Blue Assay was normalized to the negative control (untreated cells). TLR4 receptor activity was measured using Renilla luciferase expression for the HeLa-TLR4 cell line or IL-8 ELISA (pg/ml) for the THP-1 monocytes and was normalized to ethanol-treated cells. Data are displayed as viability (%) in the left graphs and TLR4 activity divided by normalized viability (%) in the right graphs. Data represents means (*n*≥2).
(PDF)

S2 Fig Anti- and pro-inflammatory effect of ethanolic extracts in HeLa-TLR4 reporter cells and THP-1 monocytes. HeLa-TLR4 reporter cells or THP-1 monocytes were incubated with extracts in different concentrations or vehicle (70% ethanol), followed by stimulation with LPS-EB. Viability was measured using Alamar Blue Assay and was normalized to the negative control (*Viability (%)*). TLR4 receptor activity was measured using Renilla luciferase expression for the HeLa-TLR4 cell line or IL-8 ELISA (pg/ml) for THP-1 monocytes and was normalized to ethanol-treated cells (*TLR4-Activity*). Data are displayed as TLR4 stimulation divided by viability and sorted ascending by the following formula: (150—*Viability (%)*) * (2 * *TLR4-Activity* + 100) weighted in a ratio of 2:1 for THP-1 monocytes vs. HeLa-TLR4 cells. Data represents means (*n*≥2).
(PDF)

S3 Fig TLR2 and TLR4 antagonistic effects of ethanolic herbal extracts. HEK-TLR2 cells (purple) and HEK-TLR4 cells (orange) were incubated with extracts (the five extracts with highest anti-inflammatory potential are displayed in Figs 5 and 6) or vehicle (70% ethanol), followed by stimulation of HEK-TLR2 cells with Pam2CSK4 or HEK-TLR4 cells with LPS-EB Ultrapure. Viability was measured using the Alamar Blue Assay was normalized to the respective negative control. TLR2 and TLR4 receptor activity were measured using SEAP production was normalized to ethanol-treated cells. Data are displayed as viability (%) in the left graphs and receptor activity divided by viability (%) in the right graphs. Data represents means (*n*≥2).
(PDF)

S4 Fig TLR2 and TLR4 antagonistic effects of select ethanolic extracts in HEK-TLR2 and HEK-TLR4 cell lines. HEK-TLR2 or HEK-TLR4 cells were incubated with extracts in different concentrations or vehicle (70% ethanol), followed by stimulation of HEK-TLR2 cells with Pam2CSK4 or HEK-TLR4 cells with LPS-EB Ultrapure. Viability was measured using Alamar Blue Assay was normalized to the negative control. TLR2 and TLR4 receptor activity were measured using SEAP production and were normalized to ethanol-treated cells. Data are displayed as receptor activity divided by normalized viability. Data represents means (*n*≥2).
(PDF)
