# Supplementary material for: Screening of herbal extracts for TLR2- and TLR4-dependent anti-inflammatory effects
Source: PLoS One. 2018 Oct 11;13(10):e0203907. doi: 10.1371/journal.pone.0203907 (PMC6181297; doi:10.1371/journal.pone.0203907)
Supplement: S1 Table — Scientific and common names, taxonomic information, active compounds and classification and reported beneficial health effects. ↓: downregulation, ↑: upregulation, AChE: acetylcholinesterase, ALP: alkaline phosphatase, ALT: alanine transaminase, AP-1: activator protein 1, AST: aspartate transaminase, CRP: C-reactive protein, CXCL1: chemokine (C-X-C motif) ligand 1, EGCG: epigallocatechin-3-gallate, ERK: extracellular signal-regulated kinase, Fg: Fibrinogen, GSH: glutathione, Hmox1: heme oxygenase (decycling) 1, IBD: inflammatory bowel disease, IFN: interferon, IL: interleukin, iNOS: inducible nitric oxide synthase, IRAK1: IL-1 receptor-associated kinase, IRF: interferon regulatory factor, IκBα: NF-κB inhibitor alpha, JNK: c-Jun N-terminal kinase, LDH: lactate dehydrogenase, LOX: lipoxygenase, MCP-1: monocyte chemoattractant protein-1, MDA: malondialdehyde, MPO: myeloperoxidase, MyD88: myeloid differentiation primary response 88, NF-κB: Nuclear factor-κB, NLRP3: NOD-like receptor family pyrin domain-containing 3, NO: nitric oxide, Nrf2: nuclear factor erythroid 2-related factor 2, PGE2: prostaglandin E2, RAGE: receptor for advanced glycation end products, RANKL: receptor activator of nuclear factor kappa-B ligand, ROS: reactive oxygen species, SOD: superoxide dismutase, TLR4: Toll-like receptor 4, TGFβ1: Transforming growth factor beta 1, TRAF6: TNF receptor-associated factor 6. (PDF) [file pone.0203907.s001.pdf]

Latin name	Common English name	Common German name	Family	Active compound	Compound classification	Influence on (inflammatory) marker	Beneficial human health effect	Reference
Achillea millefolium	Common yarrow	Gemeine Schafgarbe	*Asteraceae*	Kaempferol, luteolin, apigenin	Flavonoids	↓ ALT, ↓ AST, ↑ GSH, ↓ MPO, ↑ SOD	Ameliorating several neuro-degenerative disorders	Potrich et al., 2010; Yaeesh et al., 2006; Ayoobi et al., 2017
Aesculus hippocastanum	Horse-chestnut	Gewöhnliche Rosskastanie	*Sapindaceae*	Escin	Saponin	↓ IκBα, ↓ NF-κB, ↑ ROS	Prolongation of survival in thyroid cancer patients	Cheong et al., 2018
Alchemilla vulgaris	Common lady's mantle	Spitzlappiger Frauenmantel	*Rosaceae*				Weight loss (when combined with other plants)	Said et al., 2011
Allium sativum	Garlic	Knoblauch	*Amaryllidaceae*	Diallyl sulfide	Sulfide	↓ ALT, ↓ AST, ↓ COX-2, ↓ IL-1β, ↓ iNOS, ↑ insulin, ↓ NF-κB, ↓ TNF-α	Reduction of body fat mass and body weight	Eidi et al., 2006; Soleimani et al., 2016; Suman and Shukla, 2016
Aloe ferox	Aloe	Aloen	*Xanthorrhoeaceae*	Aloeresin I	Glucoside	↓ caspase-1, ↓ COX-1, ↓ ERK, ↓ IL-1β, ↓ IL-6, ↓ IL-8, ↓ JNK, ↓ NF-κB, ↓ NLRP3, ↓ p38, ↓ TNF-α	Wound healing	Speranza et al., 2005; Fawole et al., 2010; Budai et al., 2013; Radha and Laxmipriya, 2014
Alpinia officinarum	Galangal	Galgant	*Zingiberaceae*	Galangin	Flavonoid	↓ IL-6, ↑ IL-10, ↓ NO, ↓ ROS, ↓ TNF-α		Choi et al., 2017
Althaea officinalis	Common marshmallow	Eibisch	*Malvaceae*				Treatment of Old World cutaneous leishmaniasis; Cough reduction	Cravotto et al., 2010

Latin name	Common English name	Common German name	Family	Active compound	Compound classification	Influence on (inflammatory) marker	Beneficial human health effect	Reference
Arctostaphylos uva-ursi	Bearberry	Bärentrauben	*Ericaceae*	Arbutin	Glucoside	↓ COX-2, ↓ IL-1β, ↓ iNOS, ↓ NF-κB, ↓ NO, ↓ TNF-α		Lee and Kim, 2012
Armoracia rusticana	Horseradish	Meerrettich	*Brassicaceae*			↓ c-Jun, ↓ COX-2, ↓ ERK1/2, ↓ IL-6, ↓ NO, ↓ PGE_2, ↓ TNF-α		Marzocco et al., 2015; Herz et al., 2017
Arnica montana	Arnica	Arnika	*Asteraceae*	Helenalin	Sesquiterpene	↓ IL-1β, ↓ IL-6, ↓ IL-12, ↓ NF-κB, ↓ NO, ↓ TNF-α	Osteoarthritis	Lass et al., 2008; Chahal et al., 2013; Dragos et al., 2017
Artemisia absinthium	Wormwood	Wermutkraut	*Asteraceae*		Flavonoids	↓ COX-2, ↓ iNOS, ↓ NF-κB, ↓ NO, ↓ PGE_2, ↓ TNF-α	Crohn's disease	Lee et al., 2004; Omer et al., 2007; Krebs et al., 2010
Avena sativa	Oat	Saat-Hafer	*Poaceae*	β-Glucan	Glucan	↓ COX-2, ↓ NO, ↑ TGFβ1, ↓ TNF-α	Anti-diabetic, prevention of cardiovascular diseases, protection against breast cancer	Singh et al., 2013
Boswellia carterii	Frankincense	Afrikanischer Weihrauch	*Burseraceae*	Boswellic acid	Triterpene	↓ 5-LOX, ↓ AChE, ↑ GSH, ↓ MDA		Safayhi et al., 2000; Ebrahimpour et al., 2017
Boswellia serrata	Frankincense	Weihrauch	*Burseraceae*	Boswellic acid	Triterpene	↓ 5-LOX, ↓ IFNγ, ↓ IL-1β, ↓ IL-2, ↑ IL-4, ↓ IL-6, ↑ IL-10, ↓ IL-12, ↓ NO, ↓ TNF-α	Osteoarthritis	Safayhi et al., 2000; Gayathri et al., 2007; Dragos et al., 2017
Calendula officinalis	Marigold	Ringelblume	*Asteraceae*		Several triterpenes and flavonoids	↓ COX-2, ↓ IL-1(β), ↓ TNF-α	Wound healing; Prevention of acute dermatitis	Ukiya et al., 2006; Cravotto et al., 2010; Kirichenko et al., 2016; Alexandre et al., 2017

Latin name	Common English name	Common German name	Family	Active compound	Compound classification	Influence on (inflammatory) marker	Beneficial human health effect	Reference
Camellia sinensis (L.)	Green tea	Grüner Tee	*Theaceae*	EGCG	Flavonoid	↓ COX-2, ↓ IFNγ, ↓ NF-κB, ↑ ROS, ↓ TNF-α	Metabolism, cardiovascular system, neuro-degenerative diseases and cancer	Cooper et al., 2005; Arab et al., 2009; Shirakami et al., 2016
Capsicum frutescens	Chili	Chili	*Solanaceae*	Capsaicin	Polyunsaturated alkamide	↓ AP-1, ↓ IκBα, ↓ NF-κB	Pain reduction	Han et al., 2001; Bortolotti and Porta, 2011; Gagnier et al., 2016
Carum carvi	Caraway	Echter Kümmel	*Apiaceae*			↓ cholesterol, ↓ triglycerides	Anti-obesity	Lemhadri et al., 2006; Kazemipoor et al., 2013
Castanea sativa	Sweet chestnut	Edelkastanie	*Fagaceae*		Phenols	↓ NF-κB, ↓ RAGE, ↓ $O_2^{\cdot-}$, ↓ ·OH		Calliste et al., 2005; Jovanović et al., 2017
Chelidonium majus	Celandine	Schöllkraut	*Papaveraceae*	Berberine, norchelidonine, chelidonine, 8-hydroxydihydro-sanguinarine	Alkaloids	↓ COX-2, ↓ iNOS, ↓ NO, ↓ PGE_2	Anticancer drug (Ukrain) based on plant extract	Kuo et al., 2004; Ernst and Schmidt, 2005; Park et al., 2011
Chlorella pyrenoidosa	Chlorella	Chlorella	*Chlorellaceae*	Rhamnose, glucose, galactose, mannose, xylose	Mono-saccharides	↑ IL-1β	Pregnant and breastfeeding women	Nakano et al., 2007; Nakano et al., 2010; Chahal et al., 2013
Cinchona pubescens	Cinchona	Chinarinde	*Rubiaceae*	Quinine	Quinoline	↓ NF-κB	Malaria treatment	An et al., 2017
Cinnamomum verum	Cinnamon	Zimt	*Lauraceae*	Cinnamaldehyde	Flavonoid	↓ IκB, ↓ iNOS, ↓ MyD88, ↓ NF-κB, ↓ TNF-α	Anti-hyperlipidaemic and blood pressure lowering, stimulation of glycogen synthesis and insulin secretion	Khan et al., 2003; Kanuri et al., 2009; Hariri and Ghiasvand, 2016; Ranasinghe et al., 2017

Latin name	Common English name	Common German name	Family	Active compound	Compound classification	Influence on (inflammatory) marker	Beneficial human health effect	Reference
Coriandrum sativum	Coriander	Echter Koriander	*Apiaceae*			↓ COX-2, ↓ IL-1β, ↓ iNOS, ↓ NO, ↓ PGE_2	Reduction of UV-induced erythema	Reuter et al., 2008; Wu et al., 2010
Crataegus species	Hawthorn	Weißdorn	*Rosaceae*	Vitexin	Flavonoid	↓ IL-1(β), ↑ IL-10, ↓ NO, ↓ p-ERK1/2, ↓ PGE_2, ↓ p-JNK, ↓ p-p38, ↓ TNF-α	Adjunctive treatment for chronic heart failure; Hypotensive and hypolipidemic effects	Pittler et al., 2003; Walker et al., 2006; Dalli et al., 2011; Kirichenko et al., 2016; Rosa et al., 2016
Curcuma longa	Turmeric	Kurkuma	*Zingiberaceae*	Curcumin	Catechol	↓ AP-1, ↓ COX-2, ↓ IFNγ, ↓ IL-1, ↓ IL-6, ↓ IL-8, ↓ IL-12, ↓ iNOS, ↓ JNK, ↓ NF-κB, ↓ RANKL, ↓ TNF-α	Suppression of IBD symptoms; Osteoarthritis	Joe et al., 2004; Goel et al., 2008; Pari et al., 2008; Baliga et al., 2012; Dragos et al., 2017
Cynara scolymus	Artichoke	Artischocken	*Asteraceae*			↓ CRP, ↓ Fg, ↓ MDA	Decreased cholesterol level	Bundy et al., 2008; Ben Salem et al., 2017
Daucus carota subsp. *sativus*	Carrot	Karotte	*Apiaceae*	*trans*-Asarone<br>2,4,5-Trimethoxybenz-aldehyde	Anisole<br>Benzaldehyde	↓ COX-2		Momin et al., 2003
Digitalis purpurea	Common foxglove	Roter Fingerhut	*Plantaginaceae*	Digoxin	Digitalis glycoside		Cardiac disorders	Hauptman and Kelly, 1999
Dioscorea villosa	Yam	Yams	*Dioscoreaceae*	Diosgenin	Cholestane	↓ p-AKT, ↓ p-ERK, ↓ p-JNK, ↓ p-p38, ↓ ROS, ↓ TNF-α		Choi et al., 2010
Echinacea purpurea	Purple coneflower	Purpur-Sonnenhut	*Asteraceae*		Alkamides	↓ COX-1, ↓ COX-2, ↓ IL-1β, ↓ IL-6, ↓ IL-8, ↓ NO, ↓ TNF-α		Senchina et al., 2006; Woelkart and Bauer, 2007; Sharma et al., 2009

Latin name	Common English name	Common German name	Family	Active compound	Compound classification	Influence on (inflammatory) marker	Beneficial human health effect	Reference
Elettaria cardamomum	Cardamom	Kardamom	*Zingiberaceae*	Cardamonin	Flavonoid	↓ COX-2, ↓ IL-1β, ↓ IL-6, ↓ iNOS, ↓ NF-κB, ↓ NO, ↓ PGE_2, ↓ TNF-α		Prasad and Aggarwal, 2014
Equisetum arvense	Field horsetail	Acker-Schachtelhalm	*Equisetaceae*	Kynurenic acid	Quinoline	↓ IFNγ, ↓ TNF-α	Rheumatoid arthritis	Gründemann et al., 2014; Dragos et al., 2017
Erythraea centaurium	Common centaury	Echtes Tausendgülden-kraut	*Gentianaceae*			↓ ALT, ↓ AST, ↓ LDH		Mroueh et al., 2004
Euphrasia officinalis	Eyebright	Gemeiner Augentrost	*Orobanchaceae*			↓ IL-1β, ↓ IL-6, ↓ TNF-α		Paduch et al., 2014
Filipendula ulmaria	Meadowsweet	Mädesüß	*Rosaceae*	Quercetin	Flavonoid	↓ COX-1, ↓ COX-2, ↓ IL-1β, ↓ IL-6, ↓ TNF-α		Drummond et al., 2013; Katanić et al., 2016
Foeniculum vulgare	Fennel	Fenchel	*Apiaceae*	Limonene<br>*trans*-Anethole	Cyclohexene<br>Anisole	↓ IL-6, ↓ LDH, ↓ NO, ↓ p-ERK, ↓ TNF-α		Lee et al., 2015
Fucus vesiculosus	Bladderwrack	Blasentang	*Fucaceae*	Fucoidan	Polysaccharide	↓ COX-2, ↓ NO		Lim et al., 2015
Gentiana lutea	Gentian	Enzian	*Gentianaceae*	Gentiopicroside	Glucoside	↓ ERK 1/2, ↓ iNOS, ↓ MPO, ↓ NO		Nastasijević et al., 2012; Kesavan et al., 2013
Geranium robertianum	Herb robert	Ruprechtskraut	*Geraniaceae*			↓ NO		Catarino et al., 2017
Ginkgo biloba	Ginkgo	Ginkgo	*Ginkgoaceae*	Bilobalide	Ginkgolide	↓ ERK1/2	Enhancing certain neuro-psychological / memory processes	Mix and Crews, 2002; Dodge et al., 2008; Weinmann et al., 2010; Chahal et al., 2013
Glycyrrhiza glabra	Liquorice	Süßholz	*Fabaceae*	Isoliquiritigenin	Flavonoid	↓ COX-2, ↓ iNOS, ↓ IRF3, ↓ NF-κB	Decrease of mucositis and oral mucosal irritation	Park and Youn, 2010; Najafi et al., 2017

Latin name	Common English name	Common German name	Family	Active compound	Compound classification	Influence on (inflammatory) marker	Beneficial human health effect	Reference
Hamamelis virginiana	Witch hazel	Hamamelis	*Hamamelidaceae*	Hamamelitannin	Benzoate	↓ TNF-α	Erythema suppression	Hughes-Formella et al., 1998; Habtemariam, 2002
Harpagophytum procumbens	Devil's claw	Afrikanische Teufelskralle	*Pedaliaceae*	Harpagoside, harpagide, procumbide	Iridoid glycosides	↓ c-FOS, ↓ COX-2, ↓ IL-1β, ↓ iNOS, ↓ NF-κB, ↓ TNF-α	Osteoarthritis and low back pain	Gagnier et al., 2004; Rodriguez Villanueva et al., 2016; Dragos et al., 2017
Hedera helix	Common ivy	Efeu	*Araliaceae*	α-Hederin	Saponin	↓ IL-17		Ebrahimi et al., 2016
Hibiscus sabdariffa	Roselle	Roselle	*Malvaceae*		Polyphenols	↓ COX-2, ↓ ERK1/2, ↓ IFNγ, ↓ IL-6, ↑ IL-10, ↓ JNK, ↓ NF-κB, ↓ NO, ↓ p38, ↓ PGE_2, ↓ ROS, ↓ TNF-α		Da-Costa-Rocha et al., 2014; Herranz-López et al., 2017
Humulus lupulus	Hops	Hopfen	*Cannabaceae*	Xanthohumol	Flavonoid	↓ IL-1β, ↓ MCP-1, ↓ NF-κB, ↓ NO, ↓ TNF-α		Lupinacci et al., 2009; Lee et al., 2011
Hypericum perforatum	St John's wort	Echtes Johanniskraut	*Hypericaceae*	Pseudohypericin<br>Amentoflavone, quercetin<br>Chlorogenic acid	Benz(a)-anthracene<br>Flavonoids<br>Cyclohexane	↓ COX-2, ↓ IL-1, ↓ IL-6, ↓ NO, ↓ PGE_2, ↓ TNF-α	Depression	Schulz, 2006; Kasper et al., 2010; Huang et al., 2012; Kirichenko et al., 2016; Bonaterra et al., 2018
Ilex paraguariensis	Yerba mate	Mate-Strauch	*Aquifoliaceae*	Quercetin	Flavonoid	↓ COX-2, ↑ IκBα, ↓ iNOS, ↓ NF-κB, ↓ TNF-α		Arçari et al., 2011; Bracesco et al., 2011; Pimentel et al., 2013
Juniperus communis	Common juniper	Gemeiner Wacholder	*Cupressaceae*	Amentoflavone	Flavonoid	↓ ALT, ↓ ALP, ↓ AST, ↓ TNF-α		Bais et al., 2017
Marrubium vulgare	Common horehound	Andornkraut	*Lamiaceae*	Marrubiin	Diterpene	↓ COX-2		Sahpaz et al., 2002; Meyre-Silva et al., 2005

Latin name	Common English name	Common German name	Family	Active compound	Compound classification	Influence on (inflammatory) marker	Beneficial human health effect	Reference
Matricaria chamomilla	Chamomile	Kamille	*Asteraceae*	Apigenin	Flavonoid	↓ IL-1β, ↓ IL-6, ↓ TNF-α		Drummond et al., 2013
Melilotus officinalis	Sweet clover	Steinkleekraut	*Fabaceae*	Caffeic acid	Cinnamate	↓ COX-2, ↓ IL-6, ↓ iNOS, ↓ NF-κB, ↓ NO, ↓ PGE_2, ↓ TNF-α		Bazazzadegan et al., 2017; Liu et al., 2018
Melissa officinalis	Lemon balm	Zitronenmelisse	*Lamiaceae*	Rosmarinic acid	Benzoate	↓ IL-1β, ↓ IL-6, ↓ IRAK1, ↓ MyD88, ↓ TLR4, ↓ TNF-α, ↓ TRAF6		Jiang et al., 2017; Chizzola et al., 2018
Mentha piperita	Peppermint	Pfefferminze	*Lamiaceae*			↓ PGE_2, ↓ NO		Sun et al., 2014
Origanum majorana	Marjoram	Majoran	*Lamiaceae*	Rosmarinic acid	Benzoate	↓ IL-1β, ↓ IL-6, ↓ TNF-α		Villalva et al., 2018
Panax ginseng	Ginseng	Ginseng	*Araliaceae*	Ginsan	Polysaccharide Saponins	↓ COX-2, ↓ IFNγ, ↓ IL-1β, ↓ IL-6, ↓ IL-12, ↓ IL-18, ↓ iNOS, ↓ MyD88, ↓ TLR4, ↓ TNF-α	Osteoarthritis; Improvement of glucose metabolism and modulating of immune response	Kang and Min, 2012; Chahal et al., 2013; Shergis et al., 2013; Dragos et al., 2017
Petroselinum crispum	Parsley	Petersilie	*Apiaceae*	Apigenin	Flavonoid	↓ NO	Increase in anti-oxidant enzymes	Nielsen et al., 1999; Farzaei et al., 2013
Pimpinella anisum	Anise	Anis	*Apiaceae*	Anethole	Anisole	↓ AP-1, ↑ IL-10, ↓ NF-κB, ↓ p38, ↓ TNF-α		Aprotosoaie et al., 2016
Plantago lanceolata	Ribwort	Spitzwegerich	*Plantaginaceae*	Acteoside	Phenol glycoside	(↓ COX-1), (↓ COX-2), ↓ IL-8, ↓ iNOS, ↓ MCP-1, ↓ NO		Murai et al., 1995; Vigo et al., 2005; Fakhrudin et al., 2017
Rheum palmatum	Rhubarb	Rhabarber	*Polygonaceae*	Rhein, emodin	Anthraquinones	↓ TNF-α		Chahal et al., 2013
Rosmarinus officinalis	Rosemary	Rosmarin	*Lamiaceae*	Rosmarinic acid	Benzoate	↓ IL-1β, ↓ IL-6, ↑ NF-κB, ↓ TNF-α		Rocha et al., 2015

Latin name	Common English name	Common German name	Family	Active compound	Compound classification	Influence on (inflammatory) marker	Beneficial human health effect	Reference
Rubus fruticosus	Blackberry	Brombeere	*Rosaceae*	Cyanidin-3-O-glucoside	Anthocyanin	↓ iNOS, ↓ NF-κB, ↓ NO		Pergola et al., 2006; Zia-Ul-Haq et al., 2014
Salix alba	White willow	Silber-Weide	*Salicaceae*	Salicin	Glucoside	↓ COX-2, ↓ IL-6, ↓ NF-κB, ↓ TNF-α	Pain relief	Drummond et al., 2013; Shara and Stohs, 2015; Dragos et al., 2017
Salvia officinalis	Salvia	Salbei	*Lamiaceae*		Different flavonoids and terpenes	↓ MDA, ↓ NF-κB, ↓ NO, ↓ TNF-α		Ghorbani and Esmaeilizadeh, 2017; Kolac et al., 2017
Sambucus nigra (L.)	Elderflowers	Holunder	*Adoxaceae*		Anthocyanins	↓ IFNγ, ↓ IL-1, ↓ TNF-α		Badescu et al., 2015; Kirichenko et al., 2016
Schinus terebinthifolius	Brazilian pepper tree	Brasilianischer Pfefferbaum	*Anacardiaceae*			↓ CXCL1, ↓ IL-1β, ↓ IL-6, ↓ TNF-α	Reduction of gingival inflammation	Freires et al., 2013; Rosas et al., 2015
Spirulina	Spirulina	Spirulina	*Spirulinaceae*	Phycocyanin	Phycobiliprotein	↓ IL-6, ↑ Nrf2, ↓ TNF-α		Khafaga and El-Sayed, 2018; Kim et al., 2018
Symphytum officinale	Comfrey	Echter Beinwell	*Boraginaceae*	Rosmarinic acid Salvianolic acid	Benzoate Cinnamate	↓ IL-1β, ↓ IL-6, ↓ NF-κB, ↓ NO, ↓ TNF-α	Reduction of pain, inflammation and swelling	Koll et al., 2004; Giannetti et al., 2010; Staiger, 2013; Dragos et al., 2017; Trifan et al., 2018
Syzygium aromaticum	Clove	Nelken	*Myrtaceae*	Eugenol	Cinnamate	↓ COX-2, ↓ NF-κB	Local anti-inflammatory, antiseptic, and anesthetic effects on dental pulp	Fujisawa and Murakami, 2016
Tanacetum parthenium	Feverfew	Mutterkraut	*Asteraceae*	Tanetin<br>Parthenolide	Flavonoid<br>Sesquiterpene	↓ 5-LOX, ↓ COX-2, ↓ IL-6, ↓ IL-8, ↓ iNOS, ↓ NF-κB, ↓ NO, ↓ TNF-α		Williams et al., 1995; Mazor et al., 2000; Aviram et al., 2012; Magni et al., 2012
Taraxacum officinale	Dandelion	Löwenzahn	*Asteraceae*	Taraxinic acid β-d-glucopyranosyl	Sesquiterpene	↑ Hmox1, ↓ iNOS,		Liu et al., 2014; Esatbeyoglu et al.,

Latin name	Common English name	Common German name	Family	Active compound	Compound classification	Influence on (inflammatory) marker	Beneficial human health effect	Reference
				ester		↓ NF-κB, ↑ Nrf2		2017
Thymus vulgaris	Common thyme	Thymian	*Lamiaceae*	Thymol	Monoterpene	↓ IL-1β, ↓ TNF-α		Amirghofran et al., 2011; Oliveira et al., 2017
Tropaeolum majus	Nasturtium	Kapuzinerkresse	*Tropaeolaceae*	Benzyl glucosinolate	Isothiocyanate	↓ COX-2, ↓ ERK1/2, ↓ JNK, ↓ PGE_2		Rodriguez Villanueva et al., 2016
Uncaria tomentosa	Cat's claw	Katzenkralle	*Rubiaceae*	Mitraphylline	Indole alkaloid	↓ c-Fos, ↓ c-Jun, ↓ IL-1α, ↓ IL-1β, ↓ IL-4, ↓ IL-17, ↓ Jun-B, ↓ Jun-D, ↓ TNF-α	Adjuvant treatment for reducing adverse chemotherapy effects	Sandoval et al., 2000; Allen-Hall et al., 2010; Rojas-Duran et al., 2012; Santos Araújo et al., 2012
Urtica dioica	Stinging nettle	Große Brennnessel	*Urticaceae*	Hydroxycinnamic acid, chlorogenic acid	Cinnamate	↓ 12-LOX, ↓ COX-1, ↓ COX-2, ↓ MCP-1, ↓ NO		Carvalho et al., 2017; Franciškovič et al., 2017
Usnea barbata	Barber's itch	Bartflechte	*Parmeliaceae*	Usnic acid	Benzofuran	↓ PGE_2		Engel et al., 2007; Ranković et al., 2012
Vaccinium myrtillus	Bilberry	Heidelbeere	*Ericaceae*		Anthocyanins	↓ COX-2, ↓ IL-6, ↓ IL-12, ↓ iNOS	Different inflammatory processes	Sautebin et al., 2004; Karlsen et al., 2010; Kolehmainen et al., 2012
*Valeriana officinalis (*L.*)*	Common valerian	Echter Baldrian	*Caprifoliaceae*	Valerenic acid	Sesquiterpene	↓ MDA		Nam et al., 2013
Vanilla planifolia	Vanilla	Gewürzvanille	*Orchidaceae*	Vanillin	Benzaldehyde	↑ GSH, ↓ MDA, ↓ NF-κB, ↓ TNF-α		Elseweidy et al., 2017
Verbena officinalis	Common vervain	Echtes Eisenkraut	*Verbenaceae*					
Vigna radiata	Mung bean (dried)	Mungbohnen (getrocknet)	*Fabaceae*	Gallic acid<br>Vitexin, isovitexin	Benzoate<br>Flavonoids	↓ IL-1β, ↓ IL-6, ↓ IL-12β, ↓ iNOS, ↓ TNF-α		Tang et al., 2014
Vigna radiata	Mung bean (cooked in boiling water for	Mungbohnen (gekocht)	*Fabaceae*	Gallic acid<br>Vitexin, isovitexin	Benzoate<br>Flavonoids	↓ IL-1β, ↓ IL-6, ↓ IL-12β,		Tang et al., 2014

Latin name	Common English name	Common German name	Family	Active compound	Compound classification	Influence on (inflammatory) marker	Beneficial human health effect	Reference
	20 min)					↓ iNOS, ↓ TNF-α		
Viscum album	European mistletoe	Weißbeerige Mistel	*Santalaceae*		Diarylheptanoids	↓ IL-6, ↓ IL-12, ↓ TNF-α	Quality of life and reduction of side effects in cancer	Kienle and Kiene, 2010; Büssing et al., 2012; Nhiem et al., 2013
Zingiber officinale	Ginger	Ingwer	*Zingiberaceae*	6-Gingerol, 6-shogaol	Catechols	↓ AP-1, ↓ COX-1, ↓ COX-2, ↓ IL-1β, ↓ IL-12, ↓ IκBα, ↓ LOX, ↓ NF-κB, ↓ TNF-α	Antiemetic and inhibition of nausea and vomiting in pregnancy; Beneficial effects in osteoarthritis	Prasad and Aggarwal, 2014; Mohd Yusof, 2016; Dragos et al., 2017
