# Supplementary material for: Screening of herbal extracts for TLR2- and TLR4-dependent anti-inflammatory effects
Source: PLoS One. 2018 Oct 11;13(10):e0203907. doi: 10.1371/journal.pone.0203907 (PMC6181297; doi:10.1371/journal.pone.0203907)
Supplement: S1 Fig — HeLa-TLR4 cells (red) and THP-1 monocytes (blue) were incubated with extracts (the ten extracts with highest anti-inflammatory potential are displayed in Fig 1, Fig 2 and Fig 3) or vehicle (70% ethanol), followed by stimulation with LPS-EB. Viability was measured using the Alamar Blue Assay was normalized to the negative control (untreated cells). TLR4 receptor activity was measured using Renilla luciferase expression for the HeLa-TLR4 cell line or IL-8 ELISA (pg/ml) for the THP-1 monocytes and was normalized to ethanol-treated cells. Data are displayed as viability (%) in the left graphs and TLR4 activity divided by normalized viability (%) in the right graphs. Data represents means (n≥2). (PDF) [file pone.0203907.s002.pdf]

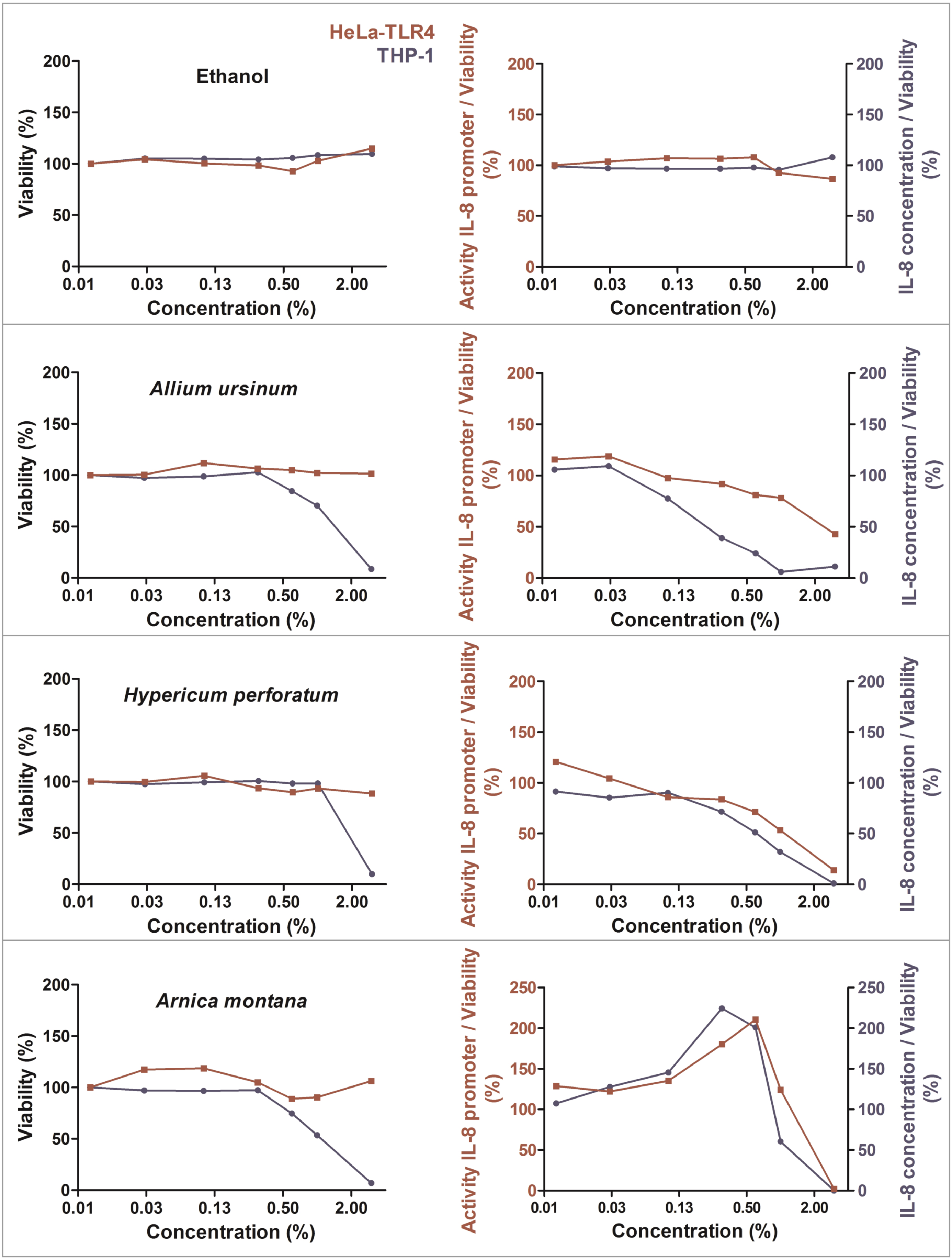

HeLa-TLR4
THP-1
Ethanol
Allium ursinum
Hypericum perforatum
Arnica montana
Viability (%)
Activity IL-8 promoter / Viability (%)
IL-8 concentration / Viability (%)
Concentration (%)
0.01
0.03
0.13
0.50
2.00
0
50
100
150
200
250

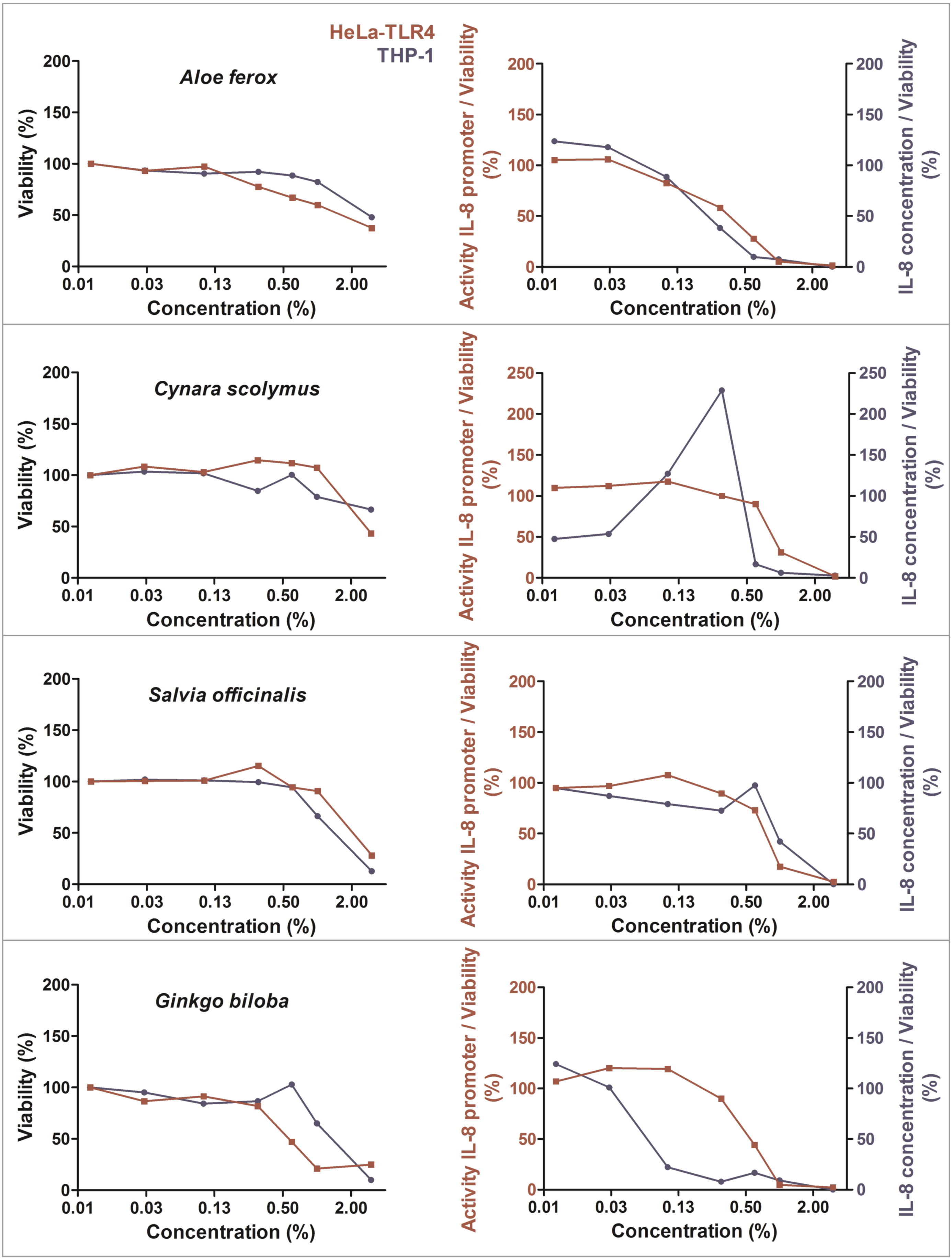
HeLa-TLR4
THP-1
Aloe ferox
Cynara scolymus
Salvia officinalis
Ginkgo biloba
Viability (%)
Activity IL-8 promoter / Viability (%)
IL-8 concentration / Viability (%)
Concentration (%)
0.01
0.03
0.13
0.50
2.00

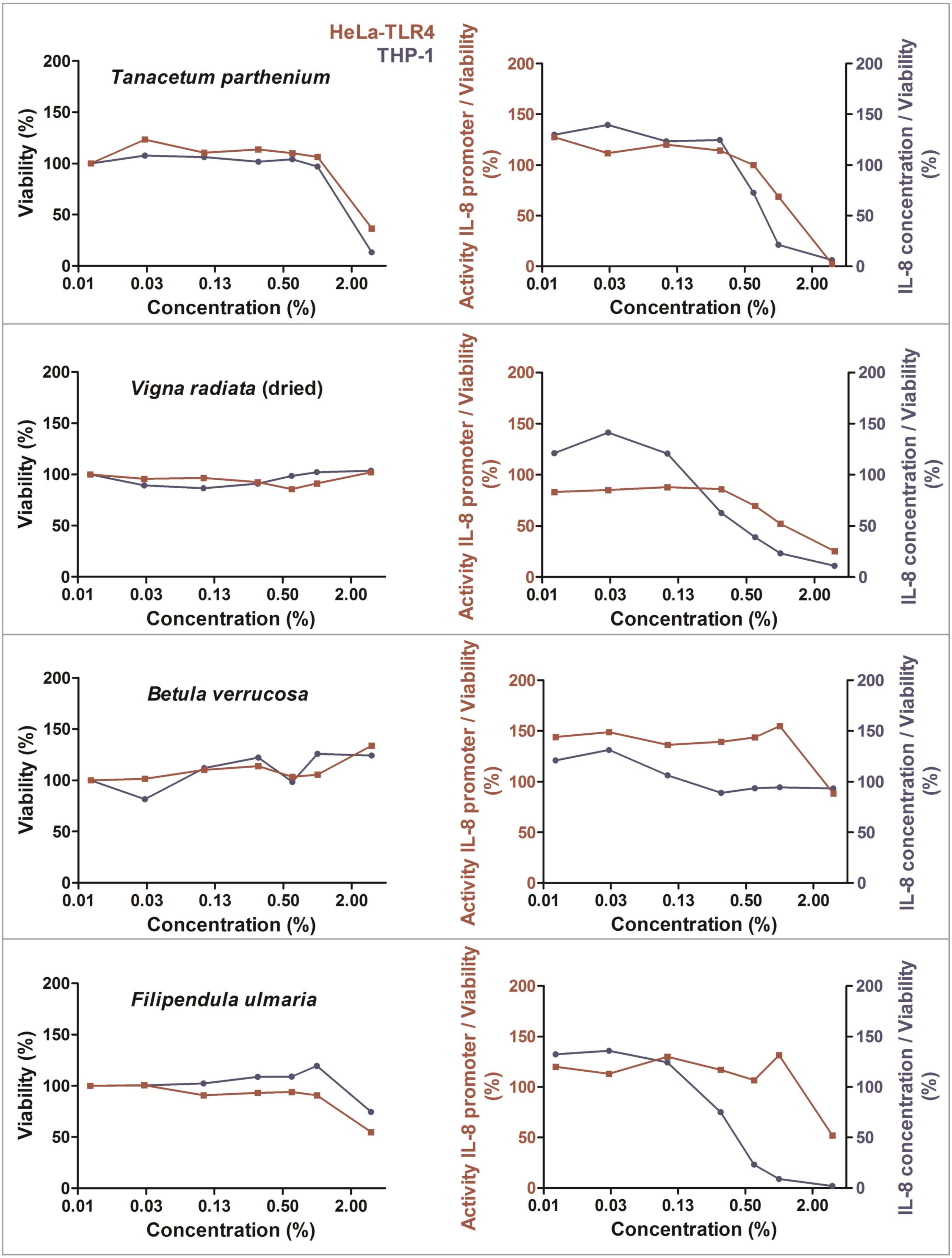

HeLa-TLR4
THP-1
Tanacetum parthenium
Vigna radiata (dried)
Betula verrucosa
Filipendula ulmaria
Viability (%)
Concentration (%)
Activity IL-8 promoter / Viability (%)
IL-8 concentration / Viability (%)
0.01
0.03
0.13
0.50
2.00
0
50
100
150
200

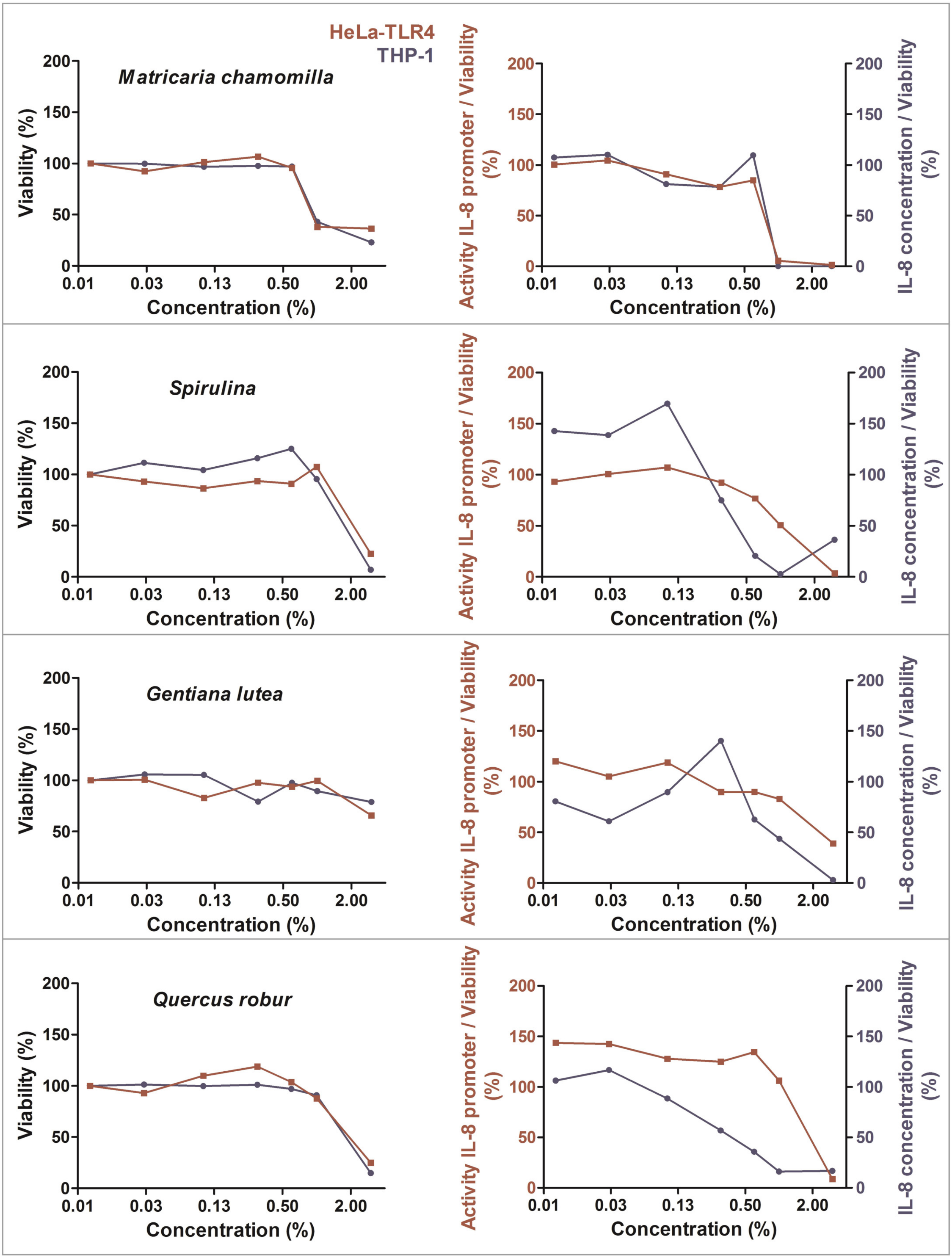
HeLa-TLR4
THP-1
Matricaria chamomilla
Spirulina
Gentiana lutea
Quercus robur
Viability (%)
Concentration (%)
Activity IL-8 promoter / Viability (%)
IL-8 concentration / Viability (%)
0
50
100
150
200
0.01
0.03
0.13
0.50
2.00

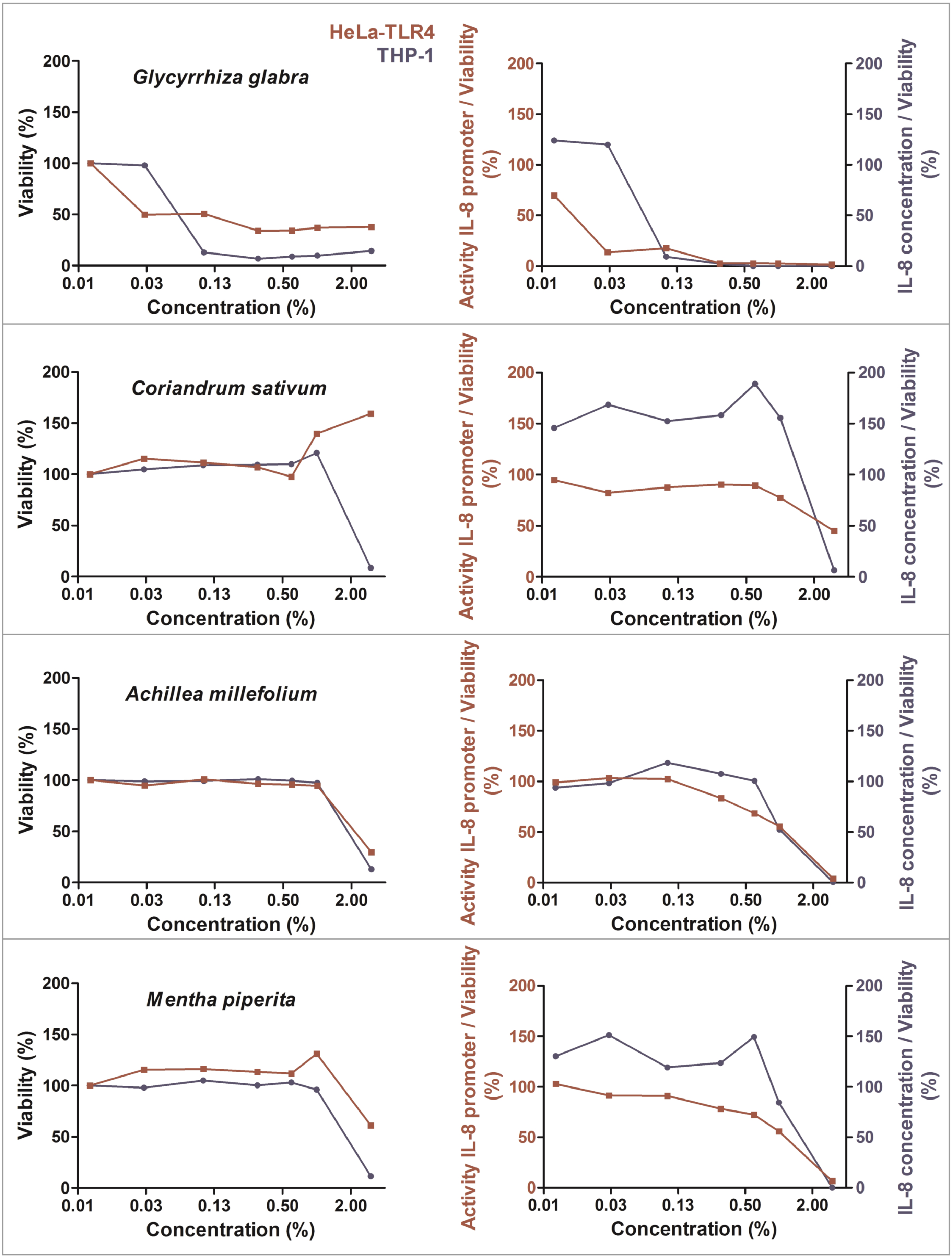

HeLa-TLR4
THP-1
Glycyrrhiza glabra
Coriandrum sativum
Achillea millefolium
Mentha piperita
Viability (%)
Concentration (%)
Activity IL-8 promoter / Viability (%)
IL-8 concentration / Viability (%)
0.01
0.03
0.13
0.50
2.00
0
50
100
150
200

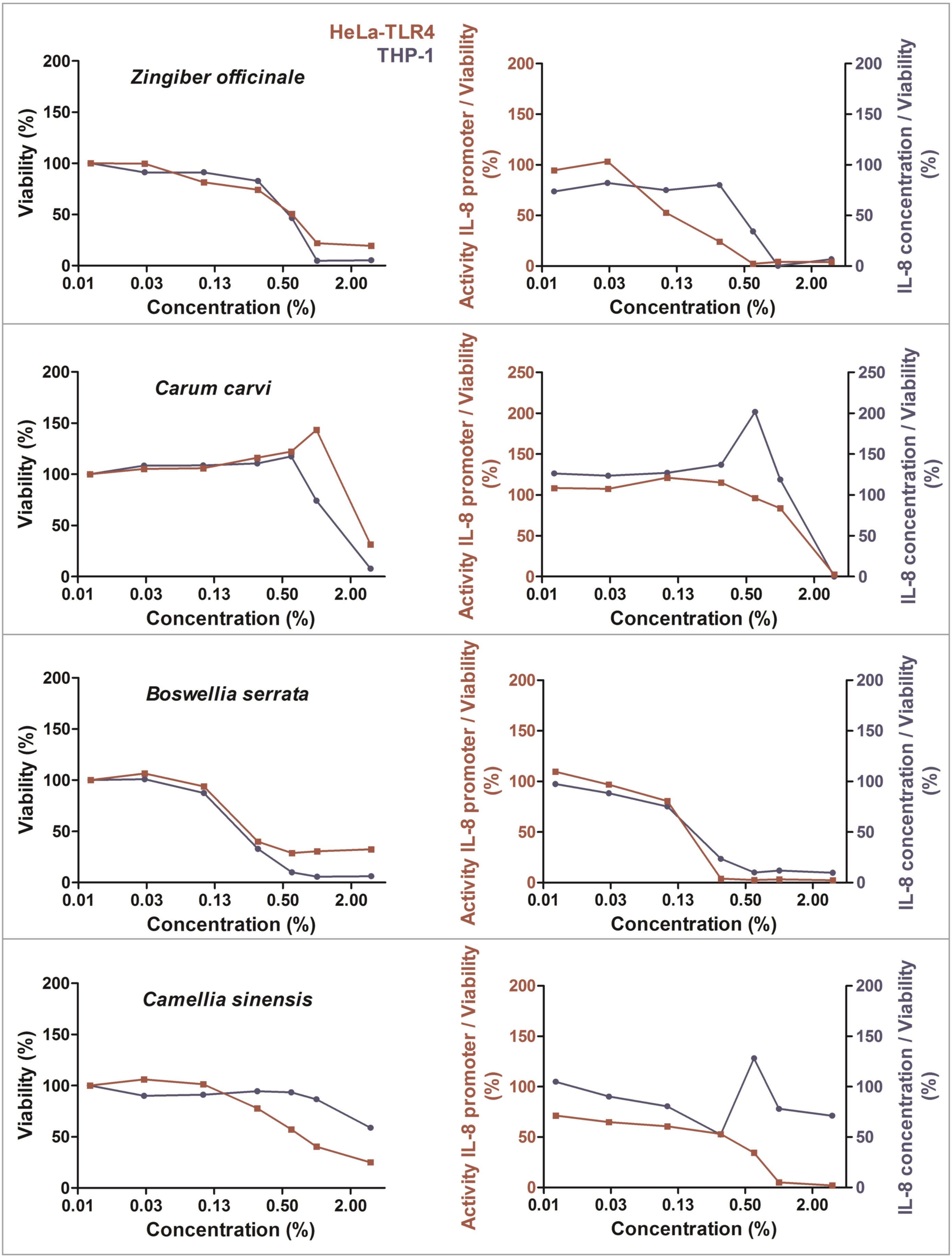
HeLa-TLR4
THP-1
Zingiber officinale
Carum carvi
Boswellia serrata
Camellia sinensis
Viability (%)
Concentration (%)
Activity IL-8 promoter / Viability (%)
IL-8 concentration / Viability (%)
0.01
0.03
0.13
0.50
2.00

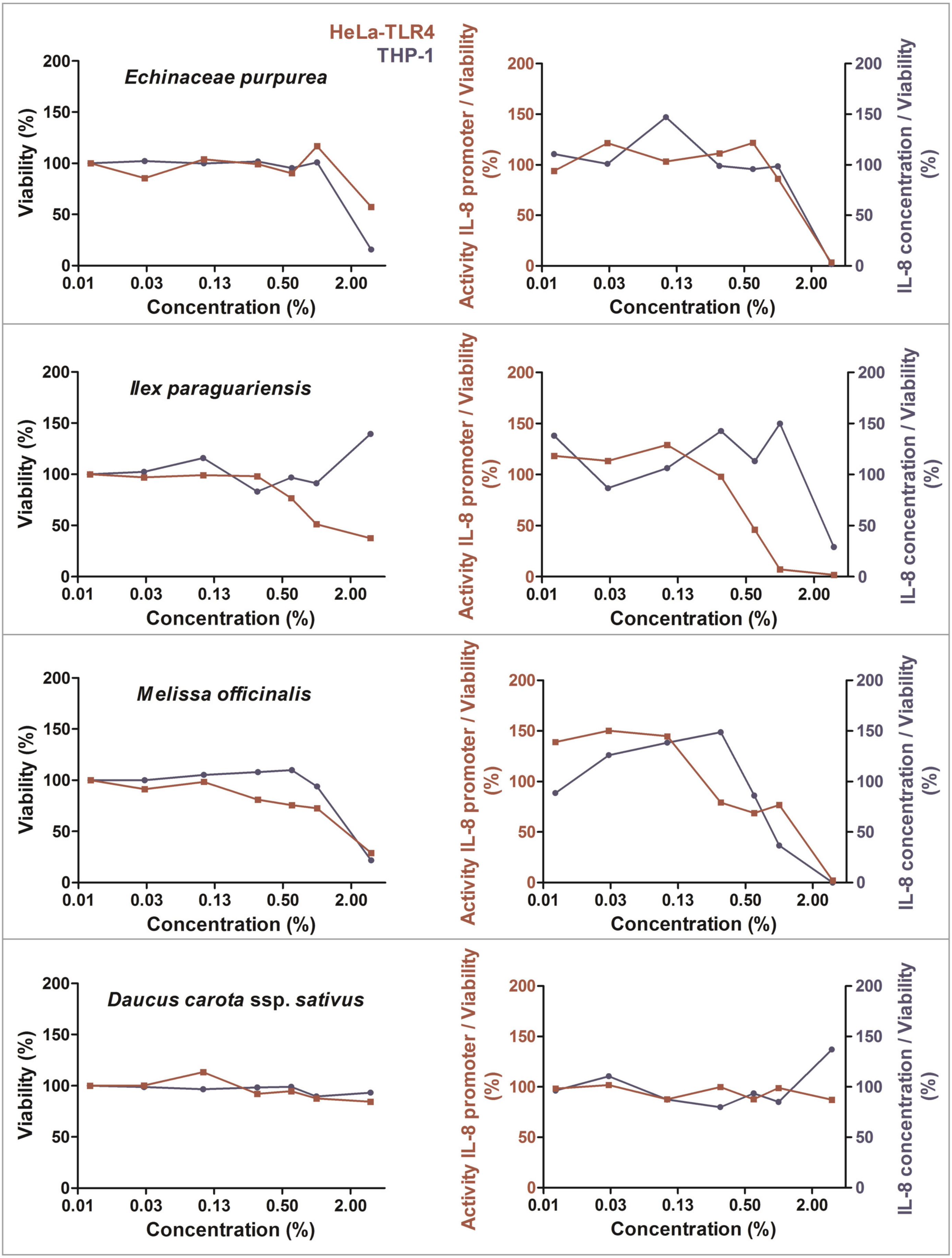
HeLa-TLR4
THP-1
Echinaceae purpurea
Ilex paraguariensis
Melissa officinalis
Daucus carota ssp. sativus
Viability (%)
Activity IL-8 promoter / Viability (%)
IL-8 concentration / Viability (%)
Concentration (%)
0.01
0.03
0.13
0.50
2.00
0
50
100
150
200

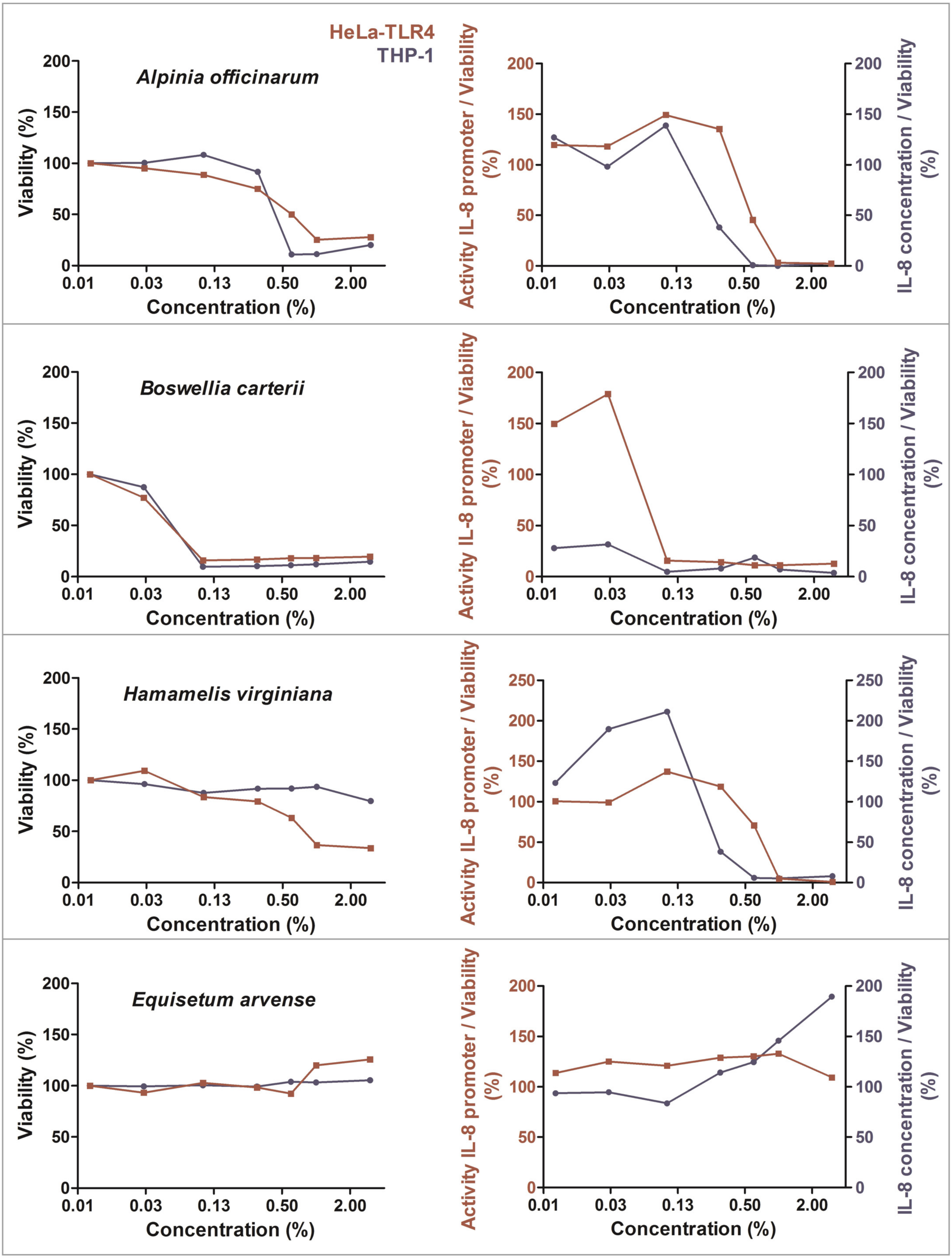
HeLa-TLR4
THP-1
Alpinia officinarum
Boswellia carterii
Hamamelis virginiana
Equisetum arvense
Viability (%)
Concentration (%)
Activity IL-8 promoter / Viability (%)
IL-8 concentration / Viability (%)
0.01
0.03
0.13
0.50
2.00

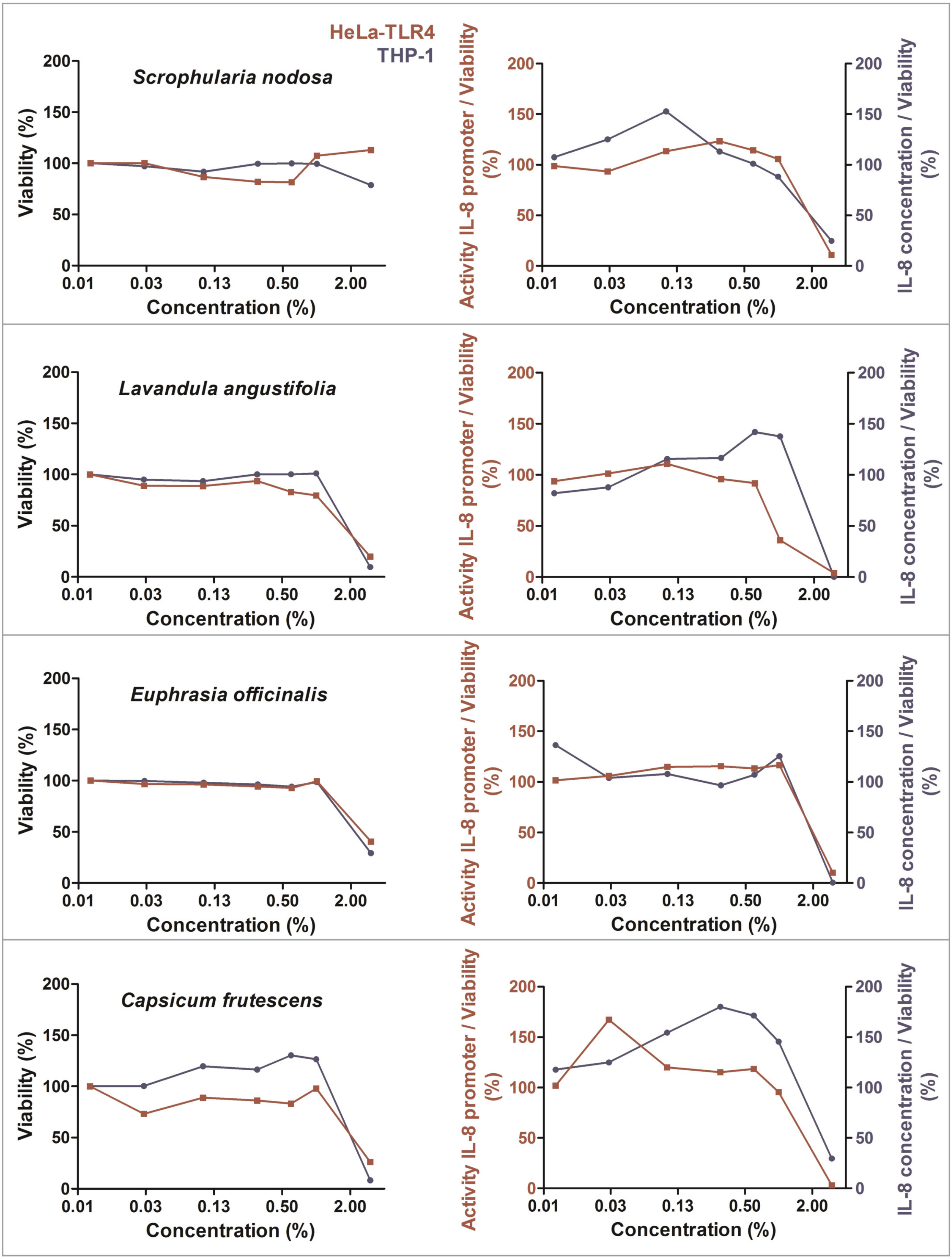
HeLa-TLR4
THP-1
Scrophularia nodosa
Lavandula angustifolia
Euphrasia officinalis
Capsicum frutescens
Viability (%)
Activity IL-8 promoter / Viability (%)
IL-8 concentration / Viability (%)
Concentration (%)
0
50
100
150
200
0.01
0.03
0.13
0.50
2.00

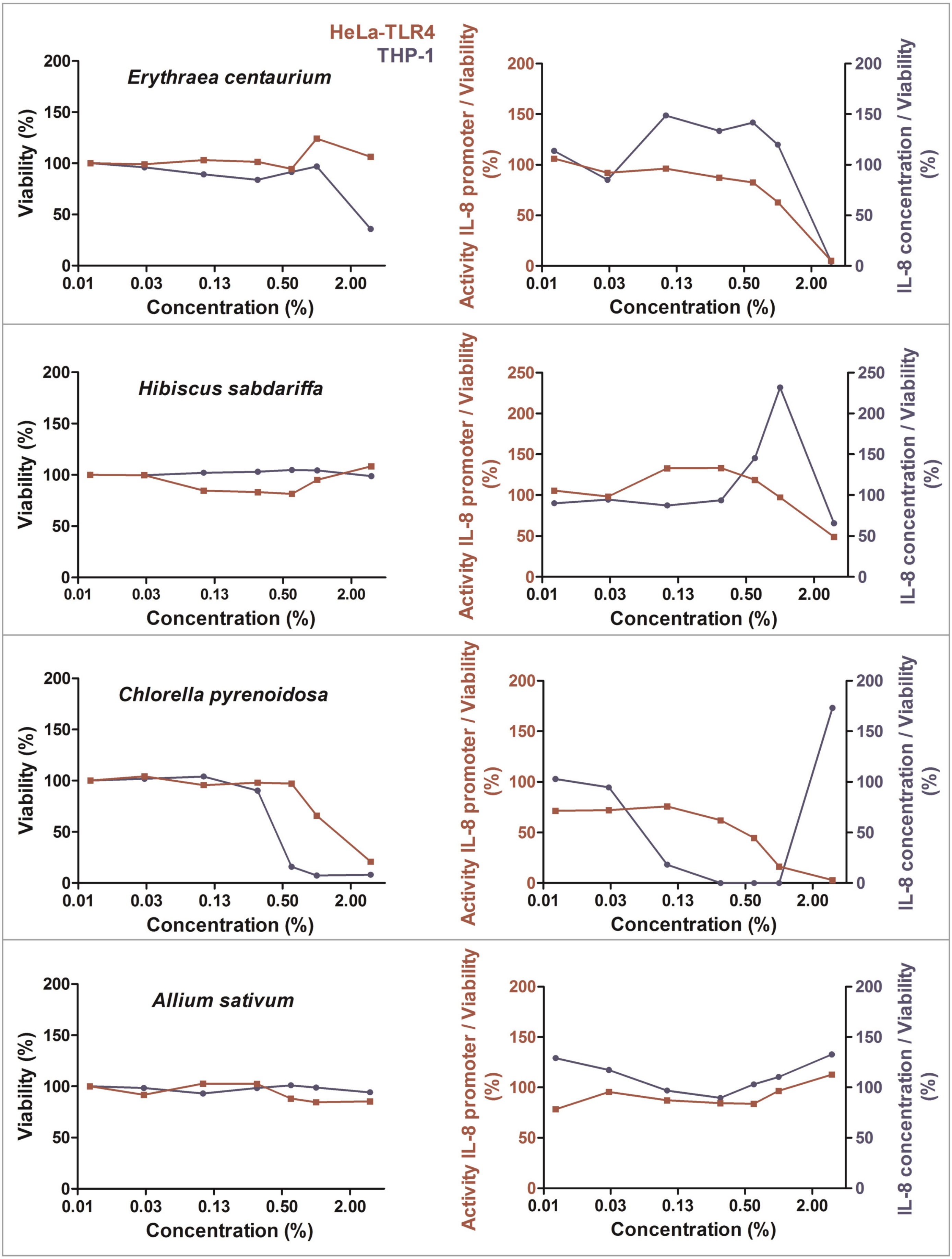
HeLa-TLR4
THP-1
Erythraea centaurium
Hibiscus sabdariffa
Chlorella pyrenoidosa
Allium sativum
Viability (%)
Concentration (%)
Activity IL-8 promoter / Viability (%)
IL-8 concentration / Viability (%)
0.01
0.03
0.13
0.50
2.00
0
50
100
150
200
250

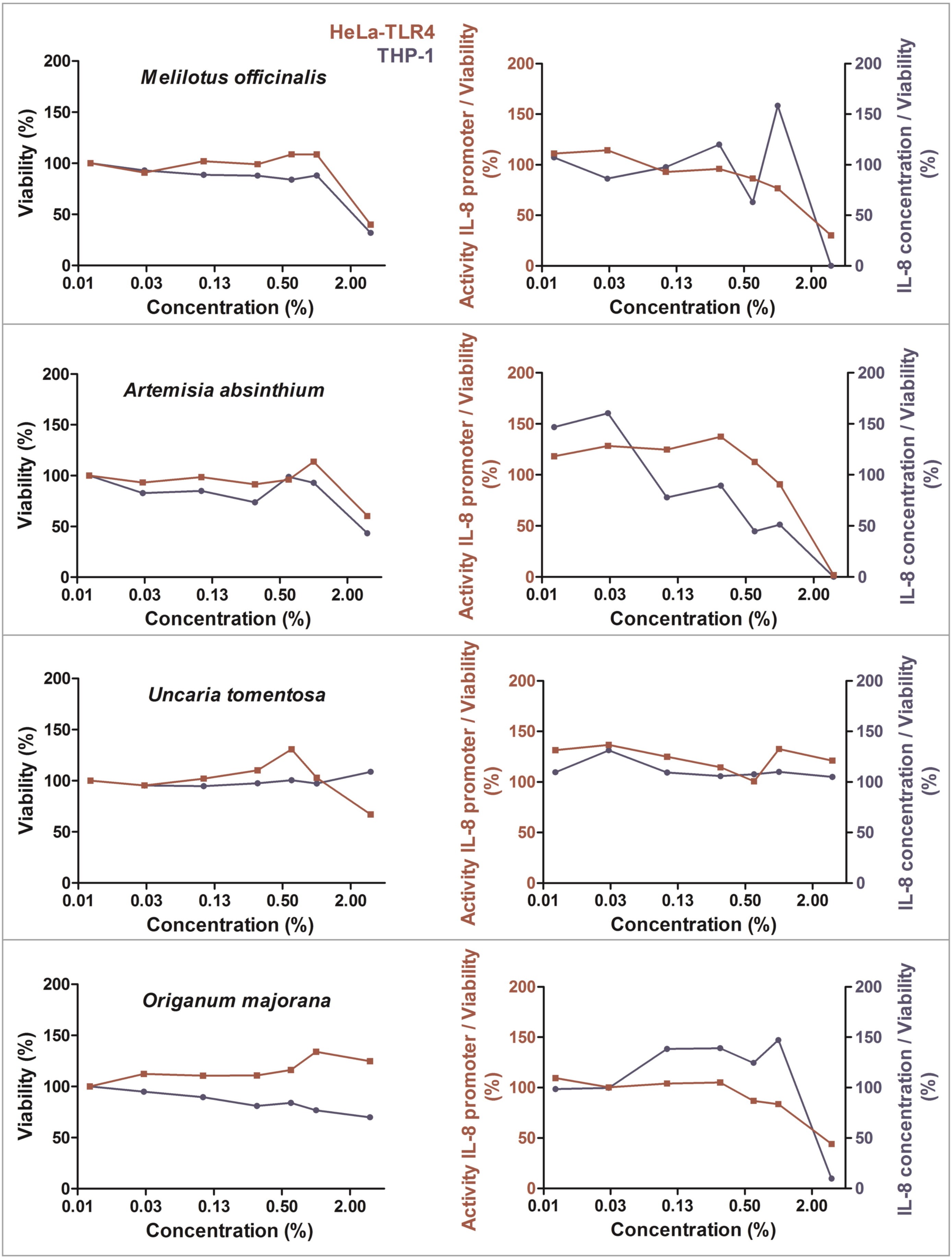
HeLa-TLR4
THP-1
Melilotus officinalis
Artemisia absinthium
Uncaria tomentosa
Origanum majorana
Viability (%)
Activity IL-8 promoter / Viability (%)
IL-8 concentration / Viability (%)
Concentration (%)
0
50
100
150
200
0.01
0.03
0.13
0.50
2.00

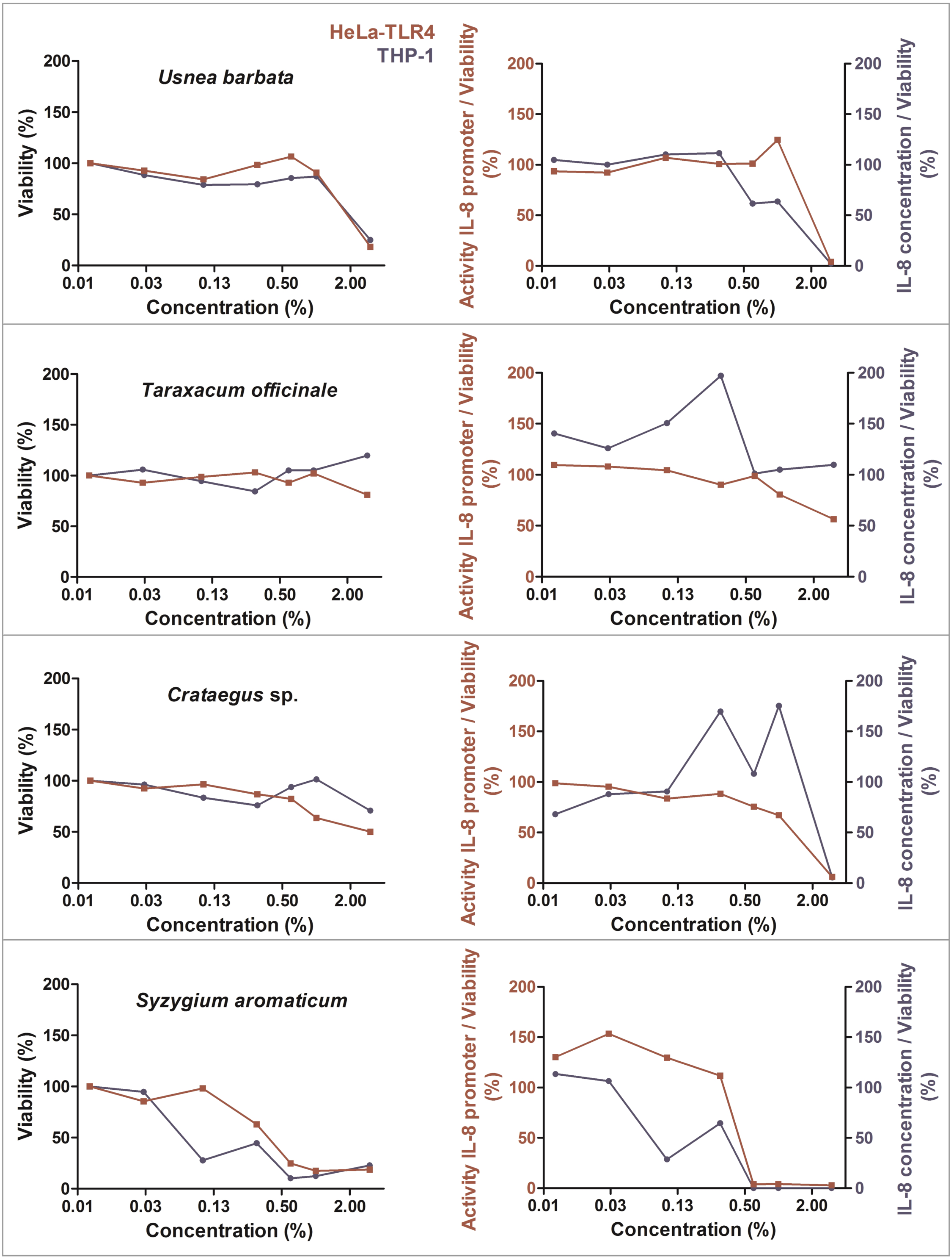
HeLa-TLR4
THP-1
Usnea barbata
Taraxacum officinale
Crataegus sp.
Syzygium aromaticum
Viability (%)
Concentration (%)
Activity IL-8 promoter / Viability (%)
IL-8 concentration / Viability (%)
0
50
100
150
200
0.01
0.03
0.13
0.50
2.00

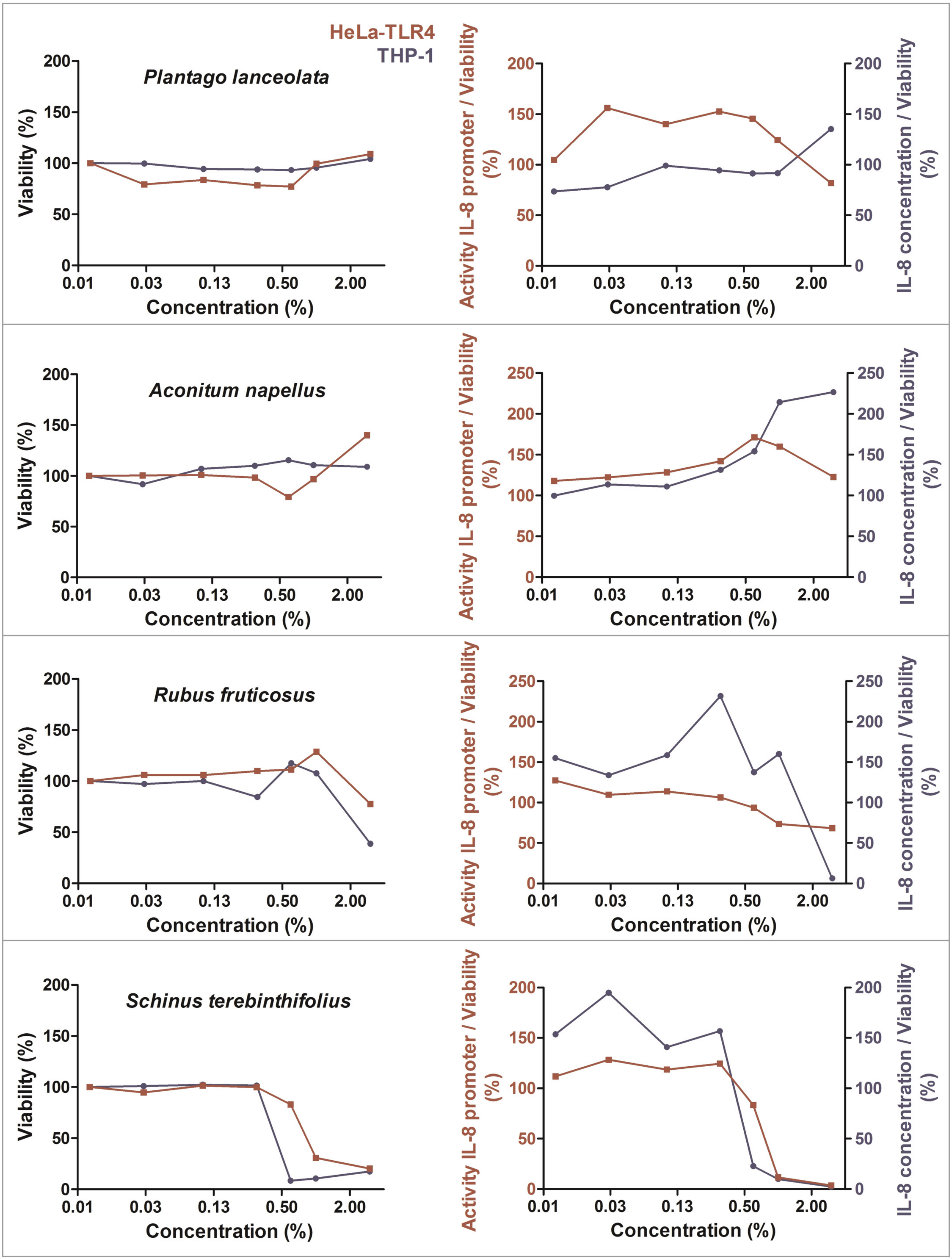
HeLa-TLR4
THP-1
Plantago lanceolata
Aconitum napellus
Rubus fruticosus
Schinus terebinthifolius
Viability (%)
Concentration (%)
Activity IL-8 promoter / Viability (%)
IL-8 concentration / Viability (%)
0.01
0.03
0.13
0.50
2.00

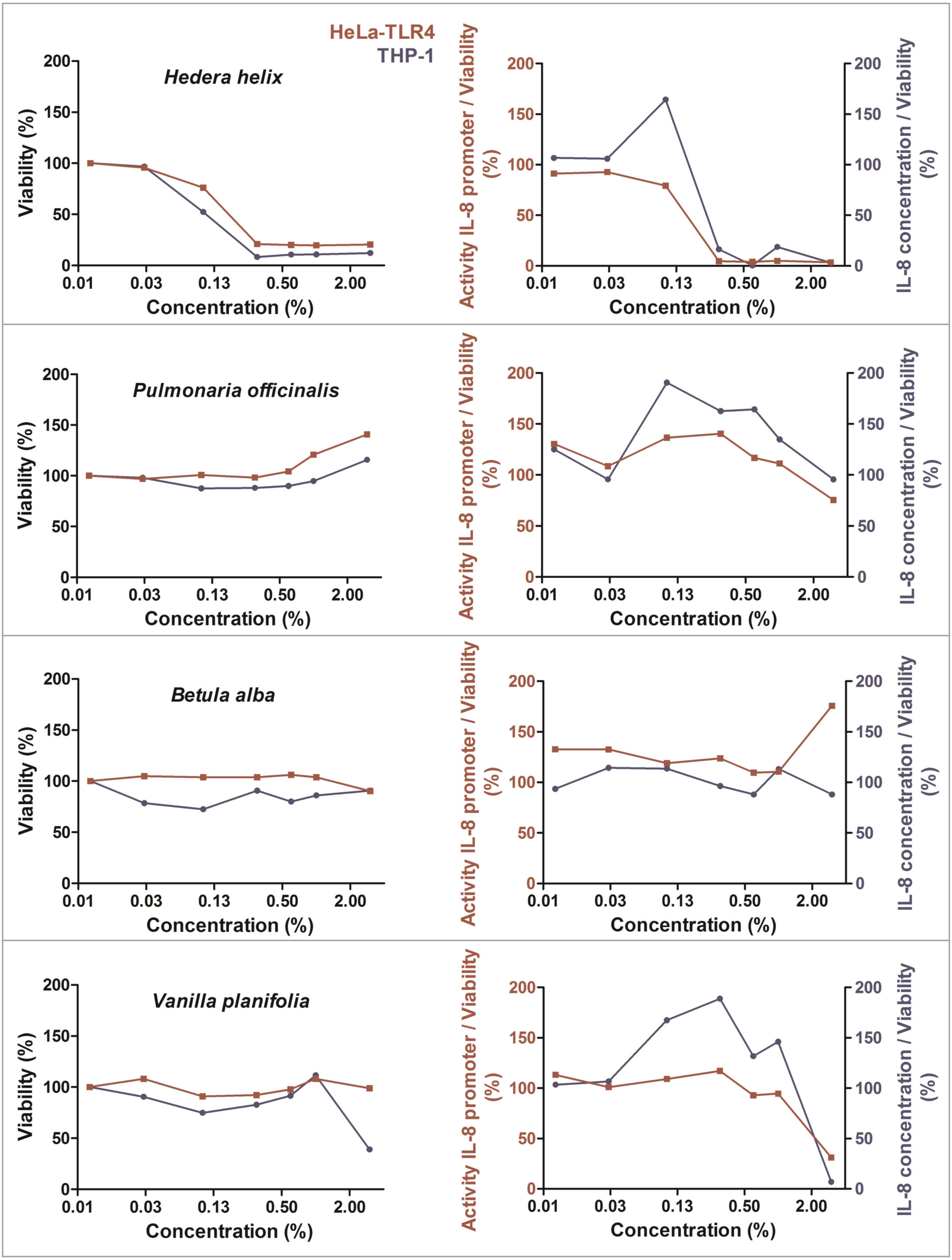
HeLa-TLR4
THP-1
Hedera helix
Pulmonaria officinalis
Betula alba
Vanilla planifolia
Viability (%)
Activity IL-8 promoter / Viability (%)
IL-8 concentration / Viability (%)
Concentration (%)
0.01
0.03
0.13
0.50
2.00
0
50
100
150
200

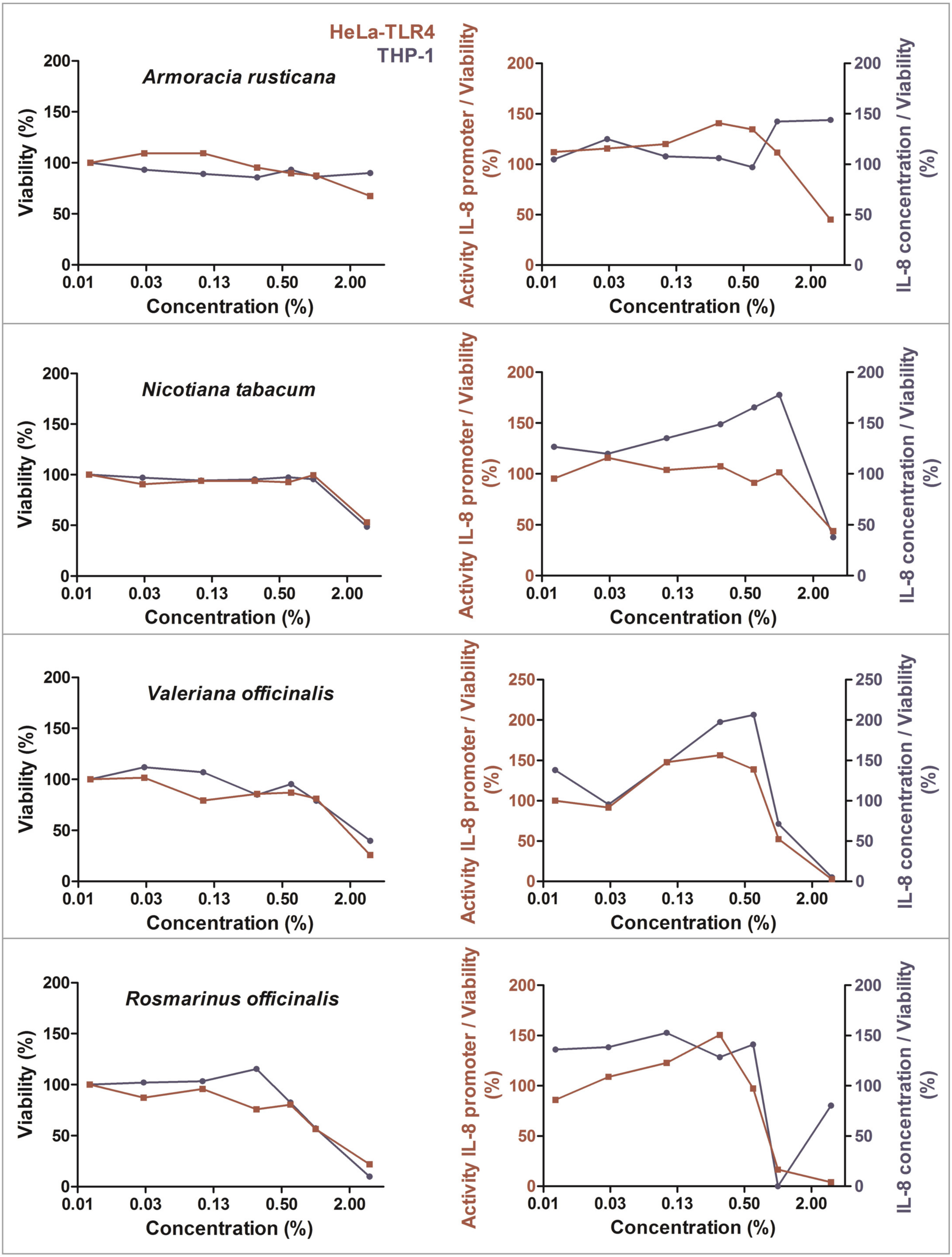
HeLa-TLR4
THP-1
Armoracia rusticana
Nicotiana tabacum
Valeriana officinalis
Rosmarinus officinalis
Viability (%)
Activity IL-8 promoter / Viability (%)
IL-8 concentration / Viability (%)
Concentration (%)
0.01
0.03
0.13
0.50
2.00

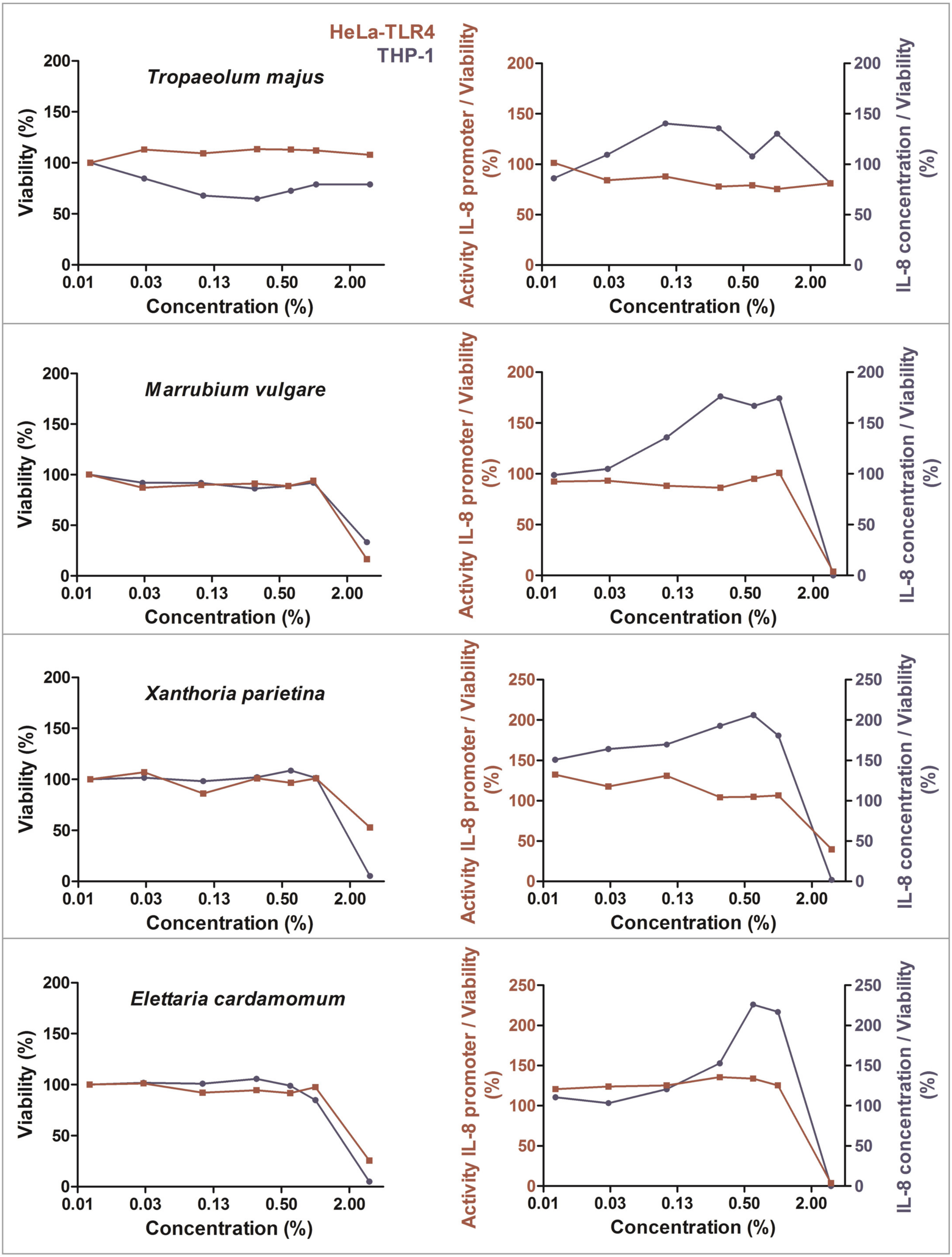

HeLa-TLR4
THP-1
Tropaeolum majus
Marrubium vulgare
Xanthoria parietina
Elettaria cardamomum
Viability (%)
Activity IL-8 promoter / Viability (%)
IL-8 concentration / Viability (%)
Concentration (%)
0.01
0.03
0.13
0.50
2.00
0
50
100
150
200
250

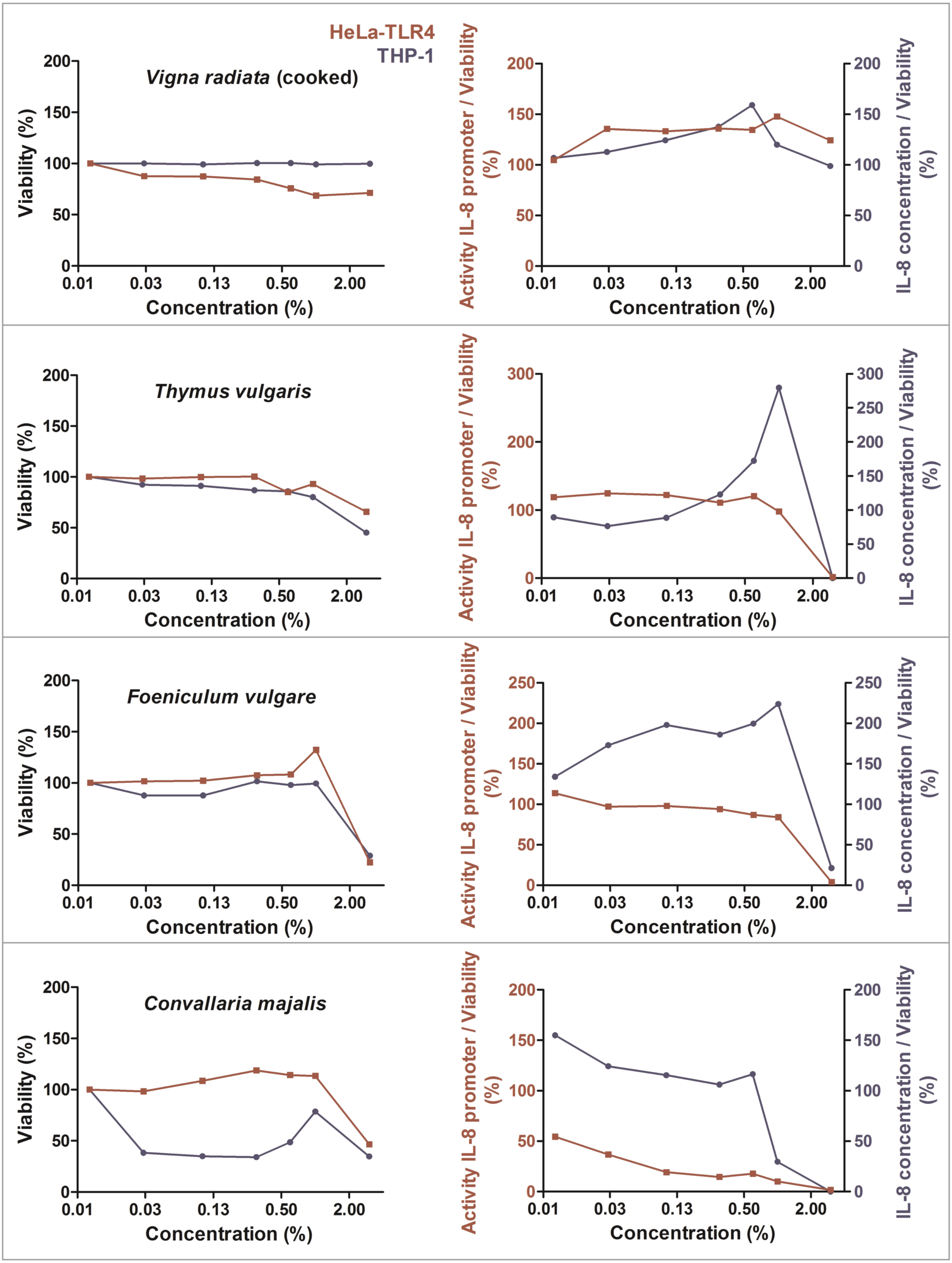
HeLa-TLR4
THP-1
Vigna radiata (cooked)
Thymus vulgaris
Foeniculum vulgare
Convallaria majalis
Viability (%)
Concentration (%)
Activity IL-8 promoter / Viability (%)
IL-8 concentration / Viability (%)
0.01
0.03
0.13
0.50
2.00

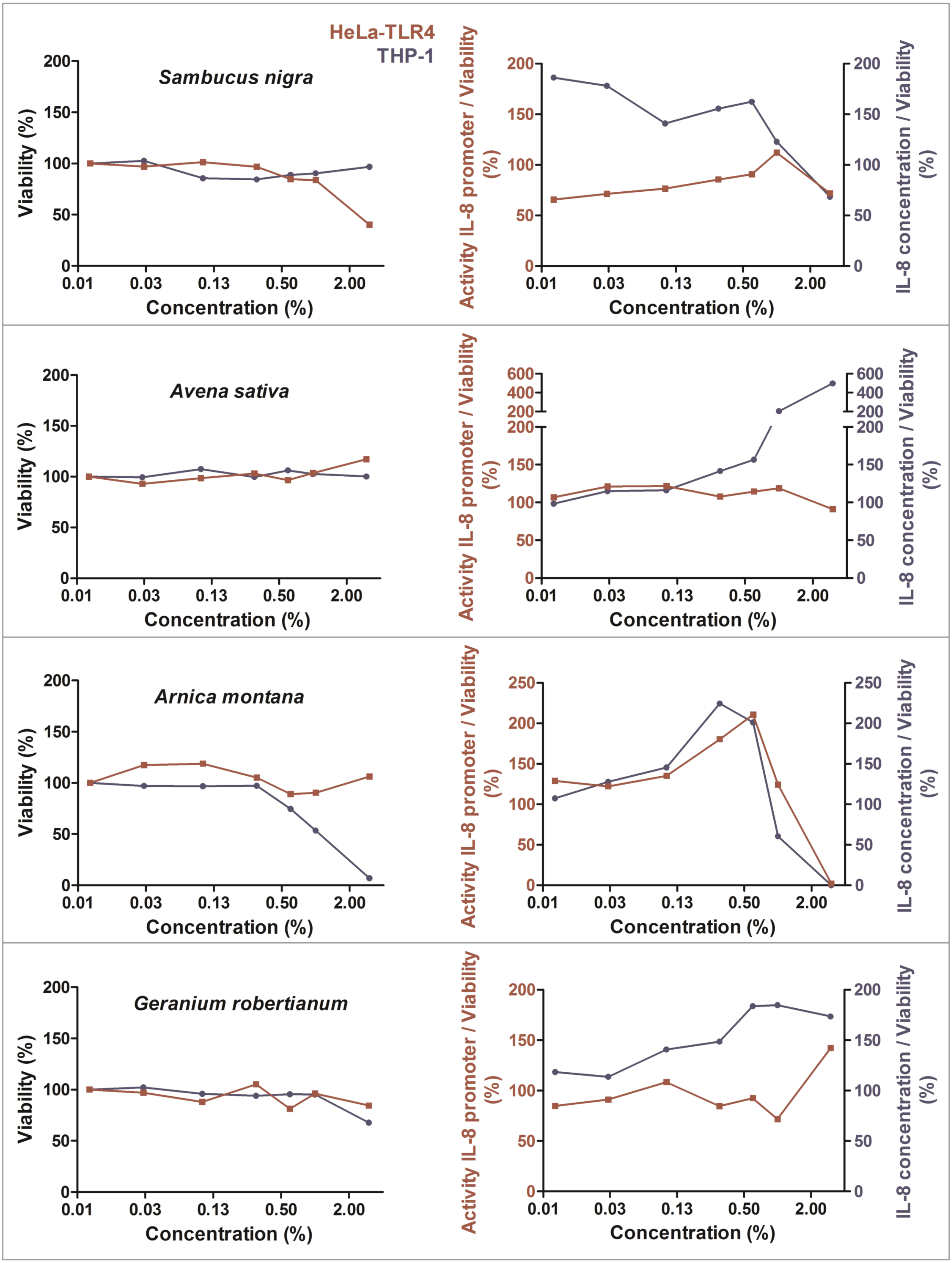
HeLa-TLR4
THP-1
Sambucus nigra
Avena sativa
Arnica montana
Geranium robertianum
Viability (%)
Concentration (%)
Activity IL-8 promoter / Viability (%)
IL-8 concentration / Viability (%)
0.01
0.03
0.13
0.50
2.00

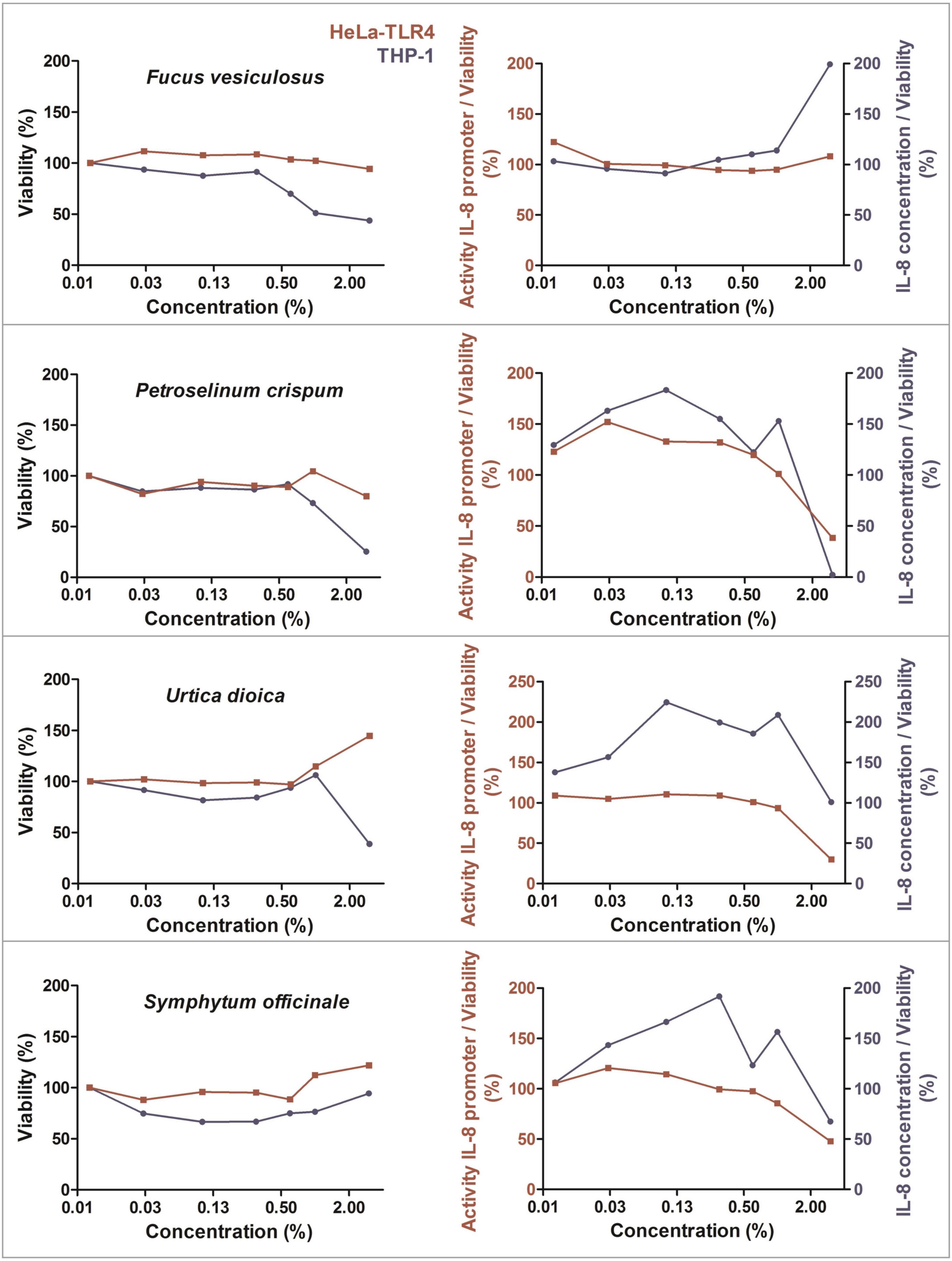

HeLa-TLR4
THP-1
Fucus vesiculosus
Petroselinum crispum
Urtica dioica
Symphytum officinale
Viability (%)
Concentration (%)
Activity IL-8 promoter / Viability (%)
IL-8 concentration / Viability (%)
0.01
0.03
0.13
0.50
2.00
0
50
100
150
200
250

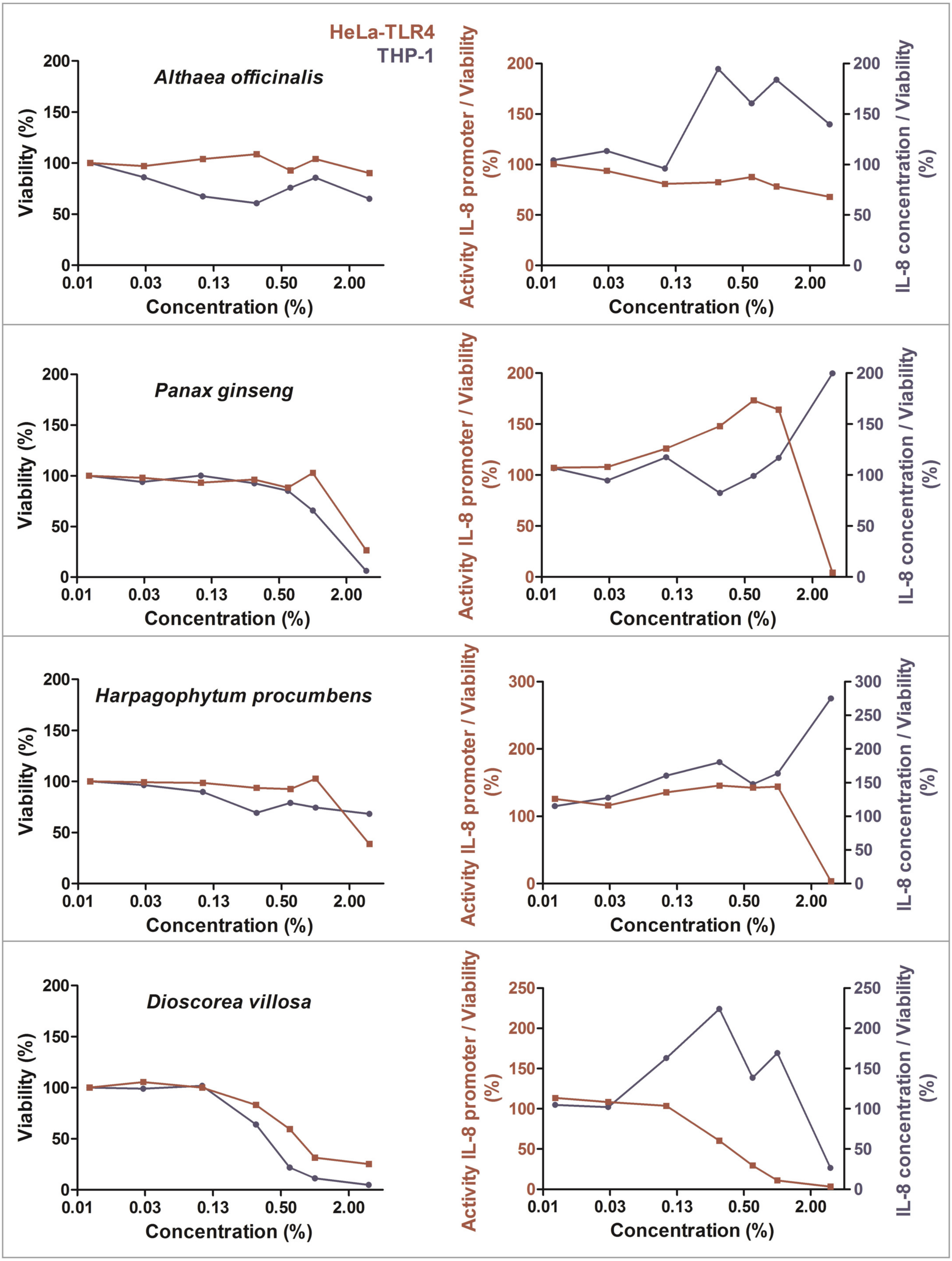

HeLa-TLR4
THP-1
Althaea officinalis
Panax ginseng
Harpagophytum procumbens
Dioscorea villosa
Viability (%)
Activity IL-8 promoter / Viability (%)
IL-8 concentration / Viability (%)
Concentration (%)
0.01
0.03
0.13
0.50
2.00

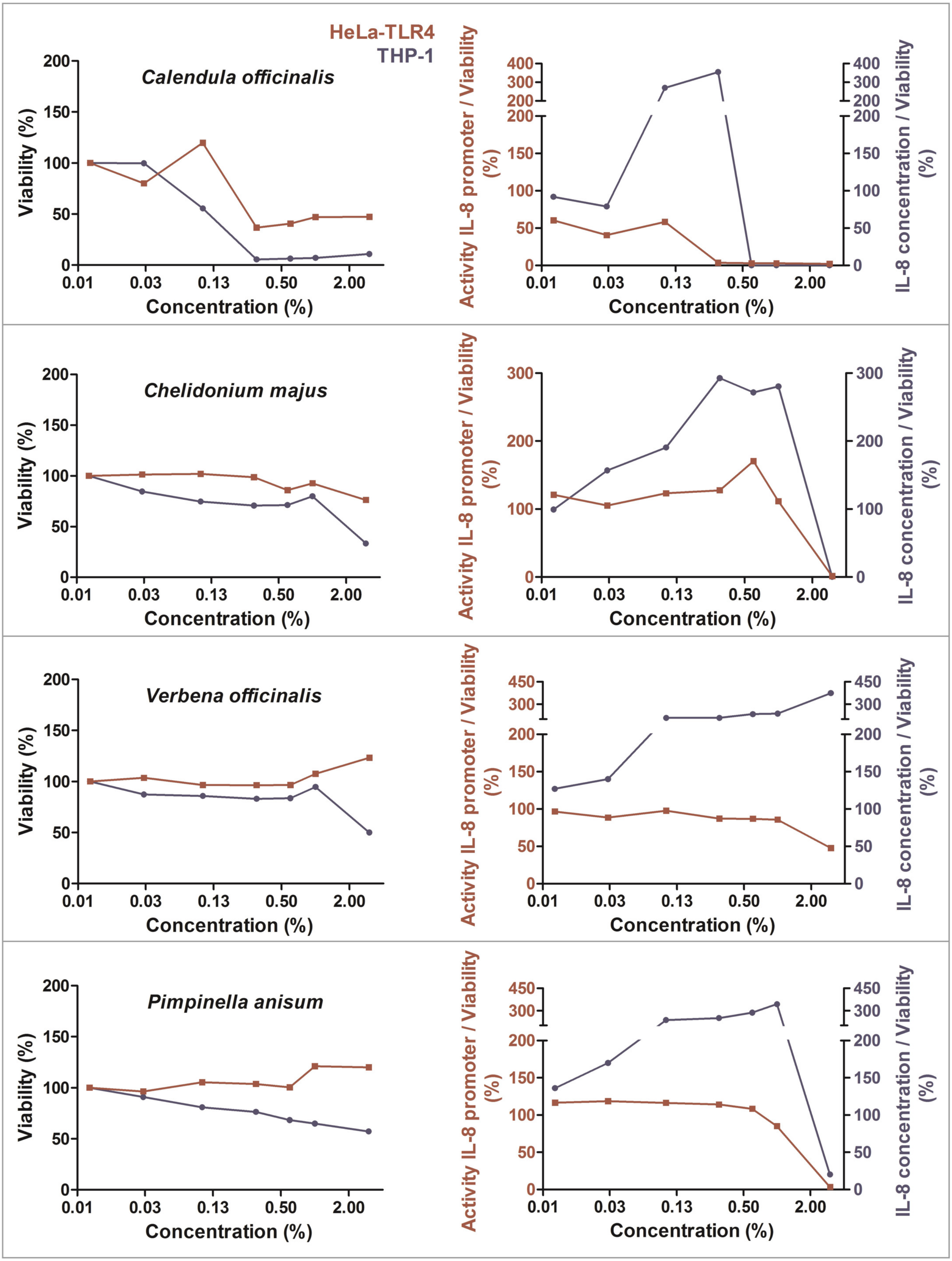
HeLa-TLR4
THP-1
Calendula officinalis
Viability (%)
0
50
100
150
200
0.01
0.03
0.13
0.50
2.00
Concentration (%)
Activity IL-8 promoter / Viability (%)
IL-8 concentration / Viability (%)
0
50
100
150
200
200
300
400
Chelidonium majus
Viability (%)
Concentration (%)
Activity IL-8 promoter / Viability (%)
IL-8 concentration / Viability (%)
0
100
200
300
Verbena officinalis
Viability (%)
Concentration (%)
Activity IL-8 promoter / Viability (%)
IL-8 concentration / Viability (%)
300
450
Pimpinella anisum
Viability (%)
Concentration (%)
Activity IL-8 promoter / Viability (%)
IL-8 concentration / Viability (%)

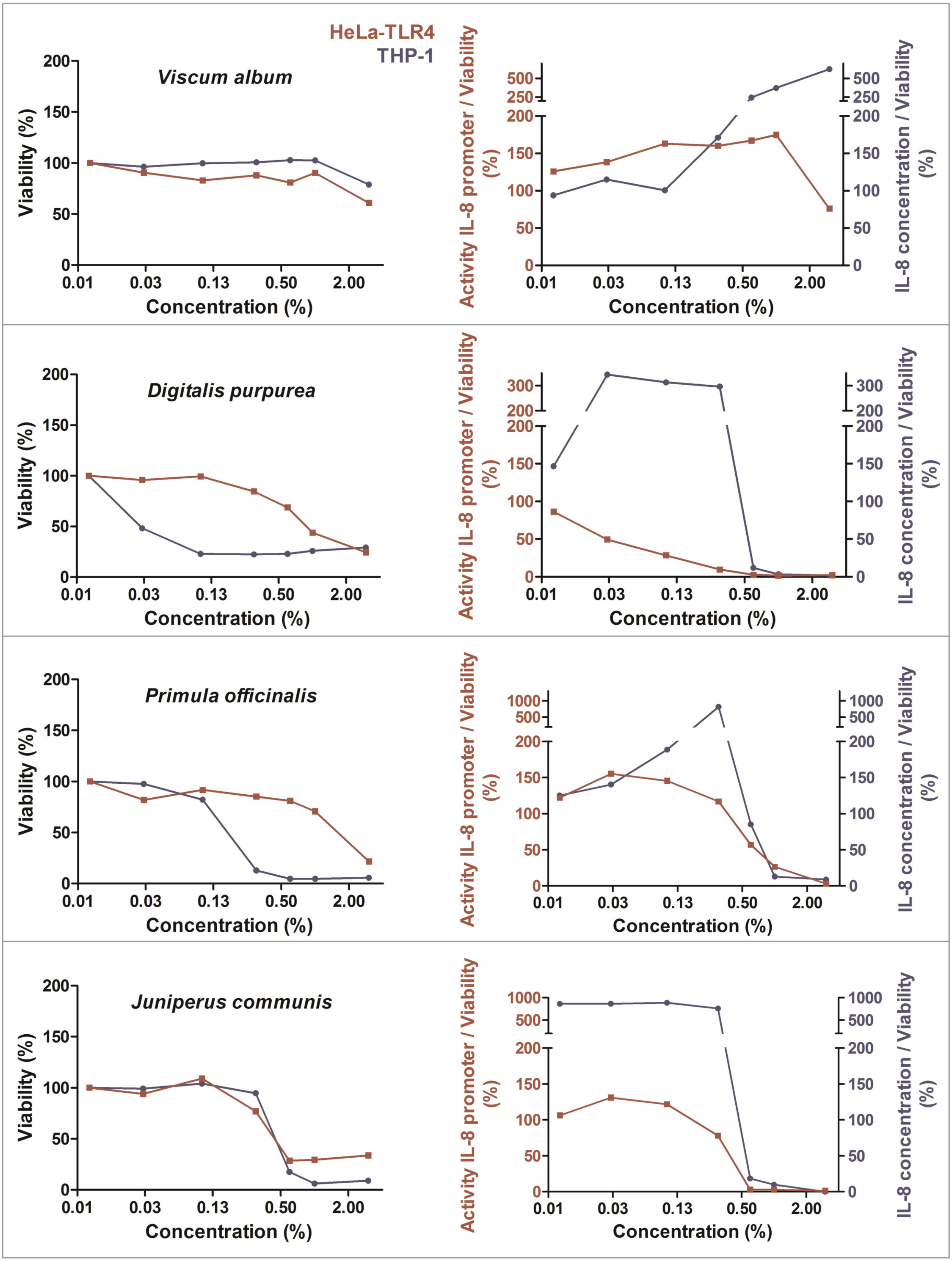

HeLa-TLR4
THP-1
Viscum album
Digitalis purpurea
Primula officinalis
Juniperus communis
Viability (%)
Concentration (%)
Activity IL-8 promoter / Viability (%)
IL-8 concentration / Viability (%)
0.01
0.03
0.13
0.50
2.00

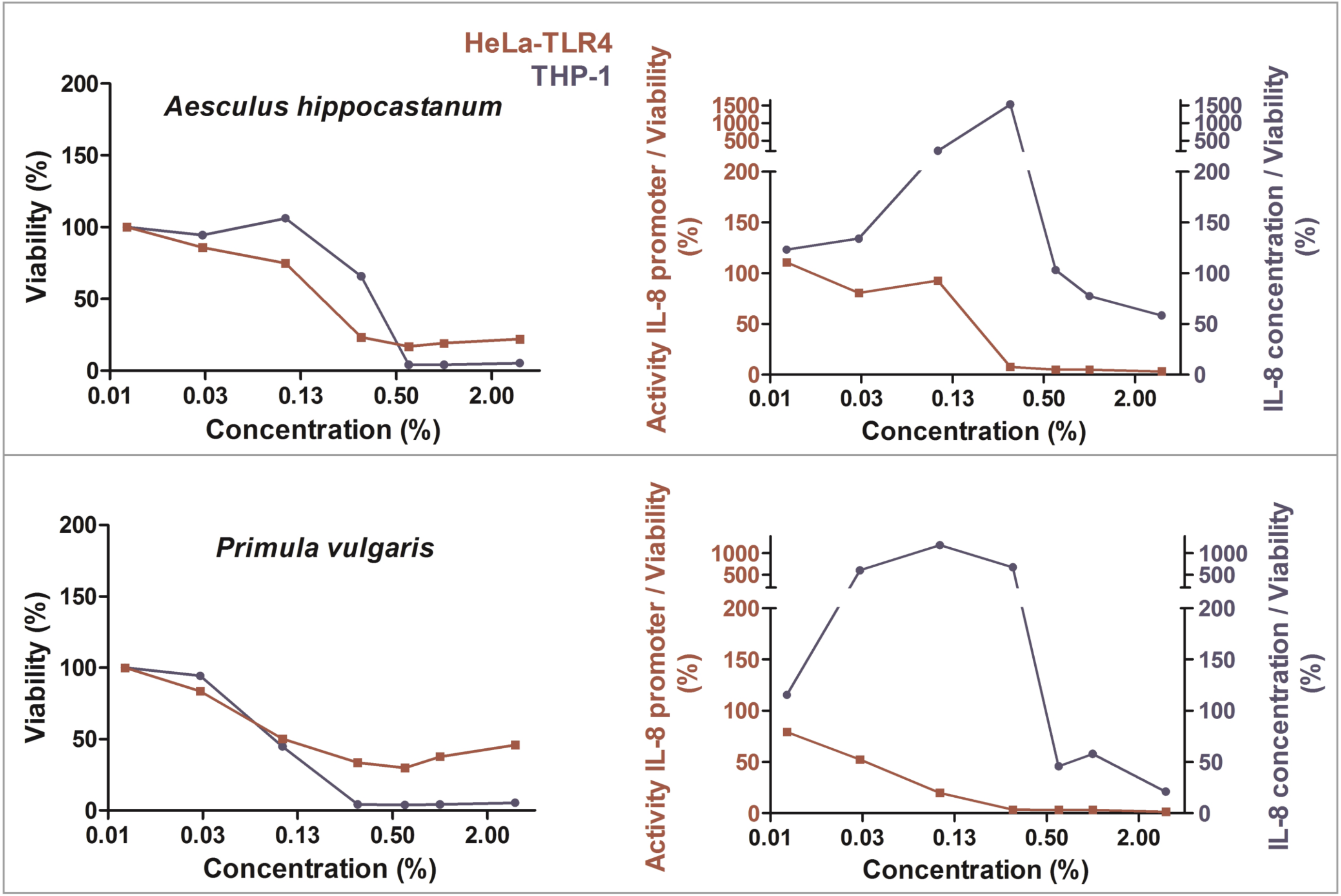
HeLa-TLR4
THP-1
Aesculus hippocastanum
Viability (%)
Concentration (%)
Activity IL-8 promoter / Viability (%)
IL-8 concentration / Viability (%)
Primula vulgaris
0.01
0.03
0.13
0.50
2.00
