# Supplementary material for: Screening of herbal extracts for TLR2- and TLR4-dependent anti-inflammatory effects
Source: PLoS One. 2018 Oct 11;13(10):e0203907. doi: 10.1371/journal.pone.0203907 (PMC6181297; doi:10.1371/journal.pone.0203907)
Supplement: S2 Fig — HeLa-TLR4 reporter cells or THP-1 monocytes were incubated with extracts in different concentrations or vehicle (70% ethanol), followed by stimulation with LPS-EB. Viability was measured using Alamar Blue Assay and was normalized to the negative control (Viability (%)). TLR4 receptor activity was measured using Renilla luciferase expression for the HeLa-TLR4 cell line or IL-8 ELISA (pg/ml) for THP-1 monocytes and was normalized to ethanol-treated cells (TLR4-Activity). Data are displayed as TLR4 stimulation divided by viability and sorted ascending by the following formula: (150—Viability (%)) * (2 * TLR4-Activity + 100) weighted in a ratio of 2:1 for THP-1 monocytes vs. HeLa-TLR4 cells. Data represents means (n≥2). (PDF) [file pone.0203907.s003.pdf]

Inflammatory scale

anti-inflammatory — pro-inflammatory

Toxicity scale

viability < 75%

Latin name	Common English name	Used part	HeLa-TLR4 reporter cell line 0.01%	0.03%	0.1%	0.3%	0.6%	1%	3%	THP-1 monocytes 0.01%	0.03%	0.1%	0.3%	0.6%	1%	3%
Ethanol control			100,00	101,30	103,67	102,01	93,23	85,77	90,26	98,88	97,04	96,54	96,59	97,67	95,66	107,91
Castanea sativa	Sweet chestnut	Leaf	90,53	82,03	30,91	3,53	10,59	1,34	0,79	118,69	71,34	46,42	25,73	5,65	0,00	0,00
Cinchona pubescens	Cinchona	Bark	122,15	137,25	148,02	131,28	148,76	98,71	2,77	47,68	104,26	125,10	62,37	29,49	15,68	3,49
Cinnamomum verum	Cinnamon	Bark	96,77	98,57	109,17	83,50	59,41	21,59	3,24	101,16	87,70	95,31	106,02	26,08	6,08	11,36
Salix alba	White willow	Bark	93,03	74,48	79,05	99,83	58,02	3,79	1,45	97,50	93,93	88,45	33,44	5,95	0,00	21,34
Rheum palmatum	Rhubarb	Root	71,38	72,58	79,60	55,28	64,48	96,37	2,34	99,17	98,60	27,86	6,42	1,94	3,57	29,89
Alchemilla vulgaris	Common lady's mantle	Whole plant	91,35	85,79	82,86	76,83	66,53	78,18	1,63	125,37	102,96	92,63	62,93	60,89	73,46	0,00
Humulus lupulus	Hops	Flower	64,31	67,38	74,08	3,57	1,10	0,92	0,96	108,76	99,78	55,66	1,46	0,00	0,00	0,73
Vaccinium myrtillus	Bilberries	Fruit/berry/seed	86,00	84,71	81,50	80,48	83,01	76,30	68,10	102,84	91,37	92,22	96,85	92,91	92,95	63,02
Curcuma longa	Turmeric	Root	124,40	109,62	142,43	67,28	11,92	5,54	1,83	127,14	110,90	115,73	45,39	8,99	0,00	15,80
Arctostaphylos uva-ursi	Bearberry	Leaf	91,28	109,88	127,59	123,26	86,52	40,81	1,18	89,45	99,97	27,43	5,97	1,72	4,71	35,80
Allium ursinum	Wild garlic	Leaf	115,74	118,86	97,71	91,94	81,27	78,25	42,93	105,80	109,20	77,64	38,94	24,16	5,90	11,21
Hypericum perforatum	St John's wort	Whole plant	120,77	104,44	85,91	83,69	71,41	53,35	14,06	91,44	85,40	90,32	71,54	51,19	32,03	1,14
Arnicae montana	Arnica	Flower	114,17	96,34	2,78	2,14	2,61	2,55	1,28	144,96	146,96	11,90	0,70	0,69	1,67	0,53
Aloe ferox	Aloe	Whole plant	105,30	105,90	82,53	58,19	27,69	5,15	1,30	123,82	117,87	88,51	38,22	9,71	7,37	0,13
Cynara scolymus	Artichoke	Leaf	109,98	112,42	117,52	100,14	90,16	31,21	1,99	47,40	53,68	127,22	228,92	16,56	6,13	2,86
Salvia officinalis	Salvia	Leaf	94,88	96,95	107,50	89,57	73,14	17,55	2,65	94,87	87,05	79,10	72,53	97,48	42,03	0,00
Ginkgo biloba	Ginkgo	Leaf	107,04	120,25	119,37	90,15	44,17	4,77	2,22	124,31	101,09	22,26	7,98	16,68	9,27	0,00
Tanacetum parthenium	Feverfew	Whole plant	127,34	111,53	120,04	114,28	99,98	68,82	2,22	129,88	139,58	123,43	124,51	72,56	21,24	6,12
Vigna radiata	Mung bean (dried)	Fruit/berry/seed	83,31	85,02	87,84	85,99	69,53	52,04	25,33	121,14	141,32	120,83	62,61	38,87	23,18	11,00
Betula verrucosa	Weeping birch	Juice/resin	144,10	148,90	136,35	139,46	143,77	154,99	88,24	120,90	131,27	106,29	88,92	93,49	94,35	93,18
Filipendula ulmaria	Meadowsweet	Flower	120,04	113,11	130,06	117,06	106,72	131,55	52,01	132,40	135,84	124,42	75,12	23,05	8,87	1,88
Matricaria chamomilla	Chamomile	Whole plant	100,35	104,50	90,84	78,36	84,92	5,43	1,26	107,39	110,30	81,01	78,37	109,61	0,00	0,00
Spirulina	Spirulina	Whole cyanobacteria	93,17	100,77	107,05	92,24	76,80	50,64	3,57	142,68	138,71	169,61	74,85	20,59	2,70	36,20
Gentiana lutea	Gentian	Root	120,08	105,07	118,69	89,81	89,96	82,85	39,06	80,63	60,91	89,51	140,23	62,51	43,53	2,92
Quercus robur	English oak	Bark	143,68	142,56	127,88	124,88	134,53	106,17	8,76	106,31	116,82	88,62	57,00	35,67	16,07	16,81
Glycyrrhiza glabra	Liquorice	Root	69,76	13,55	17,52	2,60	2,55	2,39	1,41	124,15	119,86	9,19	2,26	0,00	0,00	0,00
Coriandrum sativum	Coriander	Fruit/berry/seed	94,70	82,24	87,66	90,37	89,65	77,49	44,98	145,74	168,38	152,38	158,20	188,96	155,51	6,45
Achillea millefolium	Common yarrow	Whole plant	99,04	103,48	102,37	83,36	68,35	55,50	4,09	93,70	98,28	118,44	107,59	100,57	52,47	0,54
Mentha piperita	Peppermint	Whole plant	102,97	91,41	91,14	78,20	72,36	55,95	6,57	130,45	151,24	119,30	123,66	149,47	84,49	0,00
Zingiber officinale	Ginger	Root	94,65	103,21	52,82	24,16	2,18	4,32	3,92	73,81	82,12	75,13	80,11	34,29	0,19	6,73
Carum carvi	Caraway	Fruit/berry/seed	108,52	107,56	121,15	115,23	96,15	83,63	2,63	126,27	123,61	127,15	136,91	201,67	118,87	0,00
Boswellia serrata	Frankincense	Juice/resin	109,56	96,96	80,61	3,87	2,80	3,49	2,35	97,42	88,36	75,32	23,64	10,16	11,86	9,76
Camellia sinensis (L.)	Green tea	Leaf	71,42	64,90	60,73	53,03	34,40	5,10	2,05	104,85	90,19	80,66	52,63	128,30	78,12	71,24
Echinacea purpurea	Purple coneflower	Whole plant	93,87	121,50	103,17	111,27	121,75	86,28	3,42	110,63	100,92	147,05	98,88	95,70	98,35	1,71
Ilex paraguariensis	Yerba mate	Leaf	118,38	113,40	129,00	98,03	46,11	7,19	1,87	138,05	86,82	106,45	142,70	113,19	149,98	29,15
Melissa officinalis	Lemon balm	Leaf	139,03	150,28	144,79	79,25	68,78	76,74	2,31	88,63	126,13	138,48	148,82	86,05	36,73	0,00
Daucus carota ssp. sativus	Carrot	Root	98,38	101,96	87,71	99,88	87,68	98,85	87,26	96,35	110,50	87,33	79,92	93,51	85,16	137,06
Alpinia officinarum	Galangal	Root	119,58	118,04	149,25	135,19	45,52	3,29	2,27	127,08	98,10	138,77	38,12	0,70	0,00	2,44
Boswellia carterii	Frankincense	Whole plant	149,54	178,92	15,63	14,15	11,16	11,09	12,66	27,95	31,63	4,81	7,98	18,51	6,92	3,63
Hamamelis virginiana	Witch hazel	Leaf	100,46	99,15	137,19	118,74	70,76	4,96	1,01	123,19	189,63	211,05	38,28	5,85	5,40	7,92
Equisetum arvense	Field horsetail	Whole plant	113,79	125,14	120,99	128,87	130,20	133,00	109,17	93,55	94,65	83,58	114,07	124,64	145,69	189,41

Scrophularia nodosa	Common figwort	Whole plant	98,78	93,42	113,41	123,22	114,38	105,67	10,92	107,47	125,14	152,78	113,07	101,17	88,26	24,69
Lavandula angustifolia	Lavender	Flower	93,68	101,24	110,76	95,94	91,92	36,15	3,80	81,97	87,67	115,57	116,55	141,91	137,61	0,00
Euphrasia officinalis	Eyebright	Whole plant	101,53	105,92	114,90	115,55	113,31	116,53	10,37	136,41	103,87	107,91	96,64	107,09	125,33	0,38
Capscicum frutescens	Chili	Fruit/berry/ seed	101,94	167,29	120,07	115,20	118,62	95,65	3,07	117,80	124,86	154,50	180,15	171,38	145,55	29,51
Erythraea centaurium	Common centaury	Whole plant	106,04	92,14	96,31	87,33	82,62	62,99	5,13	113,71	85,01	148,74	133,41	141,78	119,84	4,22
Hibiscus sabdariffa	Roselle	Leaf	105,58	98,26	132,94	133,23	118,60	97,39	48,78	90,10	94,55	87,49	93,64	145,26	231,97	65,51
Chlorella pyrenoidosa	Chlorella	Whole green algae	71,44	72,14	75,70	62,00	44,51	16,46	2,86	102,98	94,60	18,27	0,00	0,00	0,00	173,01
Allium sativum	Garlic	Root	78,39	95,63	87,21	84,35	83,82	96,56	112,72	129,06	117,29	96,92	89,62	103,10	110,42	132,77
Melilotus officinalis	Sweet clover	Whole plant	111,02	114,31	92,87	95,90	86,37	76,52	30,05	107,23	86,20	97,54	119,85	62,88	158,37	0,00
Artemisia absinthium	Wormwood	Whole plant	118,20	128,44	124,74	137,46	112,64	90,75	1,69	146,74	160,40	77,99	89,46	44,77	51,21	0,00
Uncaria tomenosa	Cat's claw	Whole plant	131,44	136,66	124,86	114,39	100,57	132,50	121,12	109,66	131,32	109,33	105,76	107,51	109,99	104,97
Origanum majorana	Marjoram	Whole plant	109,40	100,22	104,08	105,12	87,00	83,56	44,13	98,64	99,81	138,44	139,16	124,52	147,26	9,77
Usnea barbata	Barber's itch	Whole plant	93,46	92,28	106,88	100,70	101,15	124,63	3,85	104,78	100,16	110,27	111,35	61,61	63,62	2,27
Taraxacum officinale	Dandelion	Whole plant	109,52	108,09	104,38	90,20	98,79	80,62	56,45	140,46	125,98	150,64	197,11	101,07	104,89	109,75
Crataegus sp.	Hawthorn	Fruit/berry/ seed	98,69	95,22	83,59	88,20	75,56	67,10	6,03	68,02	87,86	90,53	169,63	108,10	175,25	5,53
Syzygium aromaticum	Clove	Flower	130,27	153,49	129,52	111,74	3,84	4,04	2,97	113,40	106,43	28,55	64,60	0,00	0,00	0,00
Plantago lanceolata	Ribwort	Whole plant	104,81	156,04	140,16	152,61	145,60	124,31	81,91	73,59	77,75	99,03	94,45	91,42	91,59	135,20
Aconitum napellus	Monkshood	Whole plant	117,73	122,26	128,16	141,94	171,12	159,82	122,60	99,75	113,53	110,99	131,43	154,05	214,24	226,55
Rubus fruticosus	Blackberry	Leaf	127,50	109,58	113,80	106,40	93,56	73,60	68,53	155,10	133,75	158,55	231,87	137,62	160,15	6,26
Schinus terebinthifolius	Brazilian peppertree	Fruit/berry/ seed	111,76	128,25	118,65	124,42	83,27	11,51	3,58	153,65	194,72	140,78	156,82	22,67	9,93	2,35
Hedera helix	Common ivy	Leaf	91,23	92,82	79,19	4,54	3,93	4,92	3,35	106,81	105,99	164,47	16,45	0,48	18,81	3,05
Pulmonaria officinalis	Common lungwort	Flower	130,49	108,61	136,54	140,36	116,71	111,27	75,45	125,06	95,78	190,61	162,64	164,20	134,71	95,52
Betula alba	Birch	Juice/resin	132,70	132,59	119,10	123,74	109,52	110,59	175,69	93,66	114,38	113,57	96,37	88,04	113,21	88,16
Vanilla planifolia	Vanilla	Fruit/berry/ seed	113,32	101,03	109,01	117,05	92,96	94,61	31,02	103,36	106,83	167,48	188,94	131,81	146,05	6,91
Armoracia rusticana	Horseradish	Root	112,02	115,66	120,07	140,73	134,50	111,66	45,08	104,74	124,98	107,81	106,12	97,03	142,36	143,87
Nicotiana tabacum	Tobacco	Leaf	95,45	115,94	103,93	107,47	91,20	101,64	43,82	126,54	119,78	135,07	148,77	165,43	177,65	37,75
Valeriana officinalis (L.)	Common valerian	Root	100,20	91,61	147,82	156,35	138,95	52,36	2,58	138,12	95,56	147,35	197,61	206,61	71,26	5,16
Rosmarinus officinalis	Rosemary	Leaf	85,92	108,99	122,78	150,67	97,28	16,54	4,02	136,08	138,36	152,73	128,42	141,11	0,15	80,17
Tropaeolum majus	Nasturtium	Whole plant	101,46	84,01	87,90	77,92	79,16	75,39	81,14	86,16	109,46	140,46	135,69	107,73	130,22	81,44
Marrubium vulgare	Common horehound	Whole plant	92,34	93,29	88,32	86,32	95,10	100,87	3,75	98,80	104,74	135,83	176,09	166,83	174,18	0,00
Xanthoria parietina	Common orange lichen	Whole lichen	132,43	117,59	130,84	104,27	104,89	106,46	39,67	150,70	163,97	169,59	192,87	206,05	180,74	1,76
Elettaria cardamomum	Cardamom	Fruit/berry/ seed	120,81	123,83	125,38	135,62	133,76	125,41	3,62	110,45	103,25	120,45	152,87	225,96	216,74	0,00
Vigna radiata	Mung bean (cooked)	Fruit/berry/ seed	104,69	135,60	133,28	135,94	134,61	147,80	124,20	106,85	112,74	124,20	137,77	159,06	119,88	98,96
Thymus vulgaris	Common thyme	Whole plant	118,89	124,53	122,18	110,86	120,29	97,87	1,59	89,27	76,48	88,74	122,81	172,45	279,61	0,00
Foeniculum vulgare	Fennel	Fruit/berry/ seed	113,92	97,22	97,97	94,07	87,06	84,14	3,97	134,14	173,01	197,93	186,12	199,79	223,76	21,35
Convallaria majalis	Lily of the valley	Whole plant	54,48	36,78	19,24	14,57	17,70	10,11	1,72	154,97	124,30	115,50	106,13	116,39	29,53	0,00
Sambucus nigra (L.)	Elderflower	Flower	65,76	71,40	76,66	85,55	90,78	112,07	71,97	186,27	178,06	140,90	155,66	162,33	122,89	68,63
Avena sativa	Oat	Whole plant	106,83	121,15	121,84	107,76	114,50	118,79	91,19	98,22	114,88	116,09	141,54	156,55	204,12	498,22
Arnica montana	Arnica	Whole plant	128,77	122,08	135,39	180,41	210,78	124,21	2,17	107,35	127,87	145,42	224,38	201,18	60,44	0,00
Geranium robertianum	Herb Robert	Whole plant	84,73	91,07	108,37	84,64	92,55	71,75	142,28	118,42	113,83	140,82	148,56	183,75	184,72	173,60
Fucus vesiculosus	Bladderwrack	Whole plant	122,31	100,62	99,24	94,51	93,75	94,92	108,01	103,21	95,74	91,21	104,75	110,16	113,90	199,19
Petroselinum crispum	Parsley	Whole plant	122,97	152,03	133,00	132,12	119,74	101,03	38,49	129,60	163,16	183,36	155,06	122,44	153,04	2,18
Urtica dioica	Stinging nettle	Root	109,01	104,90	110,43	109,07	100,86	93,37	29,81	137,84	156,50	224,44	199,41	185,63	208,82	100,88
Symphytum officinale	Comfrey	Root	105,62	120,61	114,45	99,46	97,54	85,58	47,79	106,24	143,47	166,37	191,76	123,39	156,54	67,34
Althaea officinalis	Marshmallow	Root	100,22	93,79	80,71	82,49	87,56	78,29	67,86	104,25	113,39	96,02	194,59	160,52	183,97	139,71
Panax ginseng	Ginseng	Root	107,05	107,98	125,88	147,96	173,19	164,01	4,13	106,76	94,66	117,42	82,44	99,07	116,80	199,71
Harpagophytum procumbens	Devil's claw	Root	125,75	116,09	135,75	145,66	142,70	144,23	3,68	115,10	127,64	160,49	180,30	147,55	163,53	275,03

Dioscorea villosa	Yam	Root	113,51	108,51	103,64	60,24	29,52	11,04	3,27	104,81	102,20	163,08	224,08	138,65	169,18	26,64
Calendula officinalis	Marigold	Flower	60,30	40,34	58,30	3,49	2,91	2,77	2,19	91,73	78,85	269,59	354,55	0,00	0,00	0,00
Chelidonium majus	Celandine	Root	120,88	105,15	123,10	127,73	170,41	111,32	1,42	99,40	157,00	190,57	292,73	271,52	280,41	0,00
Verbena officinalis	Common vervain	Whole plant	96,47	88,48	97,54	86,98	86,77	85,59	47,65	127,02	140,00	208,28	208,79	233,55	236,51	374,51
Pimpinella anisum	Anise	Fruit/berry/ seed	116,49	118,67	116,39	114,10	108,54	84,98	3,36	136,25	169,95	236,67	250,31	286,11	344,07	20,08
Viscum album	European mistletoe	Whole plant	125,75	138,13	162,97	160,01	166,89	174,64	76,09	93,86	114,91	100,53	170,99	242,81	373,37	625,53
Digitalis purpurea	Common foxglove	Leaf	86,35	49,52	28,50	9,67	2,50	1,49	2,18	146,70	344,09	313,09	296,48	11,91	3,20	1,60
Primula officinalis	Common cowslip	Root	122,30	155,54	145,79	116,90	57,14	26,43	3,07	125,37	140,65	188,90	819,19	85,17	12,98	8,91
Juniperus communis	Common juniper	Fruit/berry/ seed	106,49	131,13	121,88	77,99	3,10	2,84	1,55	859,81	863,37	888,08	759,89	18,31	9,85	0,00
Aesculus hippocastanum	Horse-chestnut	Fruit/berry/ seed	110,81	80,80	92,60	7,84	5,11	5,00	3,20	123,23	134,19	212,88	1537,04	102,98	77,65	58,38
Primula vulgaris	Common primrose	Root	48,96	26,97	24,47	13,49	12,19	8,12	1,52	115,35	604,33	1193,00	674,91	45,73	58,06	21,03
