# Supplementary figures and images for: Screening of herbal extracts for TLR2- and TLR4-dependent anti-inflammatory effects
Source: PLoS One. 2018 Oct 11;13(10):e0203907. doi: 10.1371/journal.pone.0203907 (PMC6181297; doi:10.1371/journal.pone.0203907)

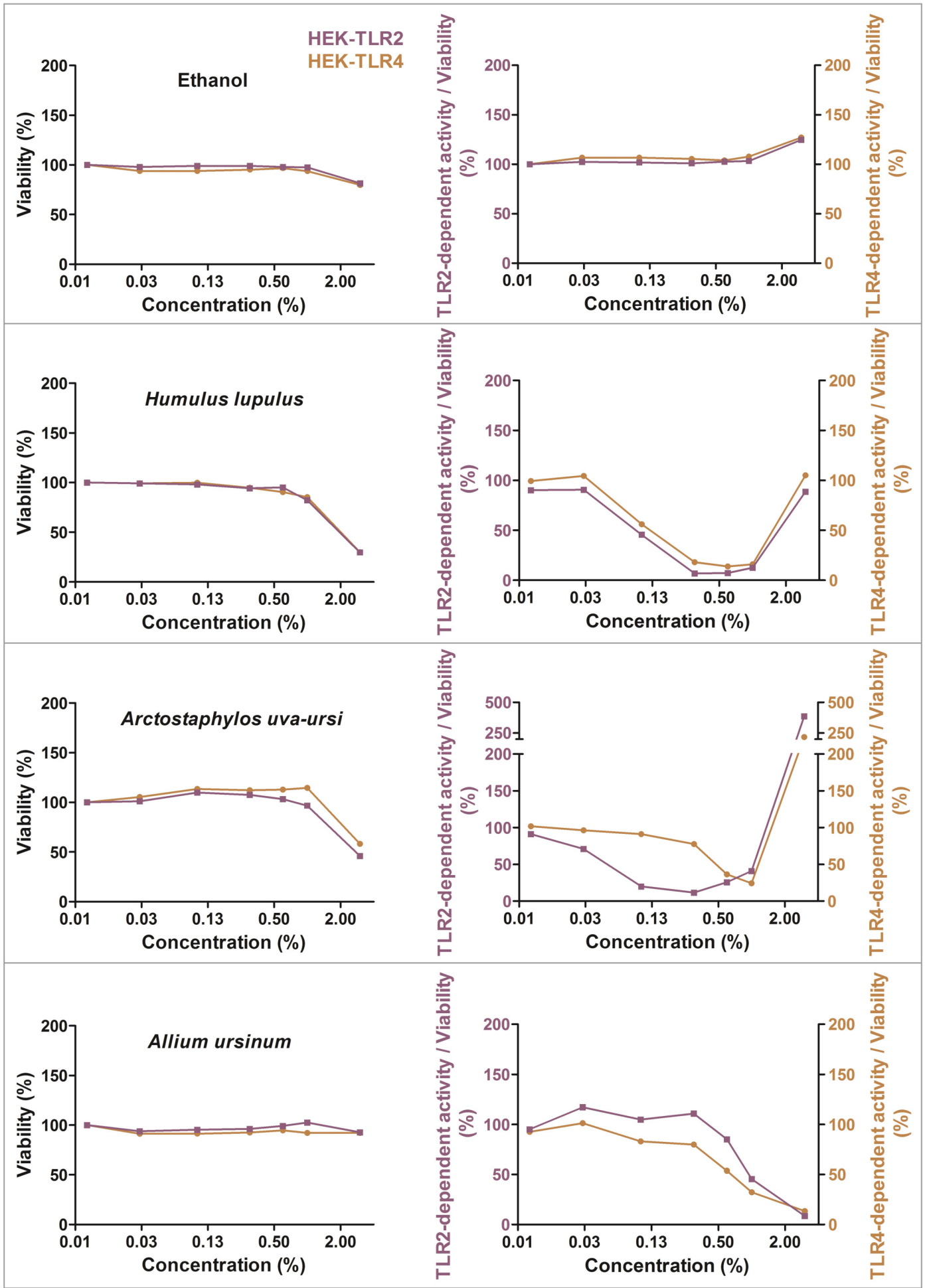

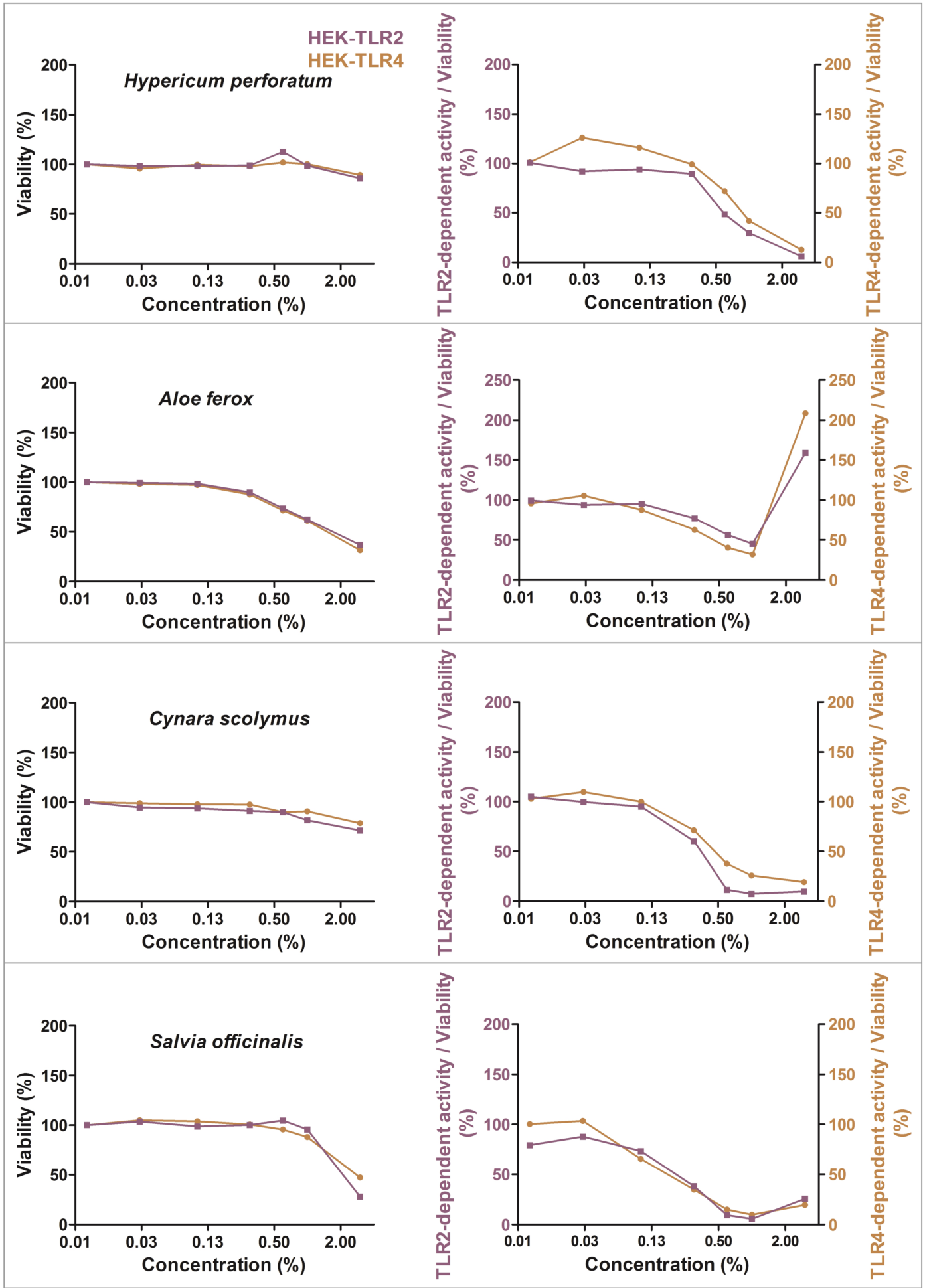

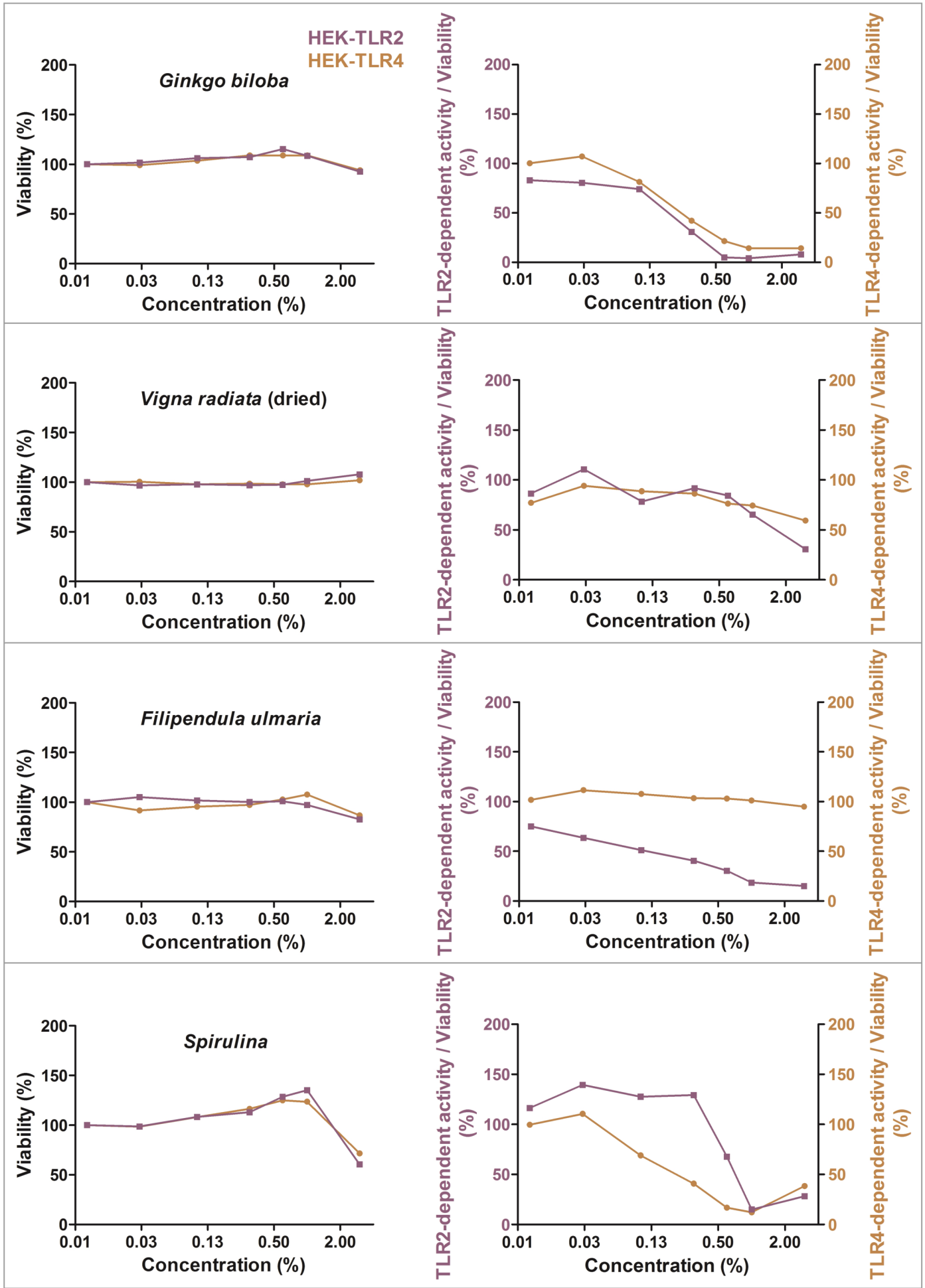

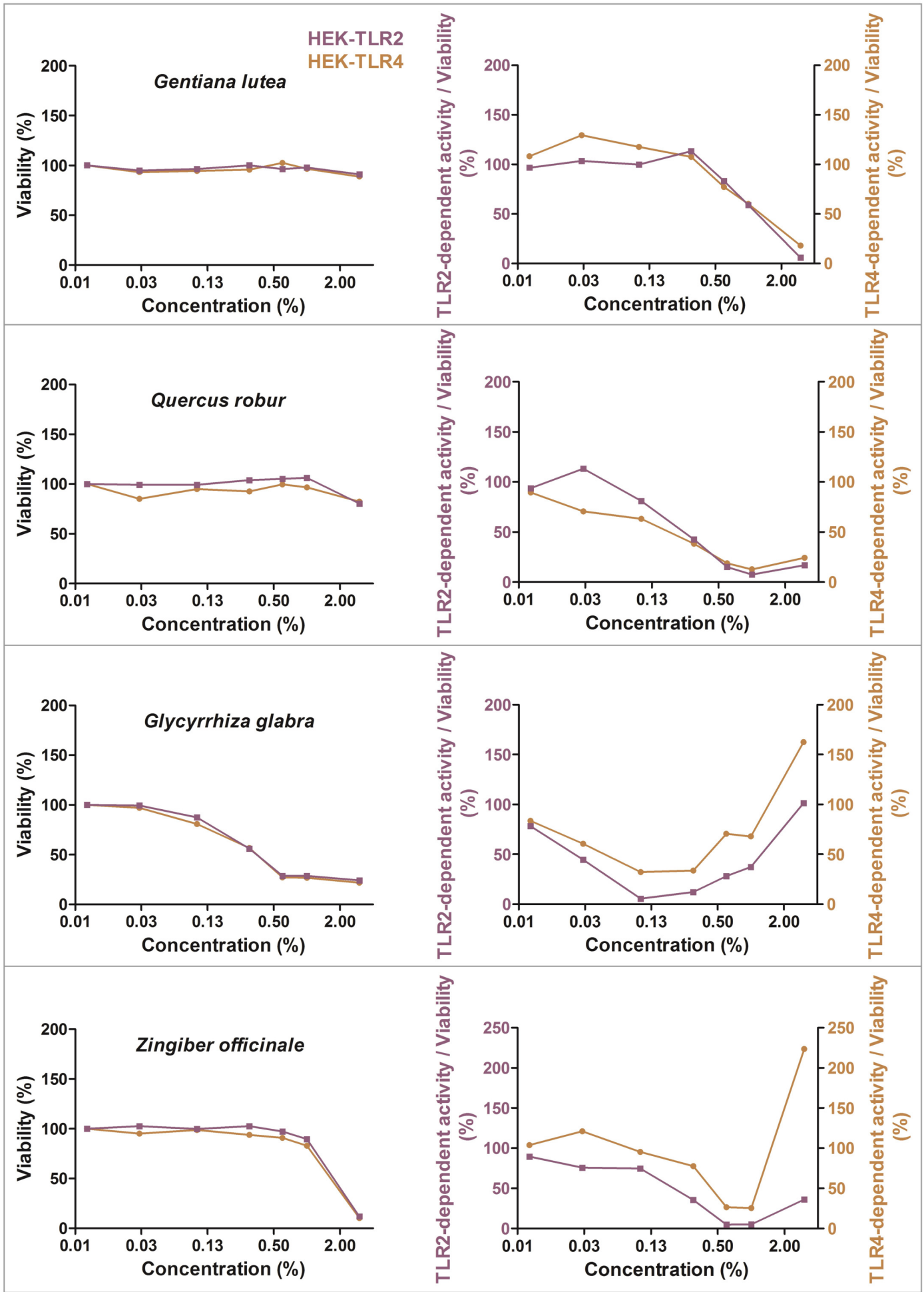

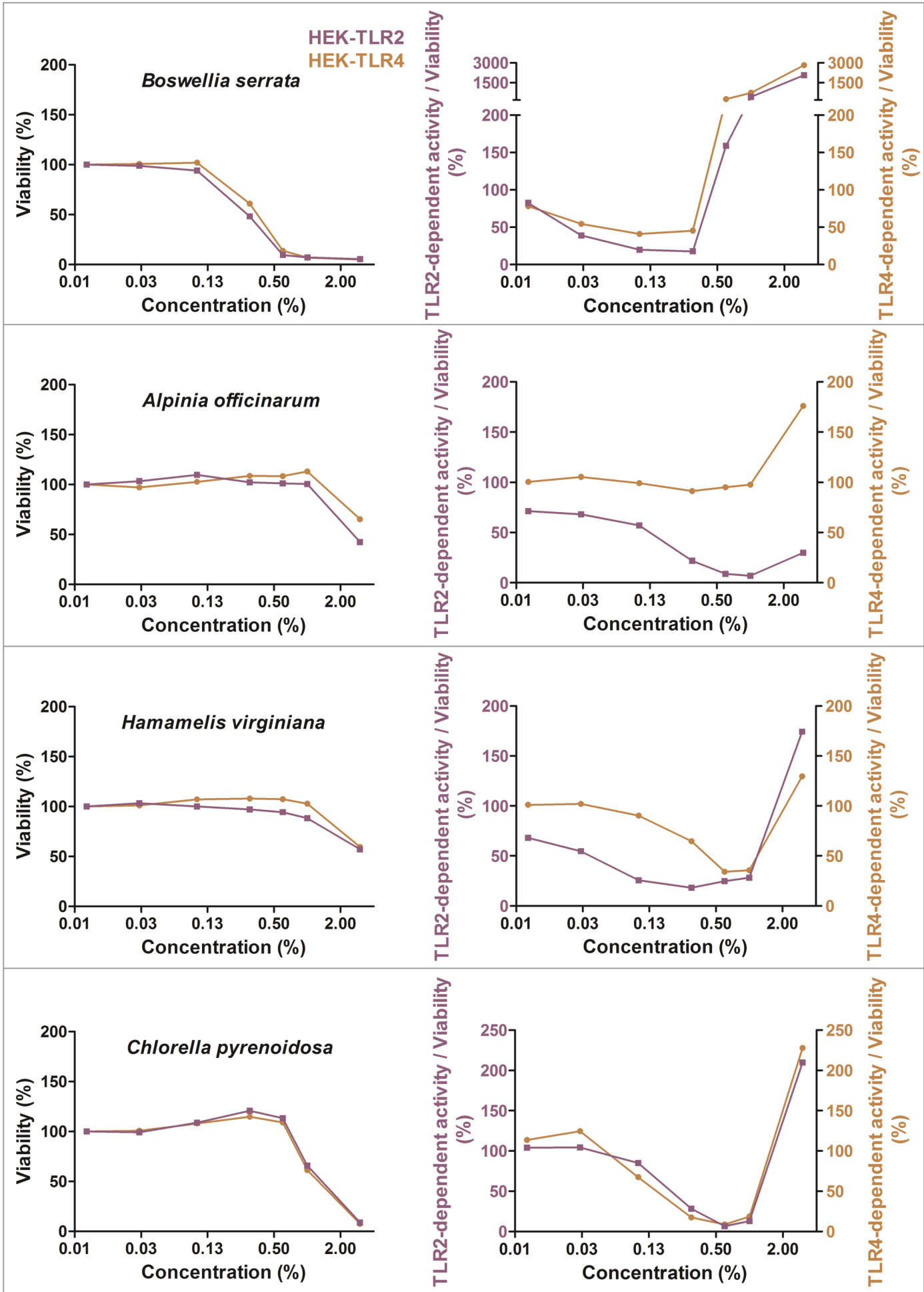

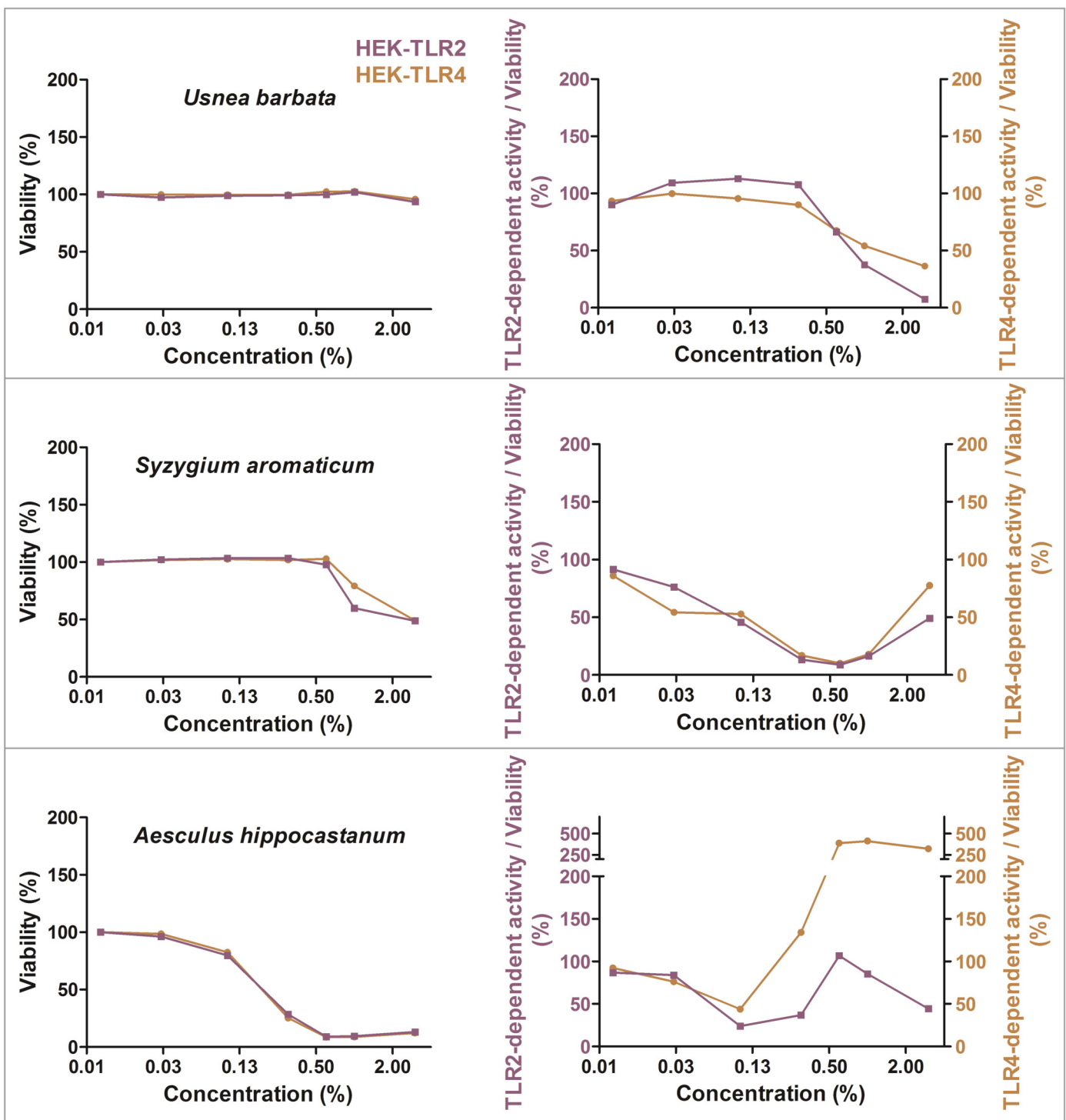

Supplement: S3 Fig — HEK-TLR2 cells (purple) and HEK-TLR4 cells (orange) were incubated with extracts (the five extracts with highest anti-inflammatory potential are displayed in Figs 5 and 6) or vehicle (70% ethanol), followed by stimulation of HEK-TLR2 cells with Pam2CSK4 or HEK-TLR4 cells with LPS-EB Ultrapure. Viability was measured using the Alamar Blue Assay was normalized to the respective negative control. TLR2 and TLR4 receptor activity were measured using SEAP production was normalized to ethanol-treated cells. Data are displayed as viability (%) in the left graphs and receptor activity divided by viability (%) in the right graphs. Data represents means (n≥2). (PDF) [file pone.0203907.s004.pdf]
