# Supplementary material for: Screening of herbal extracts for TLR2- and TLR4-dependent anti-inflammatory effects
Source: PLoS One. 2018 Oct 11;13(10):e0203907. doi: 10.1371/journal.pone.0203907 (PMC6181297; doi:10.1371/journal.pone.0203907)
Supplement: S4 Fig — HEK-TLR2 or HEK-TLR4 cells were incubated with extracts in different concentrations or vehicle (70% ethanol), followed by stimulation of HEK-TLR2 cells with Pam2CSK4 or HEK-TLR4 cells with LPS-EB Ultrapure. Viability was measured using Alamar Blue Assay was normalized to the negative control. TLR2 and TLR4 receptor activity were measured using SEAP production and were normalized to ethanol-treated cells. Data are displayed as receptor activity divided by normalized viability. Data represents means (n≥2). (PDF) [file pone.0203907.s005.pdf]

Inflammatory scale: anti-inflammatory — pro-inflammatory

Toxicity scale: viability < 75%

Latin name	Common English name	Used part	HEK-TLR2 reporter cell line							HEK-TLR4 reporter cell line						
			0.01%	0.03%	0.1%	0.3%	0.6%	1%	3%	0.01%	0.03%	0.1%	0.3%	0.6%	1%	3%
Ethanol control			100,00	102,45	101,84	101,03	102,55	103,43	124,76	100,00	106,70	106,82	105,37	103,93	107,97	127,11
Castanea sativa	Sweet chestnut	Leaf	20,06	17,83	9,78	11,82	19,94	28,56	118,82	24,28	17,36	12,78	15,09	20,87	32,77	161,75
Cinchona pubescens	Cinchona	Bark	100,53	94,50	77,03	65,89	25,61	13,17	22,78	96,97	92,46	86,34	75,11	48,95	37,82	32,81
Cinnamomum verum	Cinnamon	Bark	100,40	100,11	105,47	65,88	36,23	22,34	37,65	100,75	97,62	94,69	59,98	27,50	18,82	32,43
Salix alba	White willow	Bark	102,12	100,18	85,03	42,14	20,67	17,65	73,17	100,01	85,03	67,85	40,08	34,16	24,12	52,75
Rheum palmatum	Rhubarb	Root	96,48	87,24	59,40	36,53	13,11	8,17	24,68	89,65	89,52	64,85	40,62	20,74	16,27	38,65
Humulus lupulus	Hops	Flower	90,21	90,80	45,52	6,84	7,25	12,55	88,75	99,42	104,49	56,15	18,10	13,97	15,99	105,06
Arctostaphylos uva-ursi	Bearberry	Leaf	91,42	71,09	20,16	11,86	25,75	41,19	385,83	101,93	96,18	91,06	77,63	36,71	24,40	217,02
Allium ursinum	Wild garlic	Leaf	95,05	117,18	104,97	110,95	85,31	45,41	8,55	92,64	101,32	83,11	79,90	53,77	32,22	13,36
Hypericum perforatum	St John's wort	Whole plant	100,82	92,09	94,08	89,60	48,53	29,53	6,00	101,20	125,93	115,86	99,00	72,14	41,77	12,66
Aloe ferox	Aloe	Whole plant	99,50	94,02	95,36	76,96	56,33	45,12	158,90	95,70	105,77	87,57	62,60	40,30	32,00	208,70
Cynara scolymus	Artichoke	Leaf	104,96	99,81	95,14	60,38	11,43	7,45	9,75	102,92	109,70	99,86	71,37	37,63	25,76	19,01
Salvia officinalis	Salvia	Leaf	79,45	87,83	73,41	38,26	9,48	5,77	25,80	100,28	103,61	65,58	34,90	14,99	9,98	19,63
Ginkgo biloba	Ginkgo	Leaf	83,06	80,39	74,11	30,88	4,92	4,06	7,87	100,24	107,01	81,26	42,13	21,39	14,24	14,18
Vigna radiata	Mung bean (dried)	Fruit/berry/seed	86,58	110,71	78,43	91,74	84,41	65,29	30,75	77,04	94,29	88,78	86,30	76,44	74,46	59,49
Filipendula ulmaria	Meadowsweet	Flower	75,12	63,58	51,28	40,60	30,35	18,59	15,08	101,71	111,43	107,58	103,43	102,99	101,11	94,91
Spirulina	Spirulina	Whole cyanobacteria	116,49	139,54	127,71	129,36	67,85	15,01	28,28	99,69	110,62	69,12	40,91	16,95	12,20	38,50
Gentiana lutea	Gentian	Root	96,77	103,65	99,90	113,40	83,38	59,14	5,82	108,21	129,60	117,81	107,54	77,28	60,19	18,10
Quercus robur	English oak	Bark	93,72	113,22	81,11	42,60	15,04	7,52	16,84	89,53	70,65	63,23	38,30	18,82	12,82	24,23
Glycyrrhiza glabra	Liquorice	Root	78,30	44,63	5,56	12,09	28,06	37,27	101,27	83,64	60,59	32,18	33,77	70,66	67,87	162,46
Zingiber officinale	Ginger	Root	89,51	75,69	74,77	35,53	4,69	5,18	35,84	103,89	120,85	95,36	77,68	26,45	25,34	223,46
Boswellia serrata	Frankincense	Juice/resin	82,46	38,96	19,81	17,73	159,07	432,24	2076,63	78,29	54,43	40,86	45,48	288,07	744,11	2813,02
Alpinia officinarum	Galangal	Root	71,36	68,16	57,03	21,85	8,95	6,85	29,83	100,45	105,34	99,27	91,33	95,04	97,57	176,07
Hamamelis virginiana	Witch hazel	Leaf	68,01	54,73	25,63	18,14	24,82	28,25	174,32	101,02	102,08	90,19	64,82	34,21	35,64	129,53
Chlorella pyrenoidosa	Chlorella	Whole algae	103,97	104,34	84,84	28,14	6,63	12,96	209,74	113,66	124,42	67,31	17,39	8,74	18,75	227,74
Usnea barbata	Barber's itch	Whole plant	90,08	109,48	113,03	107,82	66,29	37,56	7,49	93,50	99,92	95,64	90,15	67,63	54,02	36,36
Syzygium aromaticum	Clove	Flower	91,40	76,21	45,89	13,37	8,91	16,47	49,18	86,11	54,39	52,82	16,99	10,06	18,12	77,65
Aesculus hippocastanum	Horse-chestnut	Fruit/berry/seed	86,76	83,98	24,02	36,87	106,93	85,46	44,41	92,43	76,26	43,91	134,14	388,13	413,57	323,76
